# Supplementary material for: Synthetic yeast chromosome XI design provides a testbed for the study of extrachromosomal circular DNA dynamics
Source: Cell Genom. 2023 Nov 9;3(11):100418. doi: 10.1016/j.xgen.2023.100418 (PMC10667340; doi:10.1016/j.xgen.2023.100418)
Supplement: Document S1. Figures S1–S13 and Tables S1, S5, and S6 [file mmc1.pdf]

Cell Genomics, Volume *3*

## Supplemental information

# Synthetic yeast chromosome XI design provides a testbed for the study of extrachromosomal circular DNA dynamics

**Benjamin A. Blount, Xinyu Lu, Maureen R.M. Driessen, Dejana Jovicevic, Mateo I. Sanchez, Klaudia Ciurkot, Yu Zhao, Stephanie Lauer, Robert M. McKiernan, Glen-Oliver F. Gowers, Fiachra Sweeney, Viola Fanfani, Evgenii Lobzaev, Kim Palacios-Flores, Roy S.K. Walker, Andy Hesketh, Jitong Cai, Stephen G. Oliver, Yizhi Cai, Giovanni Stracquadanio, Leslie A. Mitchell, Joel S. Bader, Jef D. Boeke, and Tom Ellis**

## Supplemental Information

**Contents:**

**Figure S1 – page 1**
**Figure S2 – page 2**
**Figure S3 – page 3**
**Figure S4 – page 3**
**Figure S5 – page 4**
**Figure S6 – page 5**
**Figure S7 – page 6**
**Figure S8 – page 7**
**Figure S9 – page 8**
**Figure S10 – page 9**
**Figure S11 – page 10**
**Figure S12 – page 10**
**Figure S13 – page 11**
**Table S1 – page 12**
**Table S5 – pages 13-14**
**Table S6 – pages 15-16**

## Supplemental Figures

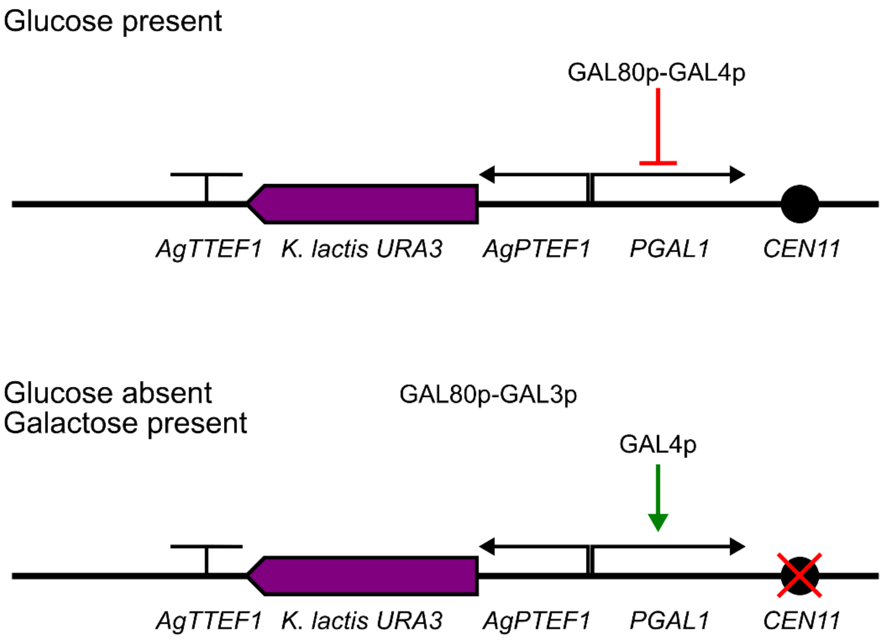


**Figure S1: Schematic of *CEN11* functional disruption by the *CEN11** construct - related to Figure 1.** Overview of the *CEN11** locus under glucose repression and galactose induction. In the absence of glucose and the presence of galactose, transcription from the *GAL1* promoter (*PGAL1*) disrupts the function of *CEN11*, leading to chromosome loss through miss-segregation in mitosis. *AgTTEF1* is the *TEF1* terminator from *Ashbya gossypii*, *K. lactis URA3* is the *URA3* coding sequence from *Kluyveromyces lactis* and *AgPTEF1* is the *TEF1* promoter from *A. gossypii*. The red bar indicates transcriptional repression, the green arrow represents transcriptional activation and the red cross represents disruption of centromere function.

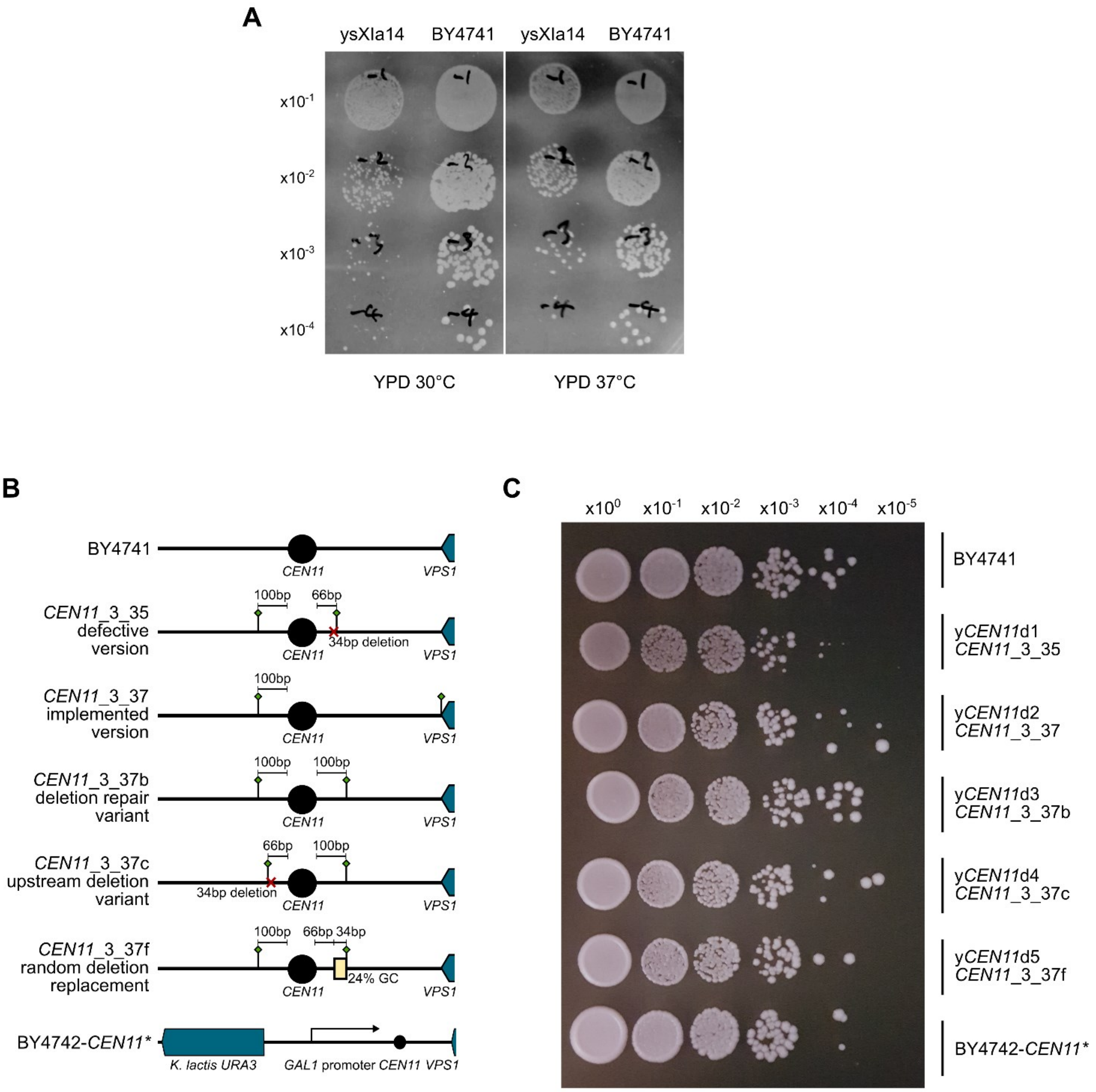


**Figure S2: CEN11_3_35 causes a growth defect - related to Figure 2.** (A) Growth spot assays of ysXla14 and BY4741 parental control on YPD agar after 2 days. Incubation temperatures and spot dilution factors are indicated on the image. (B) Overview of the *CEN11* locus variants generated in this study. Green diamonds denote loxPsym sites. (C) Growth spot assays of BY4742 strains with different *CEN11* locus variants on YPD after 3 days growth at 37 °C. Strain names and *CEN11* variant numbers (where appropriate) are shown to the right. Spot dilution factors are shown above the image.

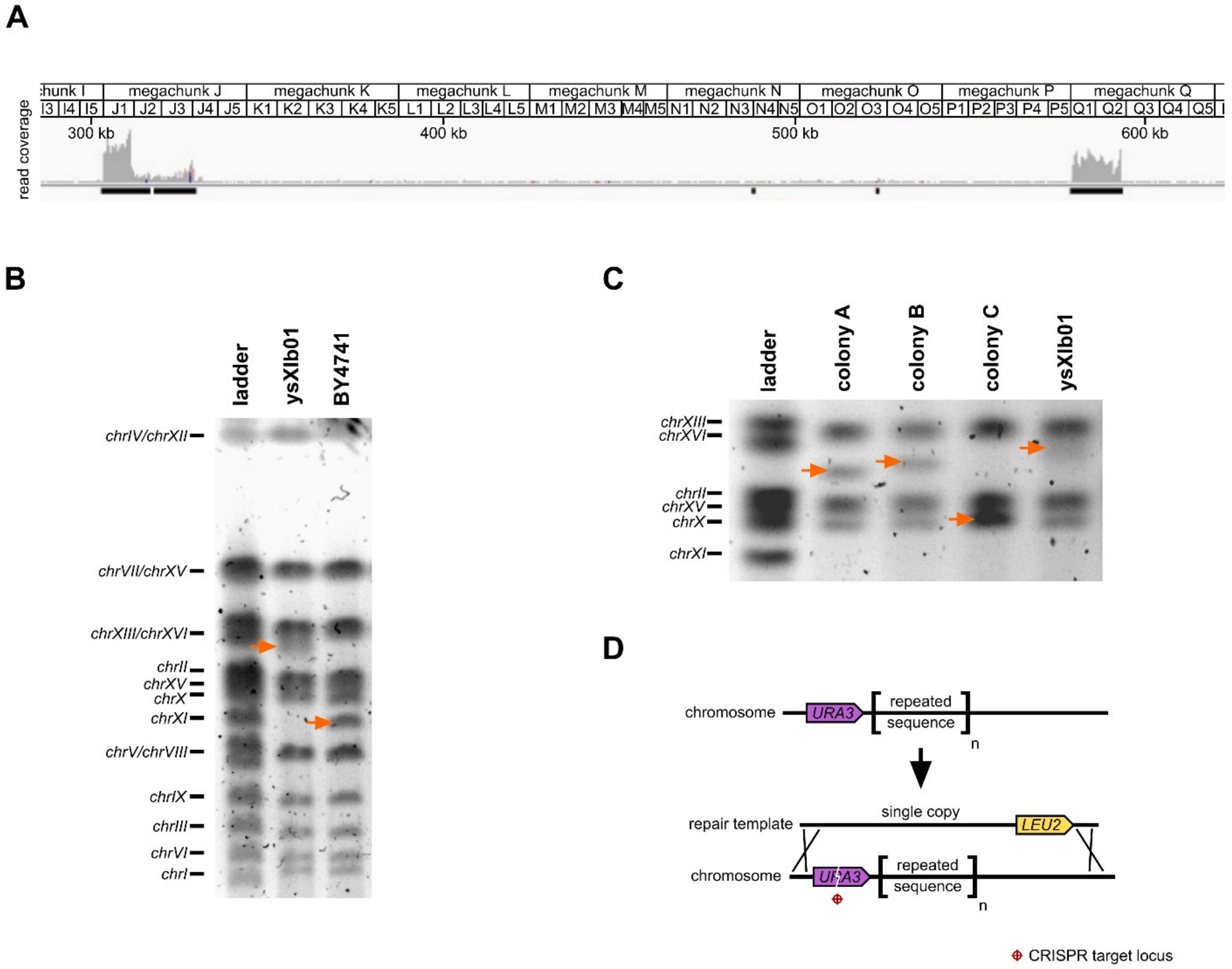


**Figure S3: Repeat regions were condensed using CRISPR/Cas9 – related to Figure 2.** (A) Read coverage depth from short read genome sequencing over a section of *synXI*_9.01 isolated from ysXIb01. Annotated boxes above the graph indicate the corresponding synthetic chunk and megachunk regions. (B) PFGE electrophoresis gel of genomic DNA isolated from ysXIb01 and BY4741 showing increased size of *synXI* in ysXIb01. The ladder is 0.2-2.2 Mb *S. cerevisiae* ladder (Bio-Rad). Orange arrows show the inferred positions of *chrXI* and *synXI*. (C) PFGE electrophoresis gel of genomic DNA isolated from ysXIb01 and colonies isolated after the first round of CRISPR-mediated megachunk J repeat condensation. Colony C is deduced to have the most condensation of synXI repeats. Orange arrows show the inferred position of *synXI*. (D) Strategy to condense the repeat sequence in the megachunk Q region. *URA3* is inserted upstream of the repeats before being targeted by CRISPR-Cas9 with a single copy of the region as repair template.

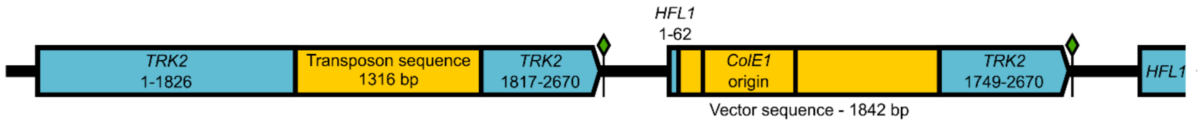


**Figure S4: Schematic of the sequence insertions at the *YKR050W/TRK2* locus in ysXI_9.09 – related to Figure 2**. Blue boxes represent yeast CDS sequence, yellow boxes represent sequence of bacterial origin of replication, green diamonds represent loxPsym sites. Base coordinates given for *TRK2* and *HFL1* sequences are relative to the annotated start of the coding sequence in the wildtype strain.

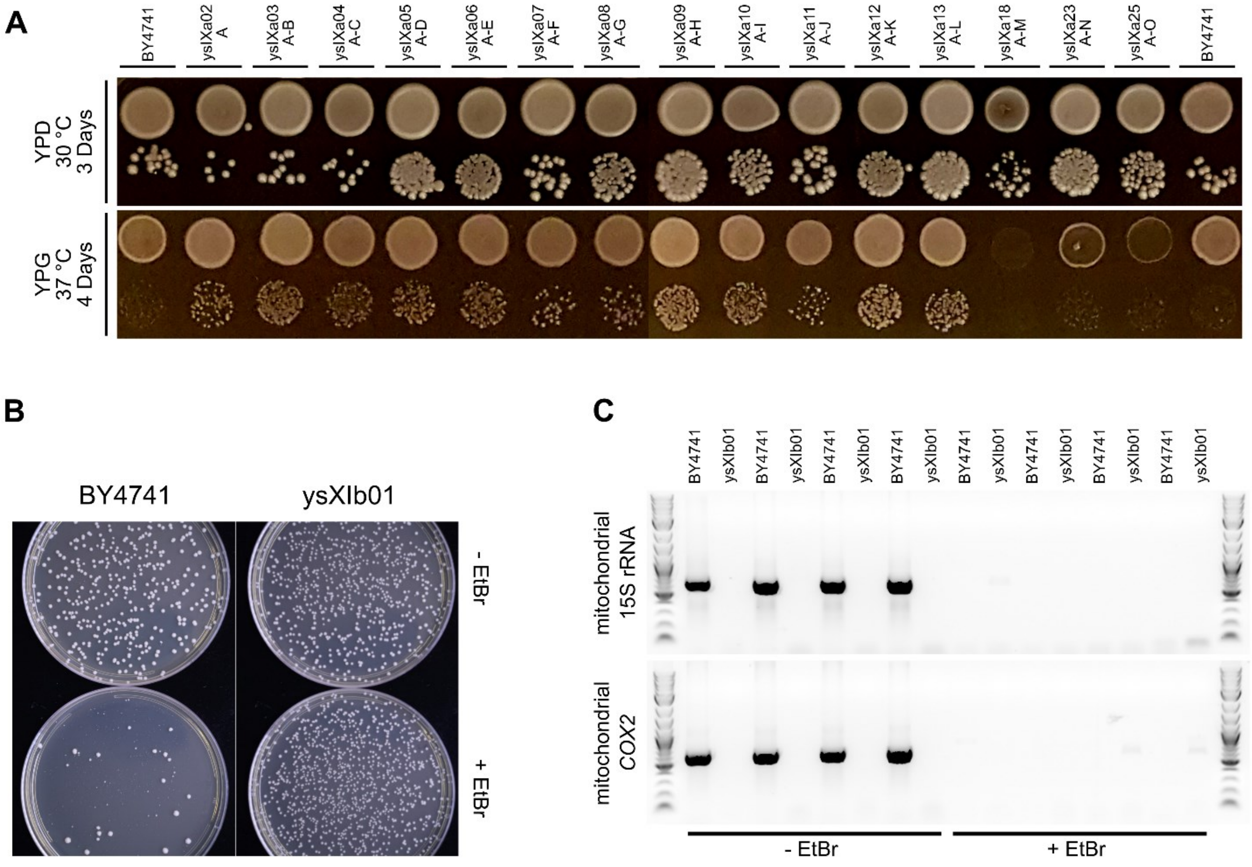


**Figure S5: ysXIb01 has a mitochondrial defect - related to Figure 3.** (A) Growth spot assays of *synXI* assembly intermediates following rounds of megachunk integration on YPD (glucose) and YPG (glycerol) to test respiratory function. For each strain and condition, the top spot is a $x10^{-1}$ dilution and the bottom spot is a $x10^{-3}$ dilution. (B) Diluted cells plated onto YPD with or without 24 hours prior exposure 10 µg ethidium bromide (EtBr). EtBr causes loss of the mitochondrial genome. (C) PCR assays targeting mitochondrial genomic DNA for amplification performed on genomic DNA harvested from colonies grown on YPD or on YPD + 10 µg EtBr. Product bands indicate the presence of mitochondrial genomic DNA. n=4 biological replicates per strain and per condition. PCR products are visualised on 1% agarose gel, with the gel image colour inverted. Ladder is 1 kb Plus DNA Ladder (New England Biolabs).

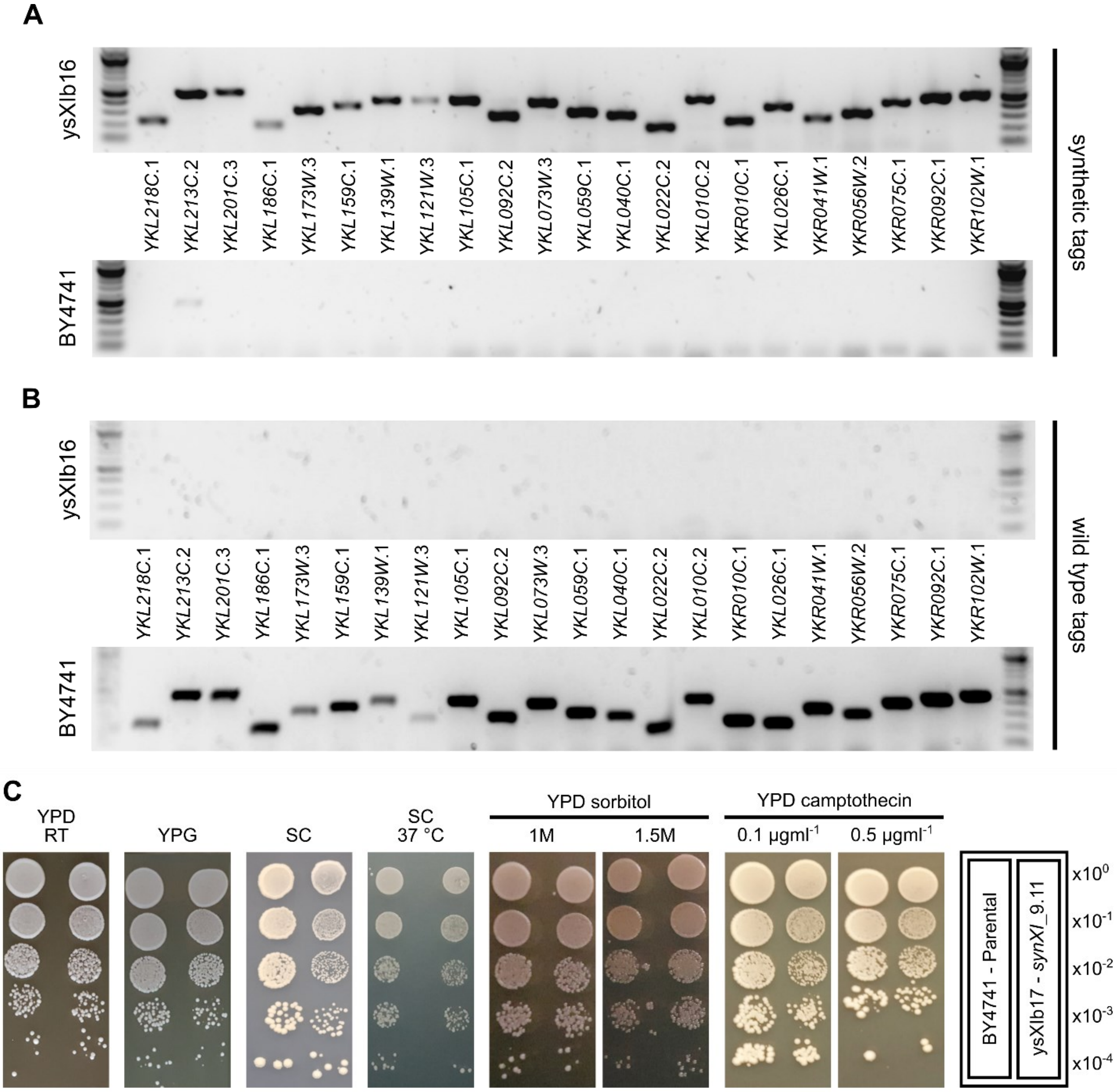


**Figure S6: Genotypic and phenotypic validation of synXI strains – related to Figure 4.** (A) Genomic DNA from ysXIb16 or a BY4741 control was amplified with synthetic PCRTag primer pairs. Product bands indicate the presence of synthetic DNA. (B) Genomic DNA from ysXIb16 or a BY4741 control was amplified with wild type PCRTag primer pairs. Product bands indicate the presence of wild type DNA. For panels A and B, PCR products were visualised on 1% agarose gel, with the colour inverted. PCRTag primer pair targets are labelled between the gel images, with target sequences listed in Table S4. Ladder is 1 kb Plus DNA Ladder (New England Biolabs). (C) Additional growth spot assays of ysXIb17 and a BY4741 parental control to assess cellular fitness. Serial dilutions of cultures were spotted as illustrated on the right. Unless otherwise indicated, plates were incubated at 30 °C.

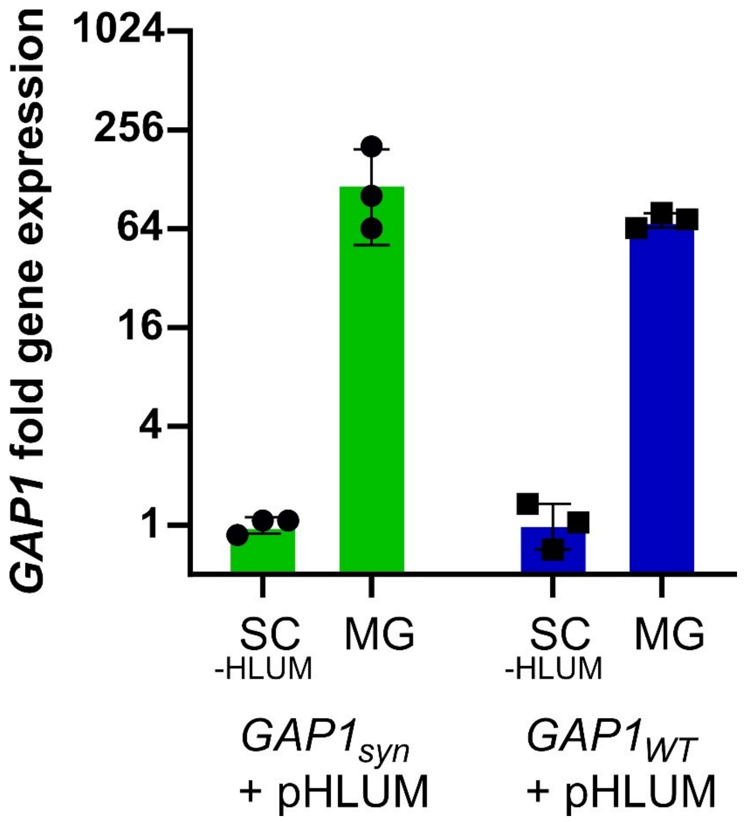


**Figure S7: *GAP1$_{syn}$* has similar transcription levels to *GAP1$_{WT}$* - related to Figure 5.** Quantification of *GAP1* transcript levels by qPCR. Strains BY4741 (*GAP1$_{WT}$*) and *GAP1syn* were transformed with pHLUM and grown for 16 hours in SC -His -Leu -Ura -Met (SC -HLUM) or MG (0.4 mM L-glutamine) before harvesting. *GAP1* transcripts were quantified by qPCR using ACT1 as a reference gene. Data is given relative to mean *GAP1* transcript abundance in *GAP$_{WT}$* +pHLUM cells grown in SC -His -Leu -Ura -Met. Experiments were performed in biological triplicate. Individual *GAP1$_{syn}$* data points are plotted as round dots, individual *GAP1$_{WT}$* data points are plotted as square dots, mean averages are denoted by bar height and error bars represent standard deviation.

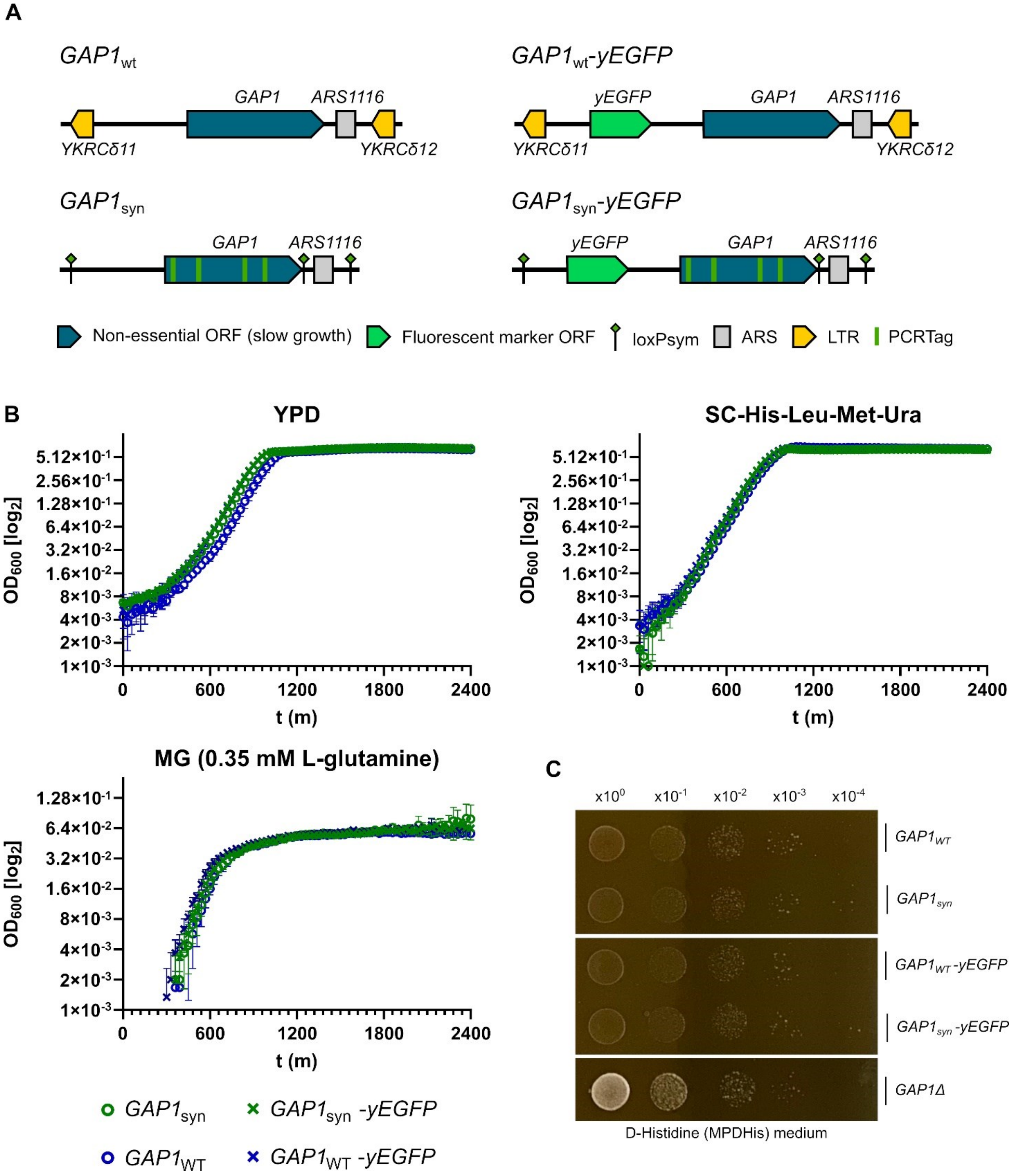


**Figure S8: *GAP1syn* produces functional Gap1 protein and does not impact growth - related to Figure 5.** (A) Structure of *GAP1* loci built and integrated into BY4741 to study eccDNA dynamics. (B) Graphs show growth of BY4741 strains with different variations of the *GAP1* locus in 96 well plates under rich (YPD), defined (SC-His-Leu-Met-Ura) and low-nitrogen (MG) media conditions at 30 °C. All strains had plasmid pHLUM, making them prototrophic. Mean $OD_{600}$ values from 3 biological replicates are plotted as circles, error bars represent standard deviation. (C) Spot growth assay on MDPHis medium containing D-histidine. Plates were incubated at 30 °C for 2 days. Image panels are all taken from a single image of one assay plate.

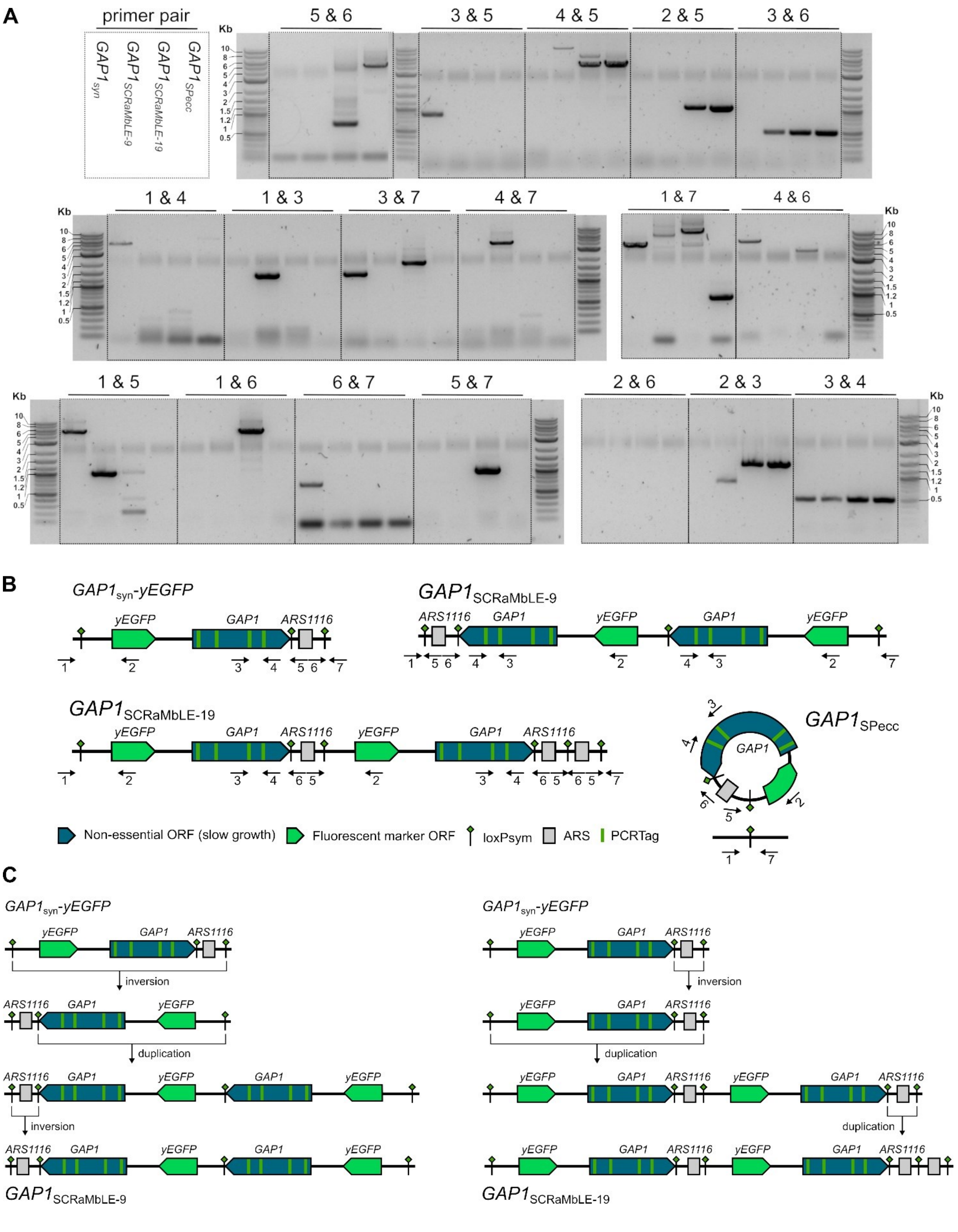


**Figure S9: SCRaMbLE rearrangements of the *GAP1$_{syn}$* locus were identified – related to Figure 5.** (A) Agarose gel electrophoresis analysis of diagnostic PCRs to determine *GAP1* locus structure of strains following SCRaMbLE of the *GAP1$_{syn}$-yEGFP* strain. Boxed sections of gel images denote samples with the same primer pairs, indicated above the boxes. Top left box shows lane loading order of each strain analysed in each boxed section. Primer pairs are numbered 1 = XL216, 2 = XL789, 3 = XL788, 4 = XL808, 5 = XL809, 6 = XL790, 7 = XL222. Primer sequences can be found in Table S3. Ladder is 1 kb Plus DNA Ladder (NEB). (B) Show's schematic overviews of the *GAP1* locus of *GAP1$_{syn}$-yEGFP* and the SCRaMbLE derived strains. Numbered arrows are indicative of the binding positions and direction of each of the primers used in diagnostic PCR. Arrow lengths and relative positions are not to scale. (C) Shows one possible mechanism each for the SCRaMbLE generation of strains *GAP1*$_{SCRaMbLE-9}$ and *GAP1*$_{SCRaMbLE-19}$. As the diagnostic methods used cannot confirm the specific SCRaMbLE events leading to each configuration, these are illustrative only.

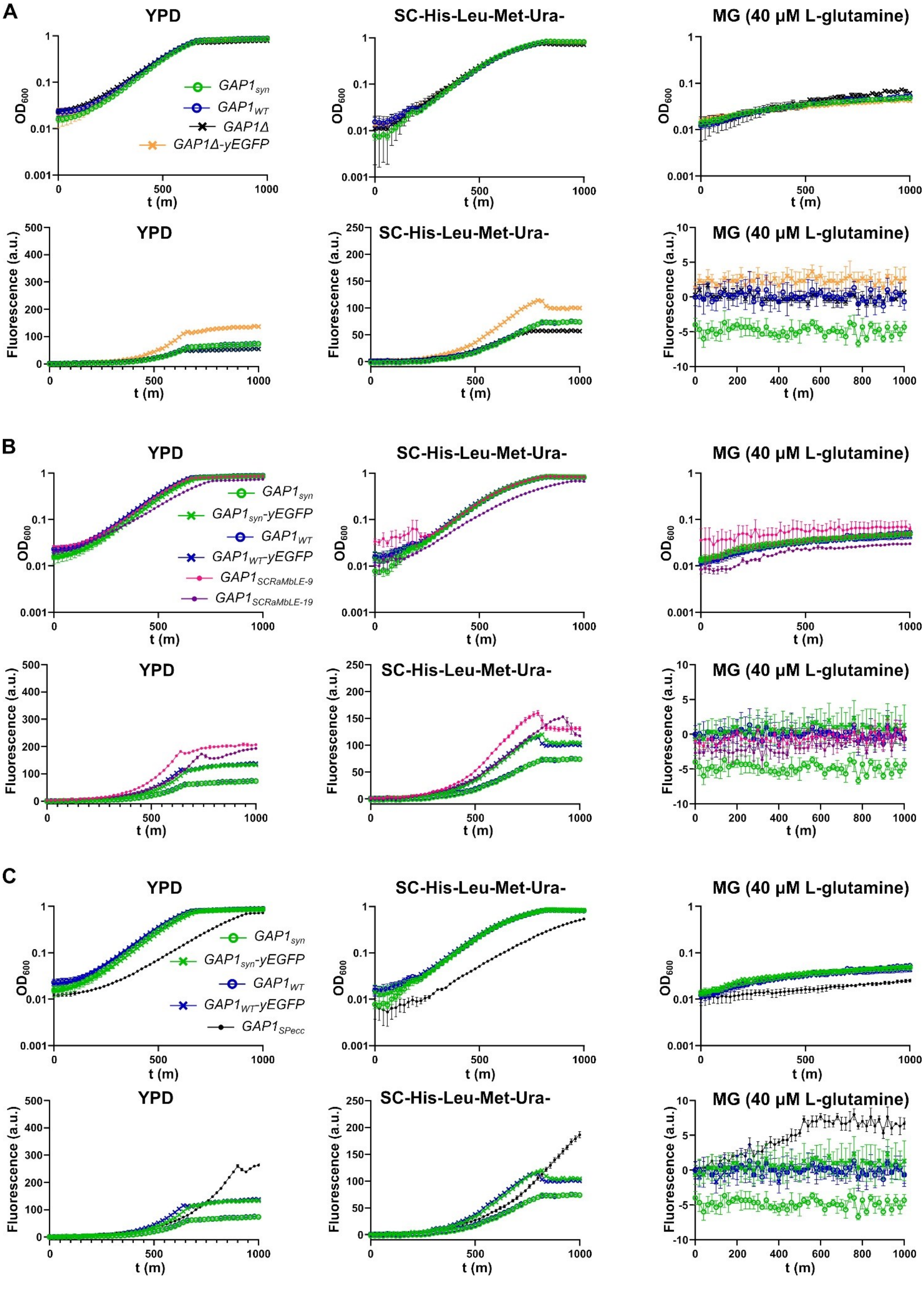


**Figure S10: $GAP1_{syn}$ locus variants were assessed for effects on growth - related to Figure 5.** Growth and population fluorescence of BY4741 cultures with different *GAP1* locus variants. (A) shows $GAP1_{syn}$, $GAP1_{WT}$, *GAP1Δ* and *GAP1Δ-yEGFP cultures, (B)* shows $GAP1_{syn}$, $GAP1_{syn}$*-yEGFP*, $GAP1_{WT}$, $GAP1_{WT}$*-yEGFP*, $GAP1_{SCRaMbLE-9}$ *and* $GAP1_{SCRaMbLE-19}$, *and (C) shows* $GAP1_{syn}$, $GAP1_{syn}$*-yEGFP*, $GAP1_{WT}$, $GAP1_{WT}$*-yEGFP and* $GAP1_{SPecc}$. Media-blanked mean $OD_{600}$ and fluorescence values from 3 biological replicates are plotted for each culture, error bars represent standard deviation. Assay growth medium is stated above each graph and assays took place at 30 °C. The y-axis has a $log_{10}$ scale for $OD_{600}$ and a linear scale for fluorescence.

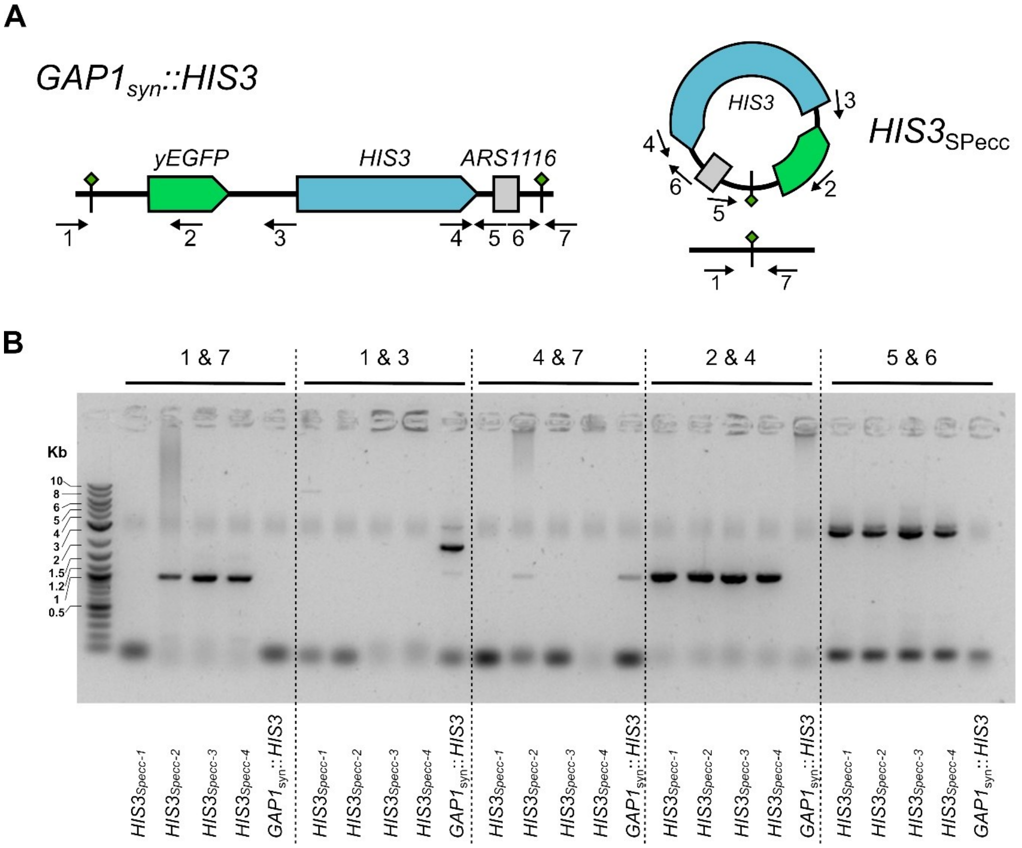


**Figure S11: *HIS3* SPeccs were confirmed - related to Figure 6.** (A) Show's schematic overviews of the *GAP1::HIS3* locus of BY4741-*HIS3-yEGFP* (labelled *GAP1*$_{syn}$::*HIS3*) and the SCRaMbLE derived HIS3$_{SPecc}$ strains. Numbered arrows are indicative of the binding positions and direction of each of the primers used in diagnostic PCR. Arrow lengths and relative positions are not to scale. (B) Agarose gel electrophoresis analysis of diagnostic PCRs to confirm *HIS3* SPecc formation following SCRaMbLE of the BY4741-*HIS3-yEGFP* strain. Primer pairs are indicated above the gel and strain analysed in each lane is indicated below the gel. Primer pairs are numbered 1 = XL216, 2 = XL789, 3 = XL1243, 4 = XL1244, 5 = XL809, 6 = XL790, 7 = BB585. Primer sequences can be found in Table S3. Ladder is 1 kb Plus DNA Ladder (NEB).

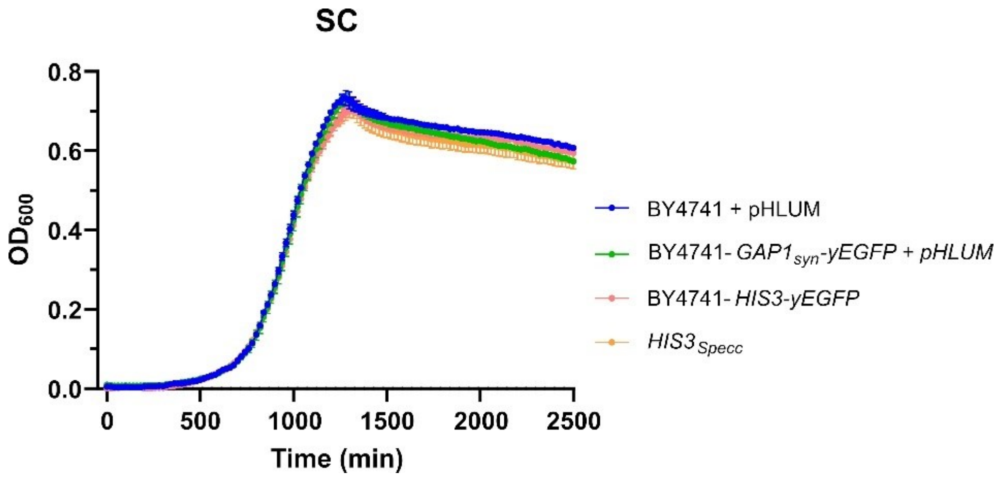


**Figure S12: *HIS3* SPeccs do not affect growth in SC medium - related to Figure 6.** Growth of BY4741-*HIS3*$_{SPecc}$ and parental strains in defined media with histidine (SC) at 30 °C. Mean $OD_{600}$ values from 3 biological replicates are plotted as circles, error bars represent standard deviation.

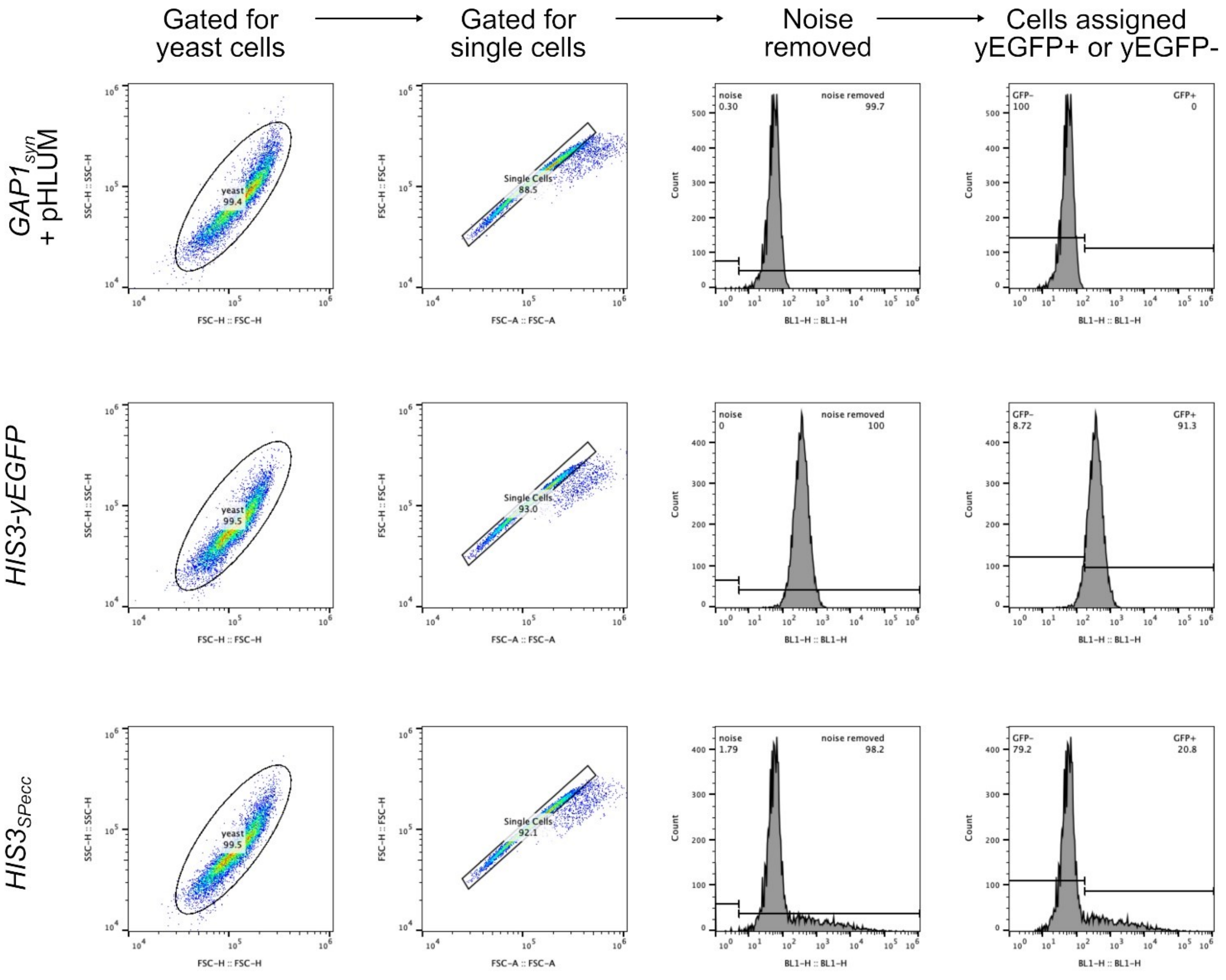


**Figure S13: Gating distinguished the yEGFP status of cells – related to Figure 6.** Examples of the gating process used to process raw flow cytometry data to generate the ratios shown in Figure 6F. Shown here is one set of data from each culture grown in SC (histidine +) medium.

## Supplemental tables

| Design element | Synthesized design *synXI*_3.34 | Final sequence *synXI*_9.11 |
|---|---|---|
| TAG stop codons replaced | 68 | 71 |
| PCRTags | 914 | 900 |
| loxP sites | 199 | 196 |
| tRNAs removed | 15 | 15 |
| introns removed | 9 | 9 |
| Assembly restriction sites | 87 | 80 |
| chunks - synthesis section | 87 | - |
| megachunks - integration sections | 18 | - |
| Assembled size (bp)* | 659,617 | 659,107 |

**Table S1 – Summary of design changes implemented in *synXI* – related to Figure 1.** Asterisk denotes that this number does not take into account *in vivo* fluctuation in telomere sequence length.

| Purpose | Target | Target sequence | Retargeting primer | Retargeting primer sequence | Repair template |
|---|---|---|---|---|---|
| M Debugging | M1 - *YKL005C_2_WT_R* | GGAAACGTCAAAGGAGGCAG[AGG] | BB392 | CTGCCTCCTTTGACGTTTCCaaagtcccattcgccacccg | M1 chunk |
| M Debugging | M2 - *YKR001C_2_WT_F* | ACTGGCGCCTTACTAGACGA[TGG] | BB394 | TCGTCTAGTAAGGCGCCAGTaaagtcccattcgccacccg | M2 chunk |
| M Debugging | M3 - *YKR006C_1_WT_R* | TATTGAAGAAGGCACTAACG[AGG] | BB396 | CGTTAGTGCCTTCTTCAATAaaagtcccattcgccacccg | M3 chunk |
| M Debugging | M4 - *YKR010C_2_WT_F* | CTAGTGGTGCTCATAGTCCC[AGG] | BB398 | GGGACTATGAGCACCACTAGaaagtcccattcgccacccg | M4 chunk |
| M Debugging | M5 - *YKR013W_1_WT_F* | TGAGGATTGAGTGCTTGAGG[CGG] | BB400 | CCTCAAGCACTCAATCCTCAaaagtcccattcgccacccg | M5 chunk |
| *CEN11** replacement | *KlURA3* 3' region | CTTCGGGAATCGCCTATCAG[CGG] | BB875 | CTGATAGGCGATTCCCGAAGaaagtcccattcgccacccg | *CEN11* variant BB877/BB878 PCR product |
| *CEN11** replacement | *YKR001C_2_WT_F* | ACTGGCGCCTTACTAGACGA[TGG] | BB394 | TCGTCTAGTAAGGCGCCAGTaaagtcccattcgccacccg | M2 chunk |
| *HIS3-TR2* removal | HIS3 | TTTTTACTCCACGCGCCAGT[AGG] | BB417 | ACTGGCGCGTGGAGTAAAAAaaagtcccattcgccacccg | *his3*Δ1 as BB418/BB419 PCR product |
| O integration patching | *YKR051W_1_WT_R* | ATTCTTGTACACATATCCAG[AGG] | BB390 | CTGGATATGTGTACAAGAATaaagtcccattcgccacccg | O3-O4 chunk ligation product |
| R5 *URA3* removal | *URA3* | GCAGACATTACGAATGCGCA[CGG] | BB486 | TGCGCATTCGTAATGTCTGCaaagtcccattcgccacccg | Neighbouring sequence |
| Mitotic crossover | O5 *LEU2* | GAAAAAAATAAAAATGGAGT[AGG] | BB457 | ACTCCATTTTTATTTTTTTCaaagtcccattcgccacccg | Sister chromatid |
| J repeat condensation | *LEU2* | CCAGCGCCTCATCTGGAAGT[GGG] | BB573 | ACTTCCAGATGAGGCGCTGGaaagtcccattcgccacccg | Megachunk J |
| J repeat condensation | *kanMX* | ATGAAGGAGAAAACTCACCG[AGG] | BB574 | CGGTGAGTTTTCTCCTTCATaaagtcccattcgccacccg | Megachunk J |
| J repeat condensation | J1 vector backbone sequence (5') | ACTGGAAAGCGGGCAGTGAG[CGG] | BB604 | CTCACTGCCCGCTTTCCAGTaaagtcccattcgccacccg | Neighbouring sequence |
| J repeat condensation | J1 vector backbone sequence (3') | CTATAGGGCGAATTGGCGGA[AGG] | BB603 | TCCGCCAATTCGCCCTATAGaaagtcccattcgccacccg | Neighbouring sequence |
| Q repeat condensation | *URA3* | GCAGACATTACGAATGCGCA[CGG] | BB486 | TGCGCATTCGTAATGTCTGCaaagtcccattcgccacccg | Megachunk Q |
| *TRK2* insertion removal | *TRK2* insertion - transposon sequence | GCTCAAAATTTATTCACACA[TGG] | BB736 | TGTGTGAATAAATTTTGAGCaaagtcccattcgccacccg | Neighbouring sequence |
| Q debugging | P5 *URA3* | GAAAAGCTTGCGGTAGTGAA[GGG] | BB783 | TTCACTACCGCAAGCTTTTCaaagtcccattcgccacccg | Megachunk Q |
| Q debugging | *YKR077W_1_WT_R* | GTTTGCATTCGGAGTTCTTG[GGG] | BB704 | CAAGAACTCCGAATGCAAACaaagtcccattcgccacccg | Megachunk Q |

| Purpose | Target | Target sequence | Retargeting primer | Retargeting primer sequence | Repair template |
|---|---|---|---|---|---|
| Q debugging | *YKR084C _2_WT_F* | AGAGTTGTTCTTA GAAAGGA[CGG] | BB705 | TCCTTTCTAAGAACAACTCTaaagtcc cattcgccacccg | Megachunk Q |
| Glycerol locus replacement | *YKR084C _1_syn_F* | GCTAACGCCGATC AACGTAG[TGG] | BB757 | CTACGTTGATCGGCGTTAGCaaagtc ccattcgccacccg | Debug colony 2 locus BB768/BB775 PCR product |
| Glycerol locus replacement | *YKR086W _1_syn_R* | CAATGGAGTTAAT TGACCTG[AGG] | BB778 | CAGGTCAATTAACTCCATTGaaagtcc cattcgccacccg | Debug colony 2 locus BB768/BB775 PCR product |
| *PRP16* stop swap | *PRP16* TAG | TCTTCCCATAAACT AAAAAA[AGG] | BB785 | TTTTTTTAGTTTATGGGAAGaaagtccc attcgccacccg | Q3 chunk BB738/BB788 PCR product |
| *GAP1*$_{syn}$ generation | *YKRCδ11* | TCACCCATTTGTC AAGATAA[TGG] | XL498 | TTTGGTCTCGCGCATCACCCATTTG TCAAGATAAGTTTTAGAGCTAGAAA TAGCAAGTTA | synthetic *GAP1* locus amplicon |
| | | | XL499 | TTTGGTCTCGTTGATATAAGCCCTG CGCAAGCCCGGAATCGAAC | |
| *GAP1*$_{syn}$ generation | *YKRCδ12* | GGGCTTATATCAA GATCTGT[TGG] | XL500 | TTTGGTCTCCTCAAGATCTGTGTTT TAGAGCTAGAAATAGCAAGTTA | synthetic *GAP1* locus amplicon |
| | | | XL501 | TTTGGTCTCCGGATTGCGCAAGCC CGGAATCGAAC | |
| *yEGFP* insertion | sequence upstream of *GAP1* | TTTGTCGAAGATA TTCGACG[AGG] | XL502 | AGATTTTGTCGAAGATATTCGACG | *PPFY1-yEGFP-TCYC1* |
| | | | XL503 | AAACCGTCGAATATCTTCGACAAA | |
| Replacing *GAP1* with *HIS3* | sequence upstream of *GAP1* | GTTGTAGGTTACG TAAGCAG[GGG] | XL1176 | TTTGGTCTCGCGCAGTTGTAGGTTA CGTAAGCAGGTTTTAGAGCTAGAAA TAGCAAGTTA | *HIS3* |
| | | | XL1182 | TTTGGTCTCGTTAGATTAATGATGC GCAAGCCCGGAATCGAAC | |
| Replacing *GAP1* with *HIS3* | sequence downstrea m of *GAP1* | TCATTAATCTAAAG AAAAAA[GGG] | XL1177 | TTTGGTCTCCCTAAAGAAAAAAGTT TTAGAGCTAGAAATAGCAAGTTA | *HIS3* |
| | | | XL1501 | TTTGGTCTCCGGATTGCGCAAGCC CGGAATCGAAC | |

**Table S5 - CRISPR/Cas9 target sequences and gRNA retargeting primers – related to STAR methods.** Target sequence Protospacer Adjacent Motifs are shown in square brackets. Retargeting primer sequences are shown with the pWS082 binding sequence in lowercase and the retargeting sequence in uppercase.

| Version | Strain(s) | Comment | Details |
|---|---|---|---|
| *synXI*_3.34 | N/A | | Final design by BioStudio |
| *synXI*_3.36 | N/A | | TAG stop codons in *YKL006C-A*, *YKR004C* and *YKR005C* recoded to TAA |
| *synXI*_3.37 | N/A | | *CEN11* right arm loxPsym site moved to 3 bp 3' of *YKR001C* |
| *synXI*_9.01 | ysXIb01 | Initial synXI assembly | Missing loxPsym sites: 526668-526701 (*YKR052C*). WT PCRTags: 466241-466268 (*YKR016W*_1_F), 493836-493863 (*YKR029C*_2_R),493911-493938 (*YKR029C*_1_F), 536959-536986 (*YKR054C*_6_R). Point mutations causing amino acid changes: 37065 T>C (*YKL210W*), 448094 G>C (*YKR008W*). Structural variations: 303238-328404 (repeated sequence), 523727-525128 (insertions/duplication at *YKR050W*), 578748-591038 (repeated sequence). |
| *synX*I_9.02 | ysXIb02 | Markers inserted flanking megachunk J repeated sequence | Missing loxPsym sites: 526668-526701 (*YKR052C*). WT PCRTags: 466241-466268 (*YKR016W*_1_F), 493836-493863 (*YKR029C*_2_R),493911-493938 (*YKR029C*_1_F), 536959-536986 (*YKR054C*_6_R). Point mutations causing amino acid changes: 37065 T>C (*YKL210W*), 448094 G>C (*YKR008W*). Structural variations: 303238-328404 (repeated sequence), 523727-525128 (insertions/duplication at *YKR050W*), 578748-591038 (repeated sequence). Marker gene insertions: 303694 (*YKL0069W*::*LEU2*), 334347 (*YKL053C*::*kanMX4*). |
| *synXI*_9.03 | ysXIb03 | Megachunk J repeated sequence copy number reduced - respiratory growth defect present | Missing loxPsym sites: 526668-526701 (*YKR052C*). WT PCRTags: 466241-466268 (*YKR016W*_1_F), 493836-493863 (*YKR029C*_2_R),493911-493938 (*YKR029C*_1_F), 536959-536986 (*YKR054C*_6_R). Point mutations causing amino acid changes: 37065 T>C (*YKL210W*), 448094 G>C (*YKR008W*). Structural variations: 303238-311650 (repeated sequence with pMA-RQ insertion at 311649), 523727-525128 (insertions/duplication at *YKR050W*), 578748-591038 (repeated sequence). Marker gene insertions: 343787 (*YKL048C*::*URA3*). |
| *synXI*_9.04 | ysXIb04 | *URA3* marker removed - respiratory growth defect present | Missing loxPsym sites: 526668-526701 (*YKR052C*). WT PCRTags: 466241-466268 (*YKR016W*_1_F), 493836-493863 (*YKR029C*_2_R),493911-493938 (*YKR029C*_1_F), 536959-536986 (*YKR054C*_6_R). Point mutations causing amino acid changes: 37065 T>C (*YKL210W*), 448094 G>C (*YKR008W*). Structural variations: 303238-311650 (repeated sequence with pMA-RQ insertion at 311649), 523727-525128 (insertions/duplication at *YKR050W*), 578748-591038 (repeated sequence). |
| *synXI*_9.05 | ysXIb05 | Megachunk J repeated sequence removed - respiratory growth defect present | Missing loxPsym sites: 526668-526701 (*YKR052C*). WT PCRTags: 466241-466268 (*YKR016W*_1_F), 493836-493863 (*YKR029C*_2_R),493911-493938 (*YKR029C*_1_F), 536959-536986 (*YKR054C*_6_R). Point mutations causing amino acid changes: 37065 T>C (*YKL210W*), 448094 G>C (*YKR008W*). Structural variations: 523727-525128 (insertions/duplication at *YKR050W*), 578748-591038 (repeated sequence). |
| *synXI*_9.06 | ysXIb06 | *URA3* inserted upstream of megachunk Q repeated sequence - respiratory growth defect present | Missing loxPsym sites: 526668-526701 (*YKR052C*). WT PCRTags: 466241-466268 (*YKR016W*_1_F), 493836-493863 (*YKR029C*_2_R),493911-493938 (*YKR029C*_1_F), 536959-536986 (*YKR054C*_6_R). Point mutations causing amino acid changes: 37065 T>C (*YKL210W*), 448094 G>C (*YKR008W*). Structural variations: 523727-525128 (insertions/duplication at *YKR050W*), 578748-591038 (repeated sequence). Marker gene insertions: 579173 (*YKR077W*::*URA3*). |
| *synXI*_9.08 | ysXIb08 | Megachunk Q repeated sequence removed - respiratory growth defect present | Missing loxPsym sites: 526668-526701 (*YKR052C*). WT PCRTags: 466241-466268 (*YKR016W*_1_F), 493836-493863 (*YKR029C*_2_R),493911-493938 (*YKR029C*_1_F), 536959-536986 (*YKR054C*_6_R). Point mutations causing amino acid changes: 37065 T>C (*YKL210W*), 448094 G>C (*YKR008W*). Structural variations: 523727-525128 (insertions/duplication at *YKR050W*). |
| *synXI*_9.09 | ysXIb09 | *TRK2*/*YKR050W* insertions/duplication removed - | Missing loxPsym sites: 526668-526701 (*YKR052C*). WT PCRTags: 466241-466268 (*YKR016W*_1_F), 493836-493863 (*YKR029C*_2_R),493911-493938 (*YKR029C*_1_F), 536959-536986 (*YKR054C*_6_R). Point mutations causing |

| Version | Strain(s) | Comment | Details |
|---|---|---|---|
| | | respiratory growth defect present | amino acid changes: 37065 T>C (*YKL210W*), 448094 G>C (*YKR008W*). |
| *synXI*_9.10 | ysXIb10, ysXIb11, ysXIb12 | Respiratory growth defect fixed | Remaining TAG stop codons: 597913 A>G (*YKR086W*). Missing loxPsym sites: 526668-526701 (*YKR052C*), 593898-593931 (*YKR085C*),598033-598066 (*YKR087C*). WT PCRTags: 466241-466268 (*YKR016W*_1_F), 493836-493863 (*YKR029C*_2_R),493911-493938 (*YKR029C*_1_F), 536959-536986 (*YKR054C*_6_R), 592963-592990 (*YKR084C*_1_F), 593239-593266 (*YKR084C*_1_R), 593956-593983 (*YKR085C*_1_F), 594292-594319 (*YKR085C*_1_R), 595453-595480 (*YKR086W*_2_F), 595684-595711 (*YKR086W*_2_R), 595912-595939 (*YKR086W*_3_F), 596266-596293 (*YKR086W*_3_R), 596749-596773 (*YKR086W*_1_F), 596989-597016 (*YKR086W*_1_R). Point mutations causing amino acid changes: 37065 T>C (*YKL210W*), 448094 G>C (*YKR008W*). |
| *synXI*_9.11 | ysXIb13, ysXIb14, ysXIb16, ysXIb17 | Final version | Missing loxPsym sites: 526668-526701 (*YKR052C*), 593898-593931 (*YKR085C*),598033-598066 (*YKR087C*). WT PCRTags: 466241-466268 (*YKR016W*_1_F), 493836-493863 (*YKR029C*_2_R),493911-493938 (*YKR029C*_1_F), 536959-536986 (*YKR054C*_6_R), 592963-592990 (*YKR084C*_1_F), 593239-593266 (*YKR084C*_1_R), 593956-593983 (*YKR085C*_1_F), 594292-594319 (*YKR085C*_1_R), 595453-595480 (*YKR086W*_2_F), 595684-595711 (*YKR086W*_2_R), 595912-595939 (*YKR086W*_3_F), 596266-596293 (*YKR086W*_3_R), 596749-596773 (*YKR086W*_1_F), 596989-597016 (*YKR086W*_1_R). Point mutations causing amino acid changes: 37065 T>C (*YKL210W*), 448094 G>C (*YKR008W*). |

**Table S6 - *synXI* chromosome versions – related to STAR methods.** For *in vivo* chromosome versions, variations from *synXI*_3.37 are listed.
