# Supplementary material for: Synthetic yeast chromosome XI design provides a testbed for the study of extrachromosomal circular DNA dynamics
Source: Cell Genom. 2023 Nov 9;3(11):100418. doi: 10.1016/j.xgen.2023.100418 (PMC10667340; doi:10.1016/j.xgen.2023.100418)
Supplement: Document S2. Article plus supplemental information [file mmc9.pdf]

Cell Genomics

Article

Synthetic yeast chromosome XI design provides a testbed for the study of extrachromosomal circular DNA dynamics

Graphical abstract

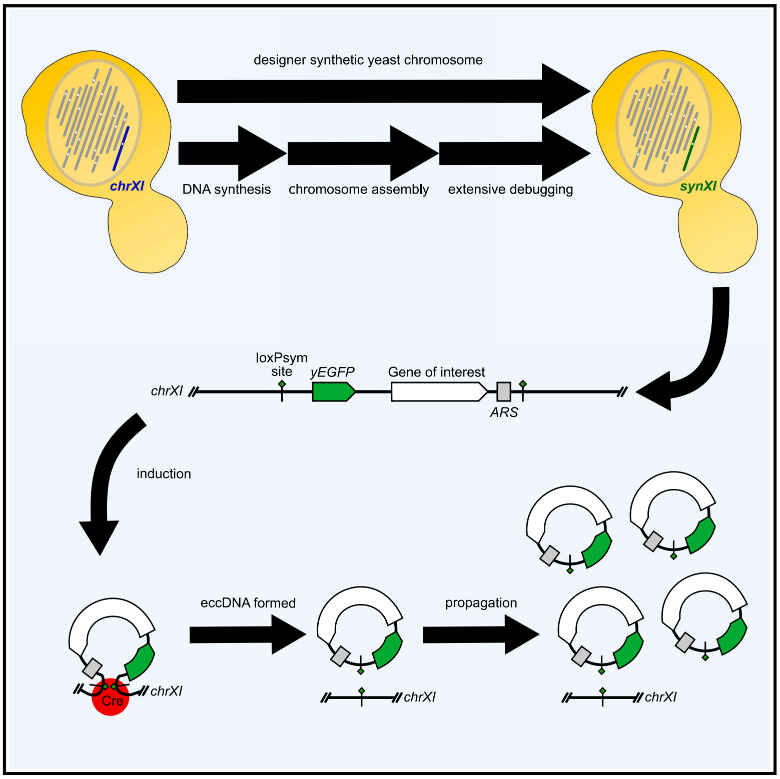

Authors

Benjamin A. Blount, Xinyu Lu, Maureen R.M. Driessen, ..., Joel S. Bader, Jef D. Boeke, Tom Ellis

Correspondence

benjamin.blount@nottingham.ac.uk (B.A.B.), t.ellis@imperial.ac.uk (T.E.)

In brief

Blount et al. describe completion of the synthetic *Saccharomyces cerevisiae* chromosome XI and identify redesigns to non-coding regions as essential to achieve strain fitness equivalent to the wild type. Using the *GAP1* locus, they demonstrate that the synthetic design can enable generation of extrachromosomal circular DNAs through an inducible recombination-based method.

Highlights

- The 660-kb yeast synthetic chromosome XI (synXI) was assembled *in vivo*
- Extensive debugging fixed defects mapping to redesigned noncoding DNA
- Cells with debugged synXI have fitness and transcription profiles equal to wild type
- Sc2.0-formatted chromosomal loci can be used to form extrachromosomal circles

Blount et al., 2023, Cell Genomics *3*, 100418
November 8, 2023 © 2023 The Author(s).
https://doi.org/10.1016/j.xgen.2023.100418

CellPress

Cell Genomics

CellPress OPEN ACCESS

Article

Synthetic yeast chromosome XI design provides a testbed for the study of extrachromosomal circular DNA dynamics

Benjamin A. Blount,[1,2,3,*] Xinyu Lu,[1,2] Maureen R.M. Driessen,[1,2] Dejana Jovicevic,[1,2] Mateo I. Sanchez,[1,2] Klaudia Ciurkot,[1,2] Yu Zhao,[4] Stephanie Lauer,[4] Robert M. McKiernan,[1,2,5] Glen-Oliver F. Gowers,[1,2] Fiachra Sweeney,[1,5] Viola Fanfani,[6] Evgenii Lobzaev,[6,7] Kim Palacios-Flores,[8] Roy S.K. Walker,[9] Andy Hesketh,[10] Jitong Cai,[11] Stephen G. Oliver,[10] Yizhi Cai,[6,12] Giovanni Stracquadanio,[6] Leslie A. Mitchell,[4] Joel S. Bader,[11] Jef D. Boeke,[4,13] and Tom Ellis[1,2,14,*]

[1]Imperial College Centre for Synthetic Biology, Imperial College London, London, UK
[2]Department of Bioengineering, Imperial College London, London, UK
[3]School of Life Sciences, University of Nottingham, Nottingham, UK
[4]Institute for Systems Genetics and Department of Biochemistry and Molecular Pharmacology, NYU Langone Health, New York, NY, USA
[5]Department of Life Sciences, Imperial College London, London, UK
[6]School of Biological Sciences, The University of Edinburgh, Edinburgh, UK
[7]School of Informatics, The University of Edinburgh, Edinburgh, UK
[8]Laboratorio Internacional de Investigación sobre el Genoma Humano, Universidad Nacional Autónoma de México, Querétaro, México
[9]School of Engineering, Institute for Bioengineering, The University of Edinburgh, Edinburgh, UK
[10]Department of Biochemistry, University of Cambridge, Cambridge, UK
[11]Department of Biomedical Engineering, Whiting School of Engineering, Johns Hopkins University, Baltimore, MD, USA
[12]Manchester Institute of Biotechnology, University of Manchester, Manchester, UK
[13]Department of Biomedical Engineering, NYU Tandon School of Engineering, Brooklyn, NY, USA
[14]Lead contact
*Correspondence: benjamin.blount@nottingham.ac.uk (B.A.B.), t.ellis@imperial.ac.uk (T.E.)
https://doi.org/10.1016/j.xgen.2023.100418

SUMMARY

We describe construction of the synthetic yeast chromosome XI (*synXI*) and reveal the effects of redesign at non-coding DNA elements. The 660-kb synthetic yeast genome project (Sc2.0) chromosome was assembled from synthesized DNA fragments before CRISPR-based methods were used in a process of bug discovery, redesign, and chromosome repair, including precise compaction of 200 kb of repeat sequence. Repaired defects were related to poor centromere function and mitochondrial health and were associated with modifications to non-coding regions. As part of the Sc2.0 design, loxPsym sequences for Cre-mediated recombination are inserted between most genes. Using the *GAP1* locus from chromosome XI, we show that these sites can facilitate induced extrachromosomal circular DNA (eccDNA) formation, allowing direct study of the effects and propagation of these important molecules. Construction and characterization of *synXI* contributes to our understanding of non-coding DNA elements, provides a useful tool for eccDNA study, and will inform future synthetic genome design.

INTRODUCTION

Our rapidly improving understanding of DNA function, along with our ability to design and build large DNA constructs, has led to us being able to create synthetic genomes assembled from chemically synthesized DNA designed *in silico*.[1,2] Designing and assembling synthetic genomes provides opportunities to assess our current understanding of how DNA sequence and structure underpin cellular properties and behavior. Altering a DNA sequence, even when preserving encoded amino acid sequences, can affect how a gene is transcribed[3] and how mRNA is localized,[4] processed,[5] and translated[6] and alter spatial localization,[7] 3D interactions of genomic DNA,[8] and interactions with nuclear DNA-associated proteins.[9] Changes predicted to have no functional effect can lead to unexpected phenotypes, presenting an opportunity to uncover the underlying cause and refine our understanding. When this process occurs at a genomic scale, the scope for learning more about how DNA functions within a cell, from the base pair to genome level, is considerable.

The synthetic yeast genome project, Sc2.0, is an international collaboration to build the first eukaryotic synthetic genome, that of *Saccharomyces cerevisiae*. The Sc2.0 genome contains many design features that probe eukaryotic genome biology and will yield strains with encoded abilities not found in nature. Perhaps the most immediately useful feature is incorporation of loxPsym

This is an open access article under the CC BY license (http://creativecommons.org/licenses/by/4.0/).

recombinase target sites in the 3′ untranslated region (UTR) of almost all nonessential genes as well as at certain "landmark sites," where elements such as repeated DNAs or tRNA genes have been deleted by design.[10] These loxPsym sites enable a process of on-demand combinatorial chromosome rearrangement, synthetic chromosome rearrangement and modification by loxPsym-mediated evolution (SCRaMbLE). The diversity of gene content and arrangements generated by SCRaMbLE in a population of cells with Sc2.0 chromosomal DNA is vast,[11] and these synthetic diversified populations also show wide phenotypic variation. By isolating "SCRaMbLEd" cells from a population with phenotypes of interest, synthetic genetic changes resulting in desirable qualities can be identified. These can include properties desirable for biotechnology, such as enhanced growth on an alternative feedstock,[12] improved product biosynthesis,[13] or resistance to adverse growth conditions.[14] The SCRaMbLE system has also been used as a driver of random gene loss in efforts to determine the content of minimal or reduced eukaryotic genomes.[15]

In addition to the SCRaMbLE system, the Sc2.0 genome has many other design features.[16] These include gene recoding to remove all instances of the TAG stop codon and incorporating synonymous mutation watermarks, dubbed PCRTags, in almost all genes of each chromosome. Several types of genetic element are also removed or recoded. Retrotransposon sequences including long terminal repeats (LTRs) have been removed, as have subtelomeric repeats and all introns not previously characterized as being essential. As hotspots for chromosomal instability, tRNA gene sequences are removed from the 16 chromosomes to ultimately be complemented by a new 17th chromosome dedicated to tRNA genes.[17] Such widespread changes to the genome are likely to affect cellular processes and phenomena not necessarily captured by general phenotypic screens used to characterize synthetic chromosomes so far.[18] One such phenomenon is formation of extrachromosomal circular DNA (eccDNA).

eccDNA is formed when a section of DNA is excised from a chromosome and forms a circular DNA species in the nucleus. This can be accompanied by deletion of the chromosomal copy of the gene or not, with the exact mechanisms for this varying between eccDNAs.[19–21] Some of these mechanisms involve recombination between repeated sequences, including LTRs, and loci found on eccDNA elements often contain replicative elements in addition to coding.[22] Because replication and segregation of this DNA during cell division is not controlled by the standard chromosomal mechanisms, the copy number of the DNA can sometimes be dysregulated, leading to asymmetric inheritance.[23,24] As a result, populations of cells can display high levels of heterogeneity in eccDNA copy number.

In humans, the presence of extrachromosomal DNA (ecDNA, equivalent to eccDNA in yeast) is thought to be a driver of evolution.[25] Accumulation of ecDNA in the nucleus of cells is also emerging as an important factor in aging,[26] stimulation of immune response,[27] and particularly in cancer.[28,29] While being rare in normal cells, ecDNA is found in around half of all human cancers[30] and is seen in particularly high prevalence in glioblastomas.[31] Oncogenes encoded on ecDNA display increased dysregulated expression,[32] and ecDNA also interacts with chromosomal loci, enhancing their transcription[33] and promoting somatic rearrangements.[32,33] Increasingly, ecDNA is being recognized as a major factor in oncogenesis and tumor progression.

Some eccDNAs found in yeast form via recombination at LTRs and other sequences, and yeast eccDNAs are thought to contribute to adaptation to challenging environmental conditions.[22,34] Replacement of LTR sequences with loxPsym recombination sites in Sc2.0 chromosomes gives us a new opportunity to directly study the effects of such eccDNAs on the cell.

Here we report assembly and successful debugging of synthetic chromosome XI (*synXI*) of the Sc2.0 synthetic yeast genome. We developed and deployed a range of CRISPR-based approaches to combine sections of synthetic DNA *in vivo*, debug complex growth defects, and correct large structural variations. We also demonstrate that the loxPsym formatting of Sc2.0 genetic loci facilitates induced formation of eccDNA species. These molecules show inheritance patterns comparable with those observed in natural eccDNAs and will be a useful tool for future studies of these important molecules.

RESULTS

synXI design and synthesis

In line with the other synthetic chromosomes generated by the Sc2.0 project, we designed *synXI* following specific design criteria[16] (Table S1). These include removal of tRNA sequences, repeat DNA, non-essential introns, and transposon-associated sequences; replacement of telomeres with a custom-designed telomere seed sequence; and conversion of all TAG stop codons to TAA codons. We also introduced 457 pairs of PCRTag watermarks and inserted 199 loxPsym recombination sites into the 3′ UTR of non-essential genes and sites of key design changes.

The resulting sequence, *synXI*_3.34, consists of 18 megachunks, A–R, further subdivided into 87 chunks (Figure 1A; Table S8). The total amount of DNA synthesized as chunks was 701,706 bp, to be assembled into a 659,617-bp chromosome. Post-synthesis edits were made to the *synXI* design to correct a stop codon modification design error. To ensure that the synthesized DNA conformed to the updated chromosome version, *synXI*_3.36, we modified 2 chunks to incorporate the changes. A summary of *synXI* versions is shown in Table 1, and more in-depth descriptions of strains and chromosome versions can be found in Tables S2 and S6.

synXI assembly

synXI was assembled in two parallel construction workflows

For our initial *synXI* assembly host, we used a *MAT**a*** BY4741 strain with a *kanMX4* marker gene insertion in *YKL220C*[35] that we modified to have a *TRT2* threonine tRNA gene at the *chrXV HIS3* locus to complement the loss of the unique tRNA encoded in *chrXI* that would be removed by integration of *synXI* chunk B5 (Table 1).

Prior to transformation into the recipient yeast strain, we enzymatically assembled the constituent megachunks of *synXI* from synthesized DNA *in vitro*, as described previously.[10,16,18,36] The overall construction strategy is outlined in Figure 1A. We built

CellPress
OPEN ACCESS

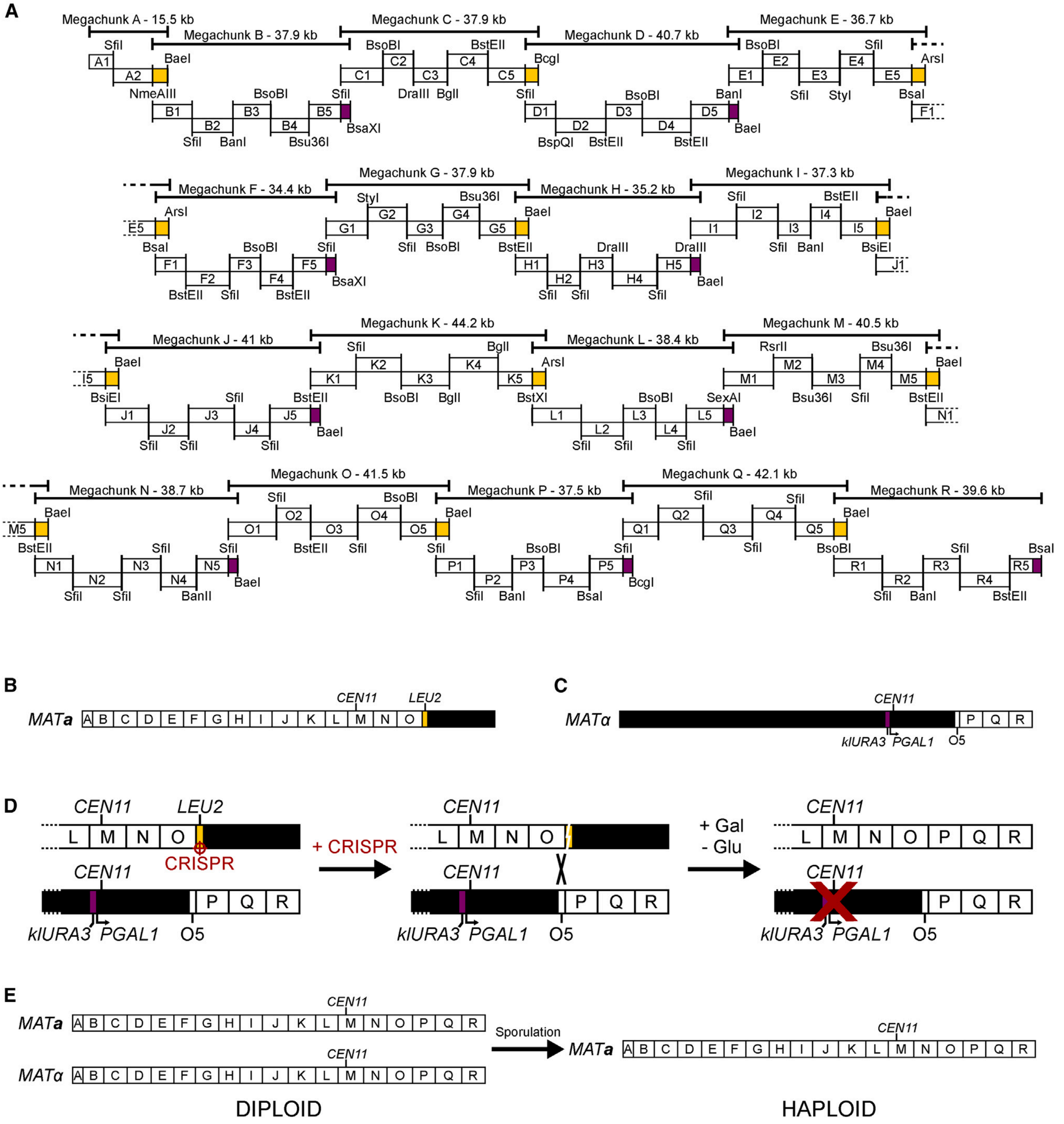

Figure 1. Synthetic chromosome XI design, synthesis, and assembly

(A) Schematic overview of the synthetic DNA sections making up *synXI*_3.34, with megachunk groupings and assembly restriction sites indicated. Purple blocks indicate a *URA3* marker gene, and yellow blocks indicate a *LEU2* marker gene.

(B) Topology of the *synXI* assembly strain ysXIa25, with white lettered boxes representing integrated megachunk sections and black sections representing wild-type sequence.

(C) Topology of the *synXI* assembly strain ysXIa30, with white lettered boxes representing integrated chunk or megachunk sections and black sections representing wild-type sequence.

(D) Overview of the method to consolidate synthetic chromosomal sequences in a diploid cell *in vivo*, generating a complete synthetic chromosome.

(E) Overview of the generation of a haploid strain containing a single copy of the complete *synXI*.

See also Figure S1.

Table 1. Summary of the strains used to construct and debug *synXI* and the iterative versions of the *synXI* chromosome

Assembly strains

Strain	*synXI* sequence assembled *in vivo*	*TRT2* location	Assembly marker	Mating type
ysXIa01	–	*HIS3*	–	a
ysXIa02	A	*HIS3*	*LEU2*	a
ysXIa03	A–B	*HIS3*	*URA3*	a
ysXIa04	A–C	*HIS3*	*LEU2*	a
ysXIa05	A–D	*HIS3*	*URA3*	a
ysXIa06	A–E	*HIS3*	*LEU2*	a
ysXIa07	A–F	*HIS3*	*URA3*	a
ysXIa08	A–G	*HIS3*	*LEU2*	a
ysXIa09	A–H	*HIS3*	*URA3*	a
ysXIa10	A–I	*HIS3*	*LEU2*	a
ysXIa11	A–J	*HIS3*	*URA3*	a
ysXIa12	A–K	*HIS3*	*LEU2*	a
ysXIa13	A–L	*HIS3*	*URA3*	a
ysXIa14	A–M, growth defect	*HIS3*	*LEU2*	a
ysXIa16	A–M (partial)	*HIS3*	*LEU2*	a
ysXIa17	A–M (partial)	*HIS3*	*URA3*	a
ysXIa18	A–M	*HIS3*	*LEU2*	a
ysXIa19	A–M	*HIS3*/*chrXI*	*LEU2*	a/α
ysXIa20	A–M	*chrXI*	*LEU2*	a/α
ysXIa21	A–M	*chrXI*/plasmid	*LEU2*	a/α
ysXIa22	A–M	plasmid	*LEU2*	a
ysXIa23	A–N	plasmid	*URA3*	a
ysXIa24	A–O (partial)	plasmid	*LEU2*	a
ysXIa25	A–O	plasmid	*LEU2*	a
ysXIa26	O5	*chrXI*	*LEU2*	α
ysXIa27	O5–P	*chrXI*	*URA3*	α
ysXIa28	O5–Q	*chrXI*	*LEU2*	α
ysXIa29	O5–R	*chrXI*	*URA3*	α
ysXIa30	O5–R	*chrXI*	–	α
ysXIa31	O5–R(*CEN11**)	*chrXI*	–	α
ysXIa32	A–O, O5–R (*CEN11**)	*chrXI*/plasmid	*LEU2*	a/α
ysXIa33	A–R, *CEN11**	*chrXI*/plasmid	–	a/α
ysXIa34	A–R, A–R	plasmid	–	a/α

synXI strains

Strain	*synXI* version	mt
ysXIb01	*synXI*_9.01	–
ysXIb02	*synXI*_9.02	–
ysXIb03	*synXI*_9.03	–
ysXIb04	*synXI*_9.04	–
ysXIb05	*synXI*_9.05	–
ysXIb06	*synXI*_9.06	–
ysXIb08	*synXI*_9.08	–
ysXIb09	*synXI*_9.09	–
ysXIb10	*synXI*_9.10	–
ysXIb11	*synXI*_9.10/*chrXI*	+
ysXIb12	*synXI*_9.10	+
ysXIb13	*synXI*_9.11	+
ysXIb14	*synXI*_9.11/*synXI*_9.11	+
ysXIb16	*synXI*_9.11	+
ysXIb17	*synXI*_9.11	+

synXI chromosome versions

Version	Description
*synXI*_3.34	*synXI* design as synthesized
*synXI*_3.36	*synXI* design with erroneous TAG codons fixed
*synXI*_3.37	*synXI* design with *CEN11* region redesigned
*synXI*_9.01	initial *synXI in vivo* assembly
*synXI*_9.02	*LEU2* insertion in I5, *kanMX4* insertion in J4
*synXI*_9.03	J repeats reduced
*synXI*_9.04	J5 *URA3* removed
*synXI*_9.05	J repeats removed
*synXI*_9.06	*URA3* insertion at P5
*synXI*_9.07	Q repeats reduced
*synXI*_9.08	Q repeats removed
*synXI*_9.09	bacterial transposon sequence removed
*synXI*_9.10	*HBS1-OMA1* locus replaced with wild type
*synXI*_9.11	*PRP16* stop TAG stop swapped to TAA

The "*synXI* sequence assembled *in vivo*" column indicates the amount of synthetic sequence successfully integrated to replace *chrXI* sequence. Letters represent whole megachunks, and O5 refers to chunk O5. *CEN11** indicates that *CEN11* has been replaced with the *klURA3-GAL1p-CEN11* construct. The presence or absence of mitochondrial function is given in the "mt" column. More details on strains used can be found in Table S2. More details on *synXI* versions can be found in Table S6.

up the synthetic chromosomal sequence *in vivo* by systematic replacement of the wild-type *chrXI* sequence using the switching auxotrophies progressively for integration (SwAP-In) methodology in this manner. We successfully performed iterative megachunk integrations to generate strain ysXIa13, containing megachunks A–L, with no fitness defects observed.

Isolation of a megachunk M integrant proved to be challenging. The delay in assembly progress prompted us to start a second assembly workflow to assemble the last 3 megachunks of *synXI* in a *MAT*α construction host strain, ysXIa26. This strain featured a modified *CEN11* that can be selectively disrupted to promote chromosomal loss during mitosis.[37] We assembled the *synXI* region from chunks O5–R5 in this strain and removed the final integrated *URA3* marker with CRISPR-Cas9.

Following successful integration of megachunk M in the *MAT**a*** host, we removed the *TRT2* gene from the *HIS3* locus and replaced it with pRS413-*chrXI*_tRNA, a vector containing an array

of the native tRNA genes of *chrXI* under the control of promoter and 3′ UTR elements from *Ashbya gossypii* and *Eremothecium coryli*.[17] This tRNA array complements the other tRNAs removed during assembly of *synXI* and is compatible with downstream consolidation of multiple synthetic chromosomes in one cell. We then successfully integrated megachunks N and O. After assembling megachunks A through O in the *MAT**a*** construction host (Figure 1B) and the region spanning chunk O5 to megachunk R in the *MAT*α construction host (Figure 1C), we mated these two strains to form a diploid with all the *synXI* sequence in one cell, albeit divided between two chromosomes.

synXI was formed by combining two semi-synthetic chromosomes in vivo

To combine the two *synXI* sections into one complete synthetic chromosome, we targeted CRISPR-Cas9 to the *LEU2* marker inserted in the 5′ end of O5 in the chromosome inherited from ysXIa25 (with megachunks A–O). This generated a double-strand break (DSB) at the 5′ end of the synthetic section of *synXI*.A-O, which could be repaired via host-mediated homologous recombination using the chromosome inherited from ysXIa31 (*synXI*.O5-R) as a repair template (Figure 1D). Cells were then grown overnight in galactose medium (YPGal) to induce *CEN11* disruption and subsequent loss of the partially synthetic copy of *chrXI* while maintaining the fully synthetic chromosome *synXI*_9.01 (Figures 1D and S1). All 6 colonies that were tested by PCRTag analysis showed no detected wild-type sequence and contained a fully synthetic chromosome XI. We sporulated the *synXI* diploid strain and dissected tetrads to isolate strain ysXIb01, a haploid strain containing *synXI*_9.01(Figure 1E).

synXI debugging

We debugged a fitness defect caused by sequence changes around the centromere

While attempting integration of megachunk M, we were unable to isolate a fast-growing transformant colony with full megachunk integration. We were able to identify one colony with a fully integrated megachunk M sequence, designated strain ysXIa14. However, this strain had a severe growth defect (i.e., a bug) on rich medium (YPD) at 30°C (Figure S2A). Replacement of the native sequence with the 40.5-kb megachunk M introduces many sequence variants that could affect strain fitness, including 9 loxPsym insertions, 3 TAG stop codon recoding events, 4 intron deletions, 1 deletion of a tRNA and associated LTR sequence, and 45 recoded sections in CDSs that introduce PCRTags (Figure 2A). We assumed that one such variant was responsible for this bug; to identify the bug-associated locus, we reintroduced the five "M" chunks individually into strain ysXIa13 by targeting the corresponding chromosomal loci with CRISPR-Cas9 and providing the synthetic chunk DNA as the repair template (Figure 2A). Transformants from CRISPR-Cas9 reactions introducing chunk M2 grew visibly more slowly than those from reactions introducing the other chunks, leading us to focus on the M2 sequence for causes of the bug.

Close inspection of chunk M2 revealed an unannotated design error in the sequence proximal to the centromere, *CEN11*. Rather than inserting a loxPsym site 100 bp downstream of *CEN11*, as intended, we had deleted 34 bp of native sequence and replaced it with a loxPsym site situated 66 bp downstream of the centromere. Although the deleted bases fell outside of the annotated centromeric sequence, we decided that this anomaly in the design warranted further investigation.

To determine the effect of the synthetic *CEN11* region on fitness, we used CRISPR-Cas9 to replace the centromeric region of strain BY4742-*CEN11** with a 2.1-kb section of chunk M2, spanning the centromere and the surrounding sequence. The resulting strain, y*CEN11*d1, displayed a clear growth defect in YPD compared with BY4742 (Figure 2B). We then redesigned the centromeric region of chunk M2 to restore the inadvertently deleted sequence downstream of *CEN11* and move the loxPsym site into the 3′ UTR of the next gene, *VPS1* (Figure S2B). Strain y*CEN11*d2, in which the centromeric region of BY4742-*CEN11** has been replaced with the redesigned centromeric sequence (*CEN11*_3_37), does not display a growth defect when grown in YPD (Figure 2B).

To investigate whether other permutations of the centromeric locus might affect fitness, we generated 3 further designs. The topologies of these *CEN11* variants are shown in Figure S2B. Because the growth defect associated with the synthetic *CEN11* locus was particularly pronounced at 37°C, we assayed the *CEN11* variant strains for growth at this temperature. None of the strains, except *CEN11*_3_35, showed a noticeable effect on growth (Figure S2C). We concluded that the function of the centromere was impaired by insertion of the loxPsym site too close to the right of the centromere. We thus moved forward with chromosome design version *synXI*_3.37, incorporating redesigned *CEN11* region *CEN11*_3_37 in a new version of chunk M2, which could now integrate without conferring a fitness defect in YPD. This new version of megachunk M was successfully integrated and is the version present in the full *synXI* assembly.

Extensive repeated sequences and a transposon sequence were removed from synXI

Whole-genome sequencing of ysXIb01 and analysis using the perfect match genomic landscape strategy[38] revealed that we had constructed *synXI* but with several deviations from the designed sequence. A full list of these deviations is given in Table S5. The most notable of these was a marked increase in coverage depth at regions corresponding to chunks J1–J4 and chunks Q1–Q2 (Figure S3A). This indicated that these sequences are repeated several times on the chromosome. Indeed, the size of *synXI*_9.01 in ysXIb01 did visualize as around 200 kb larger than expected when analyzed for size by pulsed-field gel electrophoresis (PFGE; Figure S3B).

We used a CRISPR-Cas9 strategy to replace the repeated megachunk J sequence with a single copy that we assembled *in vitro* and provided as repair template (Figure 2C). Using PFGE, we then analyzed the chromosome size of 3 transformants, of which colony C had a chromosome band most consistent with significant *synXI* repeat reduction (Figure S3C). We designated this strain as ysXIb03. To confirm the extent of repeat DNA sequence removal at the J locus, we performed nanopore sequencing with ysXIb03 genomic DNA. This showed that some repeat sequence was still present, but read lengths were now long enough to capture the entire region, showing just 3 copies of chunk J1 inserted in tandem. These reads also revealed that the J1 repeats were interspersed with plasmid

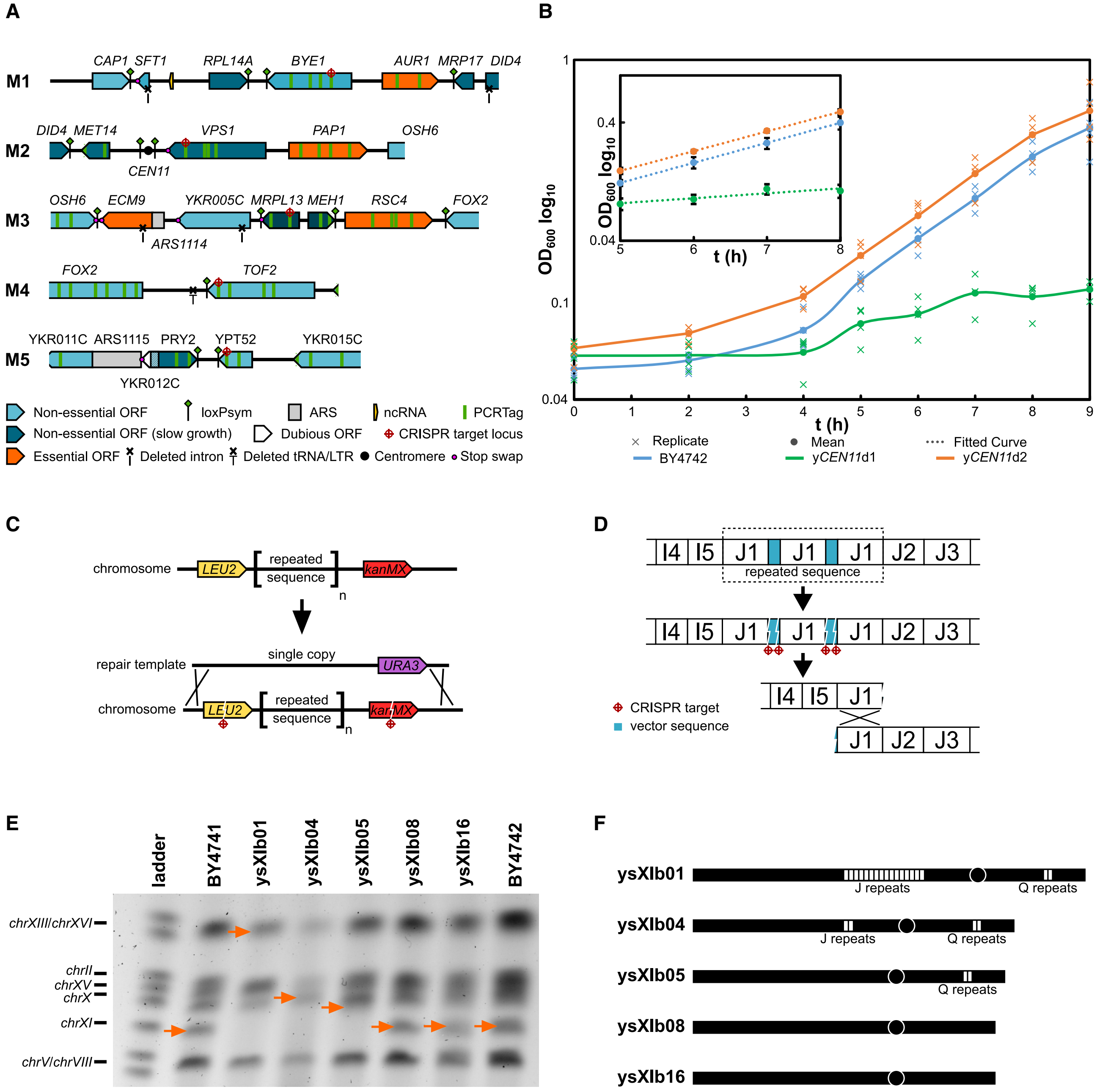

Figure 2. Debugging of centromere and repeated sequence regions in *synXI*

(A) Overview of the synthetic chromosomal locus corresponding to megachunk M, subdivided into constituent chunks. Changes to the sequence made during synthetic redesign are highlighted with symbols, explained beneath the overview.

(B) Growth of BY4742 and strains containing centromeric locus variants. Biological replicates are plotted as crosses, n = 3, with the mean value plotted as a solid line. Inset: a selection of the same data taken from a period when all cultures were undergoing exponential growth, with mean values plotted, error bars representing standard deviation, and a fitted logarithmic curve as a dotted line (BY4742 growth rate [μ] = 0.390 h^{-1}, R^2 = 1; yCEN11d1 μ = 0.096 h^{-1}, R^2 = 0.790; yCEN11d2 μ = 0.384 h^{-1}, R^2 = 1).

(C) Overview of the initial strategy to condense repeats in the megachunk J region.

(D) Structure of megachunk J repeat sequence as deduced from nanopore sequencing data and the revised strategy to condense these repeats *in vivo* by CRISPR-mediated recombination.

(E) PFGE gel of genomic DNA extracted from BY4741 and various strains generated during the repeat condensation process. Orange arrows show the inferred position of *chrXI* or *synXI*.

(F) Diagrammatic overview of the repeat sequences in the strains analyzed by PFGE in (E). Each white box represents a predicted copy of repeated sequence.

See also Figures S2 and –S4.

backbone sequence from the J1 chunk vector, a feature likely to have been missed when aligning short reads to a scaffold sequence. To remove the remaining repeated sequence at the megachunk J locus by CRISPR-directed gap repair, we transformed ysXIb03 with Cas9 and gRNAs targeting the J1 plasmid sequences flanking the J1 chunk DNA (Figure 2D). PCR screening of transformant cells to confirm loss of the plasmid-derived sequence led us to isolate strain ysXIb04, in which megachunk J repeats had been condensed down to a single copy. Because of its smaller size, we could take a more straightforward approach to repeat sequence condensation in the megachunk Q region (Figure S3D). We used CRISPR-Cas9 to replace the repeated locus with a single copy and confirmed the reduction of the repeats by PFGE (Figures 2E and 2F).

Nanopore sequencing of the resulting strain, ysIXb08, confirmed that repeated sequences in regions J and Q had been successfully removed. Interestingly, when analyzing the long sequencing reads, we also noticed that there was a sequence discrepancy in the *TRK2* CDS that we had missed by short-read sequencing analysis. The CDS showed a partial duplication and insertion of 2 foreign sequences (Figure S4). One of these sequences had partial sequence identity to the bacterial vector on which the corresponding synthetic DNA chunk was propagated, and the other was 99% identical to a gene encoding an *E. coli* transposase DDE domain protein (GenBank: QFU33765.1). We assume that the O3 chunk vector caused a fitness defect in the *E. coli* host, and a transposon insertion into the vector was selected for during pre-assembly plasmid propagation. We removed the sequence using CRISPR-Cas9 to generate strain ysXIb09, in which the *TRK2*-transposon locus had been replaced by a single intact copy of *TRK2*.

Insertion of loxPsym sites downstream of genes with mitochondrial function led to a respiratory growth bug

Routine spot assays performed after every round of integration during *synXI* construction revealed a persistent growth defect at 37°C on glycerol growth medium (YPG) following megachunk Q integration (Figures 3A and S5A). Unlike previous defects observed during chromosome assembly, this defect was not rescued by the subsequent round of megachunk integration and so not caused by marker gene insertion. Because glycerol is a non-fermentable carbon source, this defect is indicative of a problem with mitochondrial function.

Standard debugging approaches revert synthetic DNA regions back to the wild-type sequence, but doing this in ysXIb09 failed to yield any strains with rescued growth on YPG. This indicated that the defects might be in genes encoding mitochondrial proteins and that these defects could cause permanent damage to, or loss of, the mitochondrial DNA. We also found that, in contrast to BY4741, ysXIb01 colony growth was not affected by treatment with ethidium bromide, and we were unable to obtain any amplification product from PCR screens using mitochondrial genome-specific screening primers using ysXIb01 genomic DNA as a template (Figures S5B and S5C). These observations all pointed toward an absence of the mitochondrial genome, meaning that even if the underlying genetic cause of the defect was fixed, full respiratory capacity would remain absent because functional mitochondria could not be restored.

To bypass these difficulties in debugging, we attempted to use CRISPR-Cas9 to facilitate integration of as much of the megachunk Q sequence as possible into strain ysXIa27 (O5-O integrant) without introducing the defect. We co-transformed megachunk Q DNA with CRISPR-Cas9 constructs targeting DSBs to three points in the chromosomal sequence. This yielded 5 colonies, all of which had almost complete integration of megachunk Q, apart from at a locus between PCRTags YKR084C.1 and YKR087C.1. For each of these colonies, we compared PCRTag composition with the YPG 37°C phenotype (Figure 3B). Colony 2 had no associated YPG 37°C defect and had a fully synthetic megachunk Q region, except between PCRTags YKR084C.1 and YKR086W.3. We confirmed with further PCR screens that the loxPsym site introduced into the 3′ UTR of *OMA1* was also absent in this strain.

The synthetic reformatting of the identified region in megachunk Q notably contains loxPsym insertions into the 3′ UTRs of two genes encoding mitochondrial proteins, *MRPL20* and *OMA1* (Figure 3C). Deletion of *MRPL20* has been shown previously to result in mitochondrial genome loss[39,40] whereas *OMA1* is involved in maintaining respiratory supercomplexes, with null mutants showing deterioration of respiratory function.[41] These phenotypes are consistent with the fitness defect observed in the *synXI* strains. We hypothesized that the loxPsym insertions may interfere with 3′ UTR encoded targeting of mRNA to the mitochondria.[4]

To fix the respiratory growth defect, we used CRISPR-Cas9 to replace the synthetic region in ysXIb09, that spans from *HBS1* to *OMA1*, with a PCR amplicon of the equivalent region from colony 2 of the debugging process (Figure 3C). The resulting strain, ysXIb10, still had the YPG 37°C growth defect (Figures 3D–3F) because of the prior mitochondrial damage. To replenish this strain with healthy mitochondria, we backcrossed it with BY4742-*CEN11** and then enriched for *synXI*_9.10 by galactose-induced loss of *chrXI* before sporulating and isolating the *MATa* strain ysXIb12. This strain, with *HBS1*-*OMA1* locus replacement and mitochondrial replenishment, showed reversion to the parental respiratory growth phenotype (Figures 3D–3F). Because replacement of the locus had reverted the *PRP16* stop codon back to TAG, we converted this back to TAA by CRISPR-Cas9. We then backcrossed the strain with BY4742-*CEN11** and isolated strain ysXIb16, which displayed good respiratory fitness (Figures 3D–3F). This strain underwent PCRTag analysis targeted to loci across *synXI* (Figures S6A and S6B) and genome sequencing to confirm the debugged sequence of *synXI*_9.11.

Ploidy issues were identified and resolved

To ensure that there were no discrepancies in *synXI*_9.11 copy number in ysXIb16, we confirmed that full genome sequencing of ysXIb16 showed consistent levels of read coverage across all regions of the genome (Figure 4A). However, upon mating ysXIb16 with BY4742, we found that dissected spores had low viability frequencies (Figure 4B). When we tested ysXIb16 on L-canavanine plates, we failed to observe any surviving colonies, indicating that ysXIb16 is not a haploid strain (Figure 4C). Because sequencing of this strain showed no discrepancies in read coverage or heterogeneity, we concluded that ysXI16b is a homozygous diploid. We expressed a plasmid-borne *MAT*α

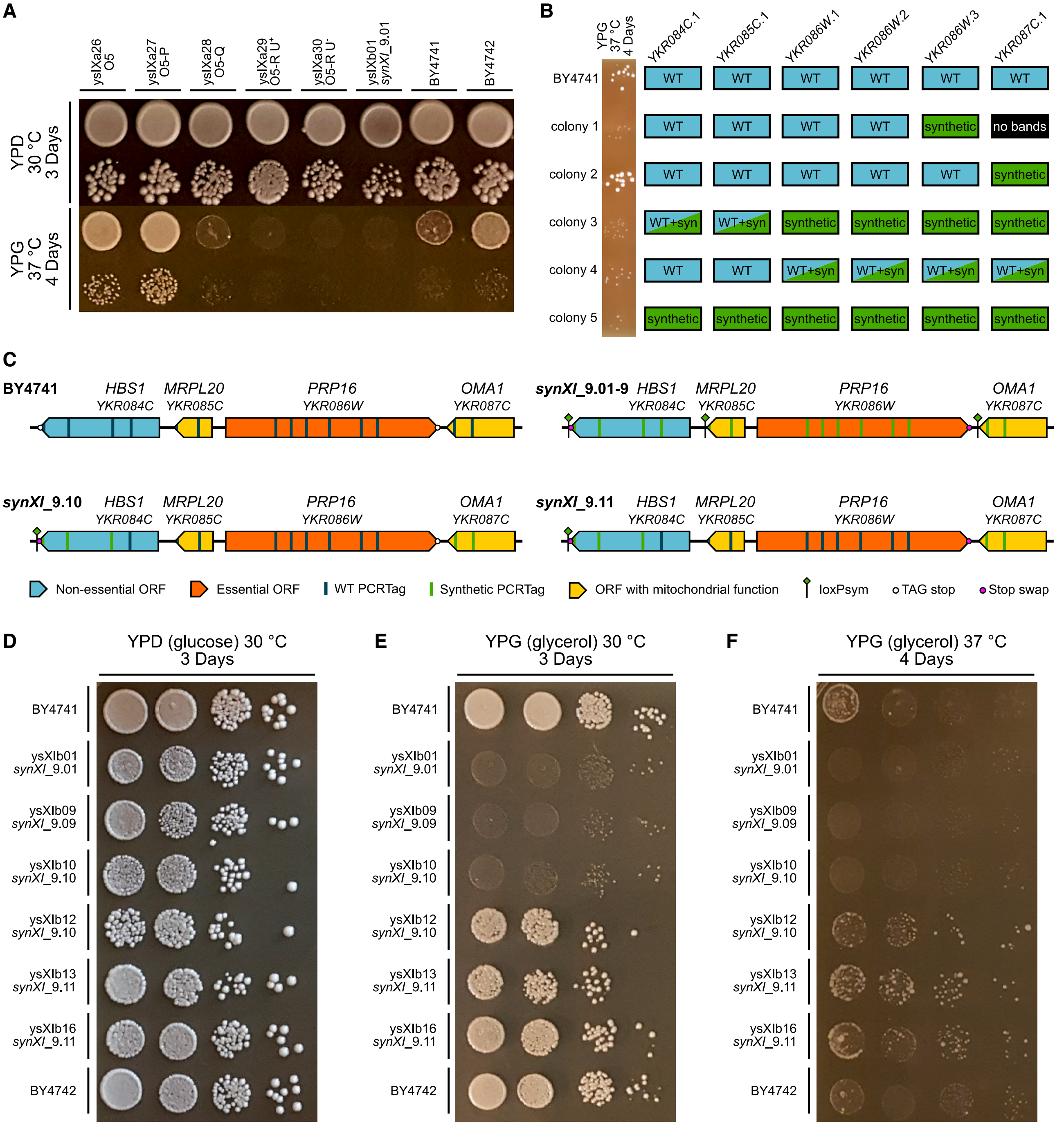

Figure 3. Successful debugging of a respiratory growth defect associated with megachunk Q

(A) Growth spot assays of *synXI* assembly intermediates following megachunk integration on YPD (glucose) and YPG (glycerol) to test respiratory function. For each strain and condition, the top spot is a $\times 10^{-1}$ dilution, and the bottom spot is a $\times 10^{-3}$ dilution.

(B) PCRTag screening results at the YKR084C.1-YKR087C.1 locus for debugging megachunk Q transformant colonies, with corresponding $\times 10^{-3}$ dilution YPG 37°C growth spots on the left, indicating respiratory function.

(C) Schematics of the *HBS1-OMA1* locus in BY4741 and the *in vivo synXI* iterations.

(D–F) Growth spot assays with strains involved in respiratory growth defect debugging on YPD (glucose) and YPG (glycerol) to test respiratory function. Dilution spots increase in steps of $\times 10^{-1}$ from $\times 10^{0}$ on the left to $\times 10^{-3}$ on the right.

See also Figure S5.

CellPress
OPEN ACCESS

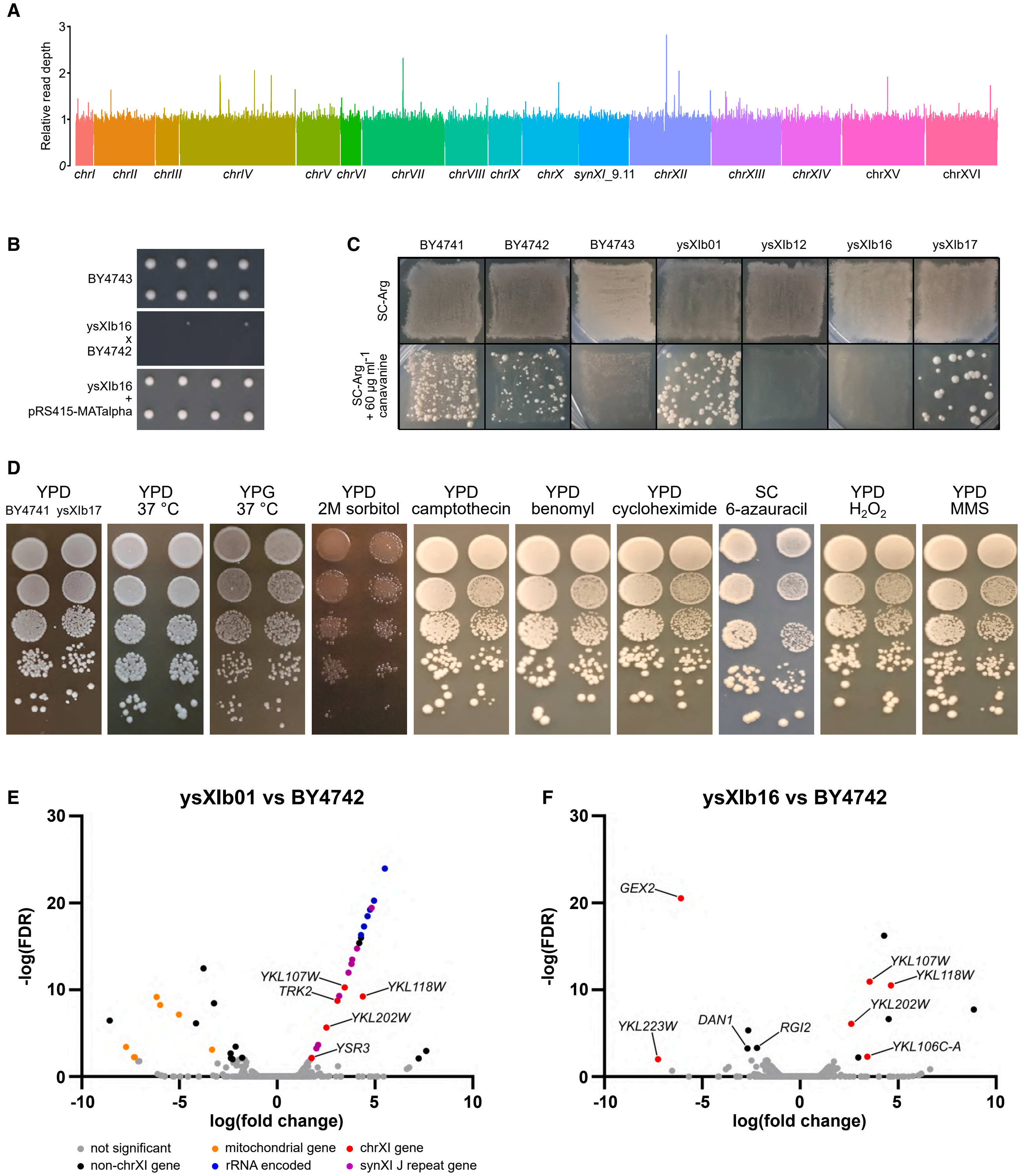

Figure 4. Assessing ploidy, fitness, and transcriptional profile of the *synXI* strain

(A) Illumina sequencing read coverage over the whole genome of ysXIb16.

(B) Spores from dissected tetrads derived from sporulated strains BY4743, ysXIb16 × BY4742, and ysXIb16 pRS415-*MAT*α. Spores from 2 tetrads were dissected for each strain, arrayed horizontally, and grown on YPD plates for 2 days at 30°C.

(C) Canavanine ploidy assay patches. Strains were grown on YPD and then replica plated onto SC-Arg with and without canavanine and grown at 30°C. Growth on canavanine is indicative of haploidy.

(legend continued on next page)

mating locus in the strain and sporulated, observing good spore viability (Figure 4B). We performed L-canavanine assays on a colony isolated from tetrad dissection, ysXIb17, which confirmed that this strain is indeed haploid. We performed further canavanine assays, which showed that the initial *synXI*_9.01 strain, ysXIb01, is haploid but ysXIb12, the strain that underwent backcrossing after editing the *HBS1-OMA1* locus, is not (Figure 4C). It is therefore likely that the homozygous diploidy was introduced during this backcrossing process.

The *synXI* strain has high phenotypic similarity to the parental strains

The synXI strain shows robust growth under a variety of conditions and perturbations

We performed growth spot assays with ysXIb17 on a wide range of medium types and conditions designed to test the robustness of various cellular processes (Figures 4D and S6C). Under all conditions tested, ysXIb17 performed well and showed no notable defects compared with the parental strain, including in YPG medium.

Altered transcription in synXI has been fixed by the debugging process

We used RNA sequencing (RNA-seq) analysis to compare the transcriptional profiles of the parental strain with the initial *synXI* assembly strain ysXIb01 and to ysXIb16, which contains the debugged *synXI*_9.11. We selected BY4742 as our parental control because its auxotrophic profile most closely matched the *synXI* strains.

The genes showing significantly different expression (false discovery rate [FDR] < 0.01) compared with BY4742 from the initial strain before debugging (ysXIb01) largely fall in a few categories (Figure 4E; Tables S9 and S10). 8 genes with higher expression are located in the repeat sequences corresponding to megachunk J. Presumably, higher transcript levels of these genes are a result of their expanded copy number. 6 genes with lower expression are encoded on the mitochondrial genome, and their differential expression is likely related to the respiratory growth defect we see in the cells. Another interesting group with higher transcription levels maps to open reading frames (ORFs) encoded within the 35s rRNA. The function of these ORFs, *YLR154W-A/B/C/E/F* and *YLR154C-G*, is not clear, but their increased transcription may indicate an increase in the copy number of the DNA encoding the 35S rRNA. Of the other differentially expressed genes, 5 are located on *synXI*. These include *TRK2*, the site of the bacterial transposon insertion.

When comparing the transcriptional profile of the final strain, ysXIb16, with BY4742 (Figure 4F), we observe that the debugging process has reverted the transcription of the megachunk J repeat genes, the mitochondrial genes, the transcripts embedded in the 35s rRNA, and *TRK2* to showing no significant differences to the parental strain. There are 6 significantly differentially expressed genes that are located on *synXI*. Of these, only the increase in *YKL107W* transcription is likely to have biological relevance. This gene encodes an aldehyde reductase involved in detoxification of toxic aldehydes.[42] An explanation for this increase in transcription is not immediately clear to us, although recoding of the CDS to incorporate PCRTags and a BstEII restriction site may be the underlying cause.

There are a further 7 genes showing significantly different expression between ysXIb16 and BY4742 that are not located on *synXI*. These consist of 5 ORFs that are of dubious or unknown function and 2 genes with lower transcription in ysXIb16: *RGI2* and *DAN1*. Neither *RGI2* or *DAN1* was differentially expressed in ysXIb01. *DAN1* and *RGI2* have been shown previously to be repressed by aerobic growth[43] and high glucose conditions,[44] respectively. Because the transcriptional changes are modest and limited to these 2 genes, slight differences in oxygen and glucose availability to the cells while culturing the strains may explain these differences.

The SC2.0 format *GAP1* locus is a promising tool for studying eccDNA behavior

Extensive characterization revealed no fitness disadvantages associated with the reformatted synXI GAP1 locus

Our characterization of strains in which *synXI*_9.11 completely replaces *chrXI* revealed very close transcriptional and phenotypic similarity to the parental strains. However, it is possible that the design principles we implemented have more subtle or situation-specific effects that we have not observed. One behavior that could be expected to be substantially affected is formation of certain species of eccDNA. The canonical example of a functional eccDNA in yeast is the *GAP1* eccDNA, thought to be formed via a recombination event between LTR sequences flanking *GAP1* and *ARS1116* in *chrXI*[22,45,46] (Figure 5A). Circularization of *GAP1* and *ARS1116* into an eccDNA allows cells to vary the copy number of *GAP1* within a population. *GAP1* encodes a general amino acid permease,[47] and *GAP1* copy number expansion through eccDNA formation is thought to be enriched for under nitrogen-limited conditions by improving a cell's ability to import amino acids under nitrogen starvation.[22,45] In the *synXI* synthetic reformatting process, the LTR (δ) regions involved in *GAP1* locus circularization, *YKRCδ11* and *YKRCδ12*, were removed and replaced with loxPsym sites (Figure 5B). To ensure that reformatting of the *GAP1* eccDNA locus does not have a negative impact on Gap1 function or cell fitness, we performed extensive characterization of strains with the synthetic *GAP1* locus (BY4741-$GAP1_{syn}$). We

(D) Growth spot assays of ysXIb17 and a BY4741 parental control under various conditions to assess cellular fitness. Cultures were serially diluted and spotted from top to bottom, with dilutions increasing from $\times 10^0$ in steps of $\times 10^{-1}$. BY4741 was spotted on the left, and ysXIb17 was spotted on the right. Sorbitol was added to 2 M, and camptothecin was added to 1 μg ml^{-1}. Other additives were added as indicated in the STAR Methods. Unless otherwise indicated, plates were incubated at 30°C.
(E) Volcano plots showing transcript abundance in ysXIb01 compared with BY4742, as determined by RNA-seq.
(F) Volcano plots of transcript abundance in ysXIb16 compared with BY4742, as determined by RNA-seq.
In (E) and (F), the y axis represents statistical significance in the form of $-\log_{10}$ of the FDR, and the x axis represents the $\log_{10}$-fold change in transcript abundance compared with BY4742 levels. Significant points are those with FDR < 0.01. See also Figure S6.

CellPress
OPEN ACCESS

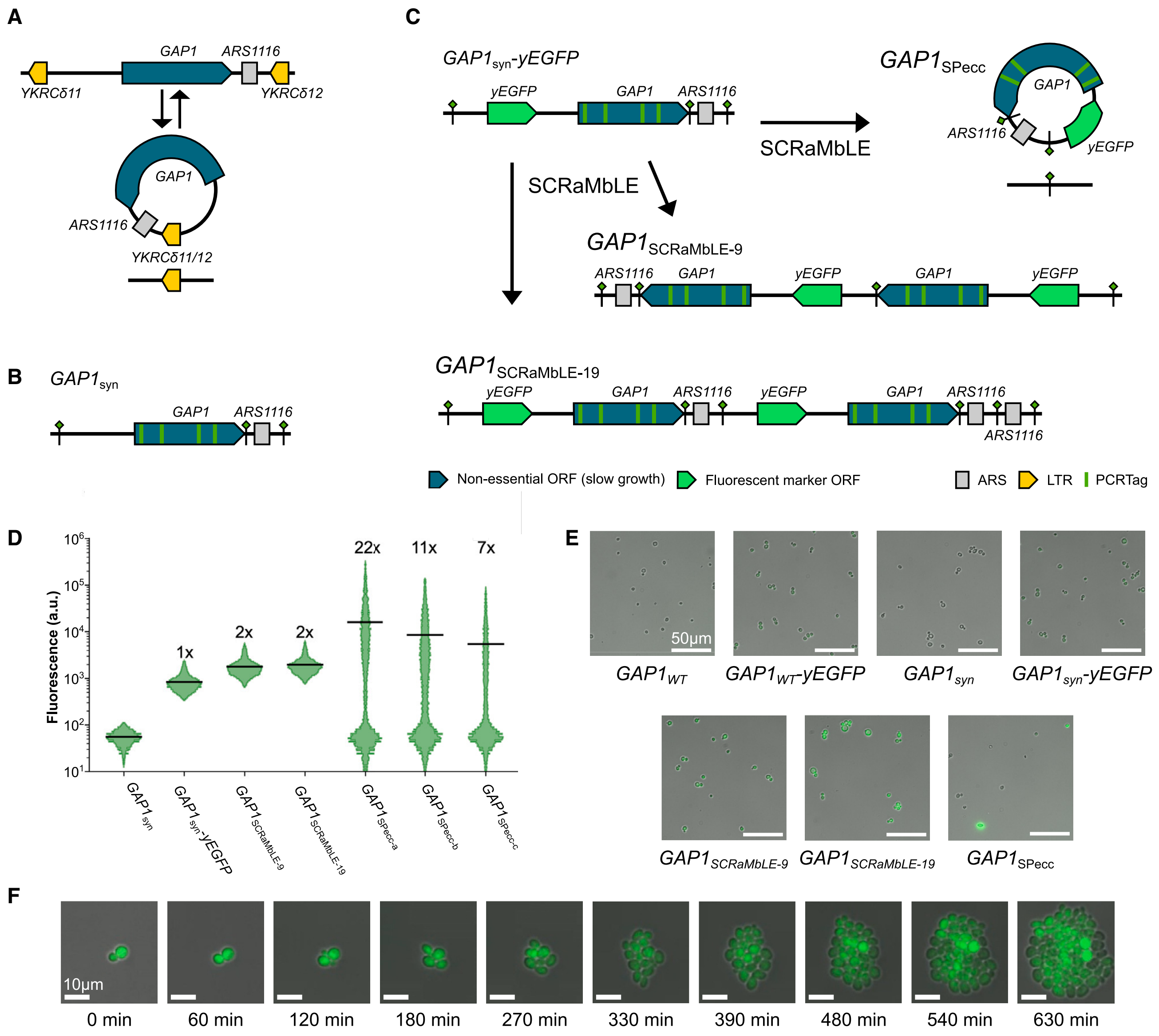

Figure 5. SCRaMbLE of the synthetic *GAP1* locus can form *GAP1* eccDNA
(A) Overview of eccDNA formation at the *GAP1* locus.
(B) Structure of the synthetic *GAP1* locus of *synXI*.
(C) Structure of the *GAP1* loci generated through SCRaMbLE.
(D) Population GFP fluorescence, as determined by flow cytometry, of strains with *GAP1* locus arrangements shown in (C). The 3 $GAP1_{SPecc}$ samples (a–c) are derived from 3 different $GAP1_{SPecc}$ colonies. Fluorescence values are arbitrary units (a.u.), horizontal black lines denote geometric means of populations, and numbers above plots give approximate relative geometric mean fluorescence compared with the $GAP1_{syn}$-*yEGFP* population.
(E) GFP fluorescence microscopy images of $GAP1_{syn}$ cells with various *GAP1* locus arrangements. Cell types are given below the images. Images were taken at 20× magnification.
(F) Time course GFP fluorescence microscopy images of $GAP1_{SPecc}$ cells taken at 20× magnification.
See also Figures S7–S10.

confirmed that the synthetic *GAP1* gene produced functional Gap1 protein and had transcription levels extremely similar to the wild-type gene, and we did not observe any detrimental effects of the $GAP1_{syn}$ locus on cell fitness (Figures S7, S8, and S10). We did, however, note that the reformatted $GAP1_{syn}$ locus has new characteristics that would make it a promising tool for generating eccDNAs through a targeted recombination mechanism.

The synthetic GAP1 locus enables inducible eccDNA formation

Isolation and study of cells with a specific rare eccDNA event can be extremely challenging. Not only are cells with the circularization

event potentially difficult to isolate, but the eccDNA is often unstable and may display asymmetric inheritance patterns, meaning that presence of the eccDNA over the course of an experiment can be difficult to maintain.[48] Fortunately, the *GAP1*$_{syn}$ locus offers a way of bypassing these issues because the elements responsible for the circularization mechanism in *GAP1* have been replaced by loxPsym sites, which can be targeted for recombination by Cre recombinase. To help study the locus, we inserted a *yEGFP* green fluorescent protein reporter gene between the *GAP1* gene and the upstream loxPsym site of BY4741-*GAP1*$_{syn}$ to generate strain BY4741-*GAP1*$_{syn}$-*yEGFP* (Figure 5C). We then introduced the SCRaMbLE plasmid pSCW11-*cre*-EBD to BY4741-*GAP1*$_{syn}$-*yEGFP*, induced SCRaMbLE with β-estradiol, and cured the strains of the plasmid. Because Cre was no longer present in the cells, and loxPsym sites are not large enough to be efficiently targeted by the native homologous recombination machinery,[49] the SCRaMbLE recombination events are far less likely to be reversible than eccDNA formation through LTRs.[46] Using this process, we were able to isolate two strains, *GAP1*$_{SCRaMbLE-9}$ and *GAP1*$_{SCRaMbLE-19}$, which had expanded the chromosomal *GAP1* locus, and a third strain, *GAP1*$_{SPecc}$, containing a *GAP1* SCRaMbLE-produced extrachromosomal circle (SPecc; Figure 5C). Extensive PCR characterization of these strains confirmed expansion events and circularization of the *GAP1* locus (Figure S9).

We used flow cytometry to determine that yEGFP levels were increased in both strains with an expanded chromosomal *GAP1* locus (Figure 5D). In contrast, the fluorescence profile of *GAP1*$_{SPecc}$ cultures showed that many cells lost *yEGFP* expression and, thus, likely lost the *GAP1* SPecc. However, many highly fluorescent cells were still present in the *GAP1*$_{SPecc}$ population and showed a spread of fluorescence with a geometric mean around 13 times higher than in the single-copy *yEGFP* parental strain. This is fully consistent with a highly variable gene copy number that would be expected from an asymmetrically inherited eccDNA (Figure 5D). We analyzed these strains by fluorescence microscopy, and the images supported the flow cytometry findings, particularly showing the variable fluorescence seen in *GAP1*$_{SPecc}$ cells (Figure 5E). Time-lapse microscopy showing a single budding *GAP1*$_{SPecc}$ cell growing into a population of cells illustrates the uneven inheritance and variable copy number of the *GAP1*$_{SPecc}$ in the population (Figure 5F). This behavior is consistent with observations of eccDNA behavior in a population following its formation.[24,48]

The synthetic GAP1 locus can be repurposed to form eccDNA containing an alternative gene of interest

Having shown that SCRaMbLE-based methods can be used to generate strains with *GAP1* SPeccs, we explored whether we could repurpose the synthetic locus to generate SPeccs containing an alternative gene of interest by replacing the *GAP1*$_{syn}$ gene of BY4741-*GAP1*$_{syn}$-*yEGFP* with the histidine biosynthesis pathway gene *HIS3* (Figure 6A). Because the parental BY4741 gene has a deletion of the native *HIS3* gene and is a histidine auxotroph, the newly generated strain BY4741-*HIS3*-*yEGFP* relies on the *HIS3* gene in the synthetic *GAP1* locus to grow in the absence of histidine. We induced SCRaMbLE in this strain and picked downstream colonies we visually assessed under blue light to have high yEGFP levels (Figures 6B and 6C). Of 4 colonies picked, all 4 were determined to contain SPeccs by PCR screen (Figure S11). To investigate retention rates of the *HIS3* SPecc with and without a selective pressure, we grew a *HIS3*$_{SPecc}$ strain in synthetic complete (SC) medium with and without supplemented histidine (Figures 6D and S12). In SC −His, the *HIS3*$_{SPecc}$ strain showed a slower growth rate than the strain with a chromosomal copy of *HIS3* but was able to reach a similar final density (Figure 6D). We observed that, when grown in SC −His, the yEGFP fluorescence of the culture with *HIS3*$_{SPecc}$ increased rapidly during exponential growth and then dropped when the culture had reached saturation (Figure 6E). When histidine was present in the medium, yEGFP fluorescence was markedly lower. This indicated that the *HIS3* SPeccs were enriched for during growth in SC −His and then lost, likely because of a modest cellular burden of the eccDNA, as reflected by its effect on growth. We analyzed cultures from the growth assay endpoint by flow cytometry and estimated that, in histidine-supplemented medium, 20.8% of cells retained the *HIS3* Specc, while in medium lacking histidine, this figure was 58.2% (Figures 6F and S13).

To investigate the dynamics of *HIS3* eccDNA levels, we subcultured the *HIS3*$_{SPecc}$ cells grown with supplemented histidine into histidine dropout medium and subcultured *HIS3*$_{SPecc}$ cells grown without histidine into histidine-supplemented medium. By flow cytometry analysis, we estimate that the population that had moved from histidine-supplemented medium to histidine dropout medium had *HIS3* SPeccs present in 66.9% of cells, while cells moving from histidine-depleted to histidine-supplemented medium saw a reduction in cells with SPeccs, down to 26.7% (Figure 6F). In this case, we were able to observe the prevalence of *HIS3* SPeccs in the population dynamically shifting in response to availability of histidine.

DISCUSSION

We assembled and debugged synthetic chromosome XI of the Sc2.0 synthetic yeast genome. We generated a yeast strain with full replacement of its native chromosome XI with a synthetic counterpart and found its fitness and transcriptional profiles to be very similar to those of the parental strain.

Complications and difficulties in the assembly process led us to develop a range of effective CRISPR-Cas9 approaches to debugging and editing the designed synthetic DNA *in vivo*. Targeted integration of synthetic DNA chunks, followed by phenotypic screening, allowed us to effectively narrow down the potential sequence causing fitness defects. By focusing on narrower regions of synthetic DNA sequence, we were able to identify underlying causes and precisely edit the problematic sequences to restore fitness. Through the process of debugging by CRISPR-Cas9-mediated integration of megachunk Q, we also had a strong indication that the efficiency of megachunk integration and subsequent colony screening could be markedly improved. Co-transformation of CRISPR-Cas9 constructs, targeting loci across the chromosomal region being replaced, reduced the number of transformant colonies while enriching for a high proportion of successfully integrated sequence. Combined with our CRISPR-Cas9 approach to combine chromosome sections *in vivo* via targeted mitotic crossover, we believe that our refined methodologies will allow much faster and more

CellPress
OPEN ACCESS

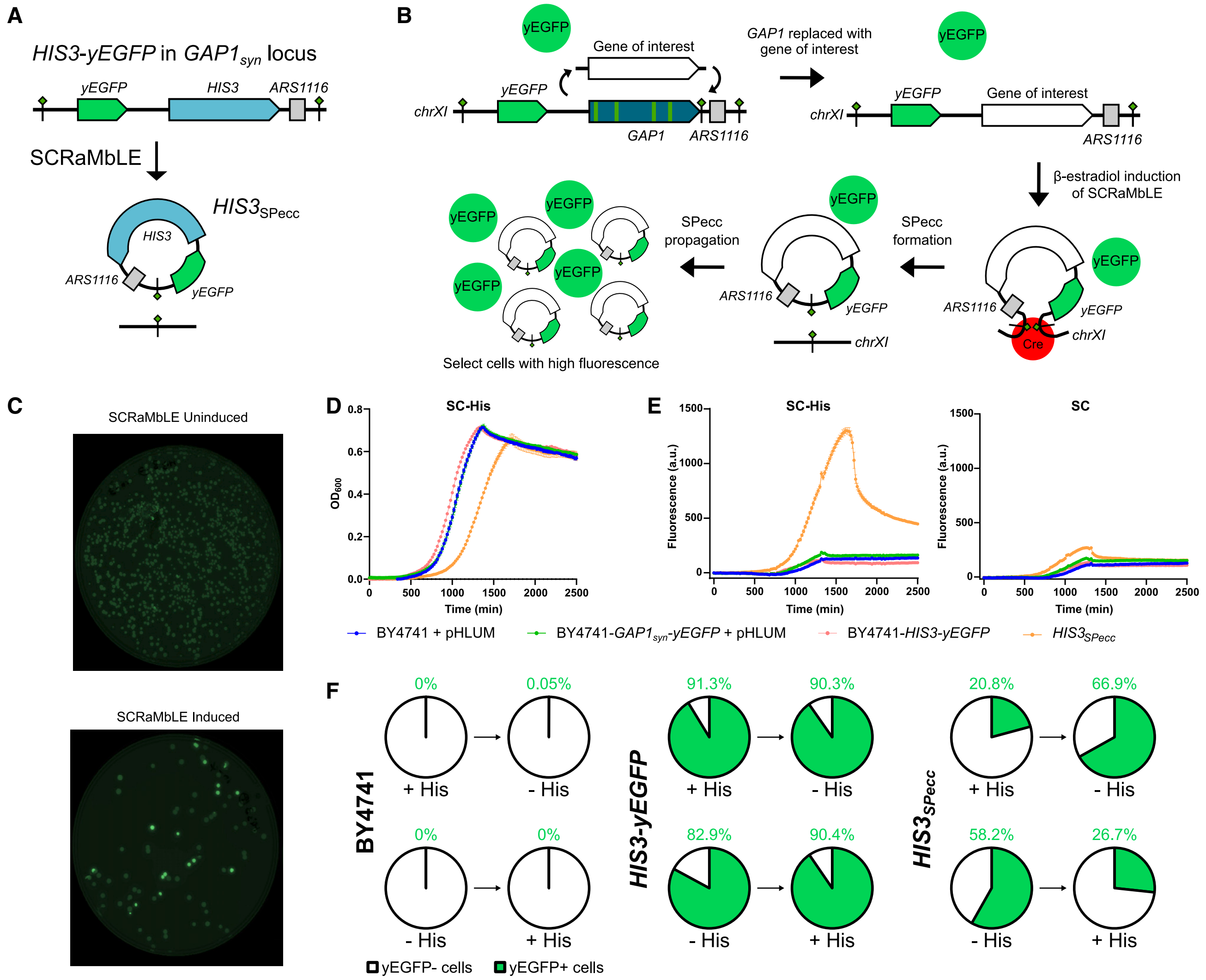

Figure 6. The synthetic *GAP1* locus was repurposed to study *HIS3* eccDNA
(A) Structure of the *HIS3-yEGFP* locus replacing the $GAP1_{syn}$ locus.
(B) Schematic of SPecc isolation using increased GFP production in SPecc cells for screening.
(C) Photographs of BY4741-*HIS3-yEGFP* pSCW11-*cre*-EBD-derived colonies on agar plates under blue light to visualize yEGFP fluorescence. The top photo shows a plate inoculated from an uninduced culture, and the bottom photo shows a plate inoculated from a culture in which SCRaMbLE was induced.
(D) Growth of BY4741-$HIS3_{SPecc}$ and parental strains in defined medium without histidine (SC −His) at 30°C. Mean optical density 600 (OD_{600}) values from 3 biological replicates are plotted as circles; error bars represent standard deviation.
(E) Fluorescence values of BY4741-$HIS3_{SPecc}$ and parental strain cultures in defined medium with (SC) and without histidine at 30°C. Medium-blanked mean fluorescence values from 3 biological replicates are plotted for each culture; error bars represent standard deviation.
(F) Charts showing the proportion of cells in a population determined to be expressing (yEGFP+ cells) and not expressing (yEGFP− cells) a yEGFP reporter, as determined by flow cytometry. For each strain, as listed to the left of charts, cells were grown overnight at 30°C in one medium type, sub-cultured into another medium type, and grown overnight at 30°C. Flow cytometry was performed on cultures after each stage of overnight growth. For each pair of charts, the left chart represents the first overnight culture, and the right chart represents the second overnight culture sub-cultured from cells in the left chart. "−His" denotes cultures grown overnight in SC −His, and "+His" denoted cultures grown overnight in SC. Green figures above charts indicate the percentage of cells within the population assessed to be producing yEGFP.
See also Figures S11–S13.

efficient parallelized assembly of future synthetic chromosomes and genomes.

Over the course of assembling and debugging *synXI*, we uncovered cases of redesigned sequences in non-coding DNA leading to phenotypic defects in the host cell. We identified insertion of loxPsym sites to be a common underlying cause of these fitness defects. Insertion of a loxPsym site 66 bp downstream of the annotated centromere *CEN11* caused a pronounced slowing of growth. A possible explanation could be that the sequence change interferes with the process of Cse4

binding to the DNA to promote kinetochore assembly.[50,51] Although various permutations made to the sequence around *CEN11* did not produce a similar effect, sequence surrounding annotated centromeres should be edited with caution in future projects. We also found that loxPsym insertions into the 3′ UTRs of *MRPL20* and *OMA1*, both encoding mitochondrial proteins, led to strains defective in mitochondrial function. Additionally, we were unable to isolate a strain with successful integration of a loxPsym site into *MRS4*, another gene encoding mitochondrial function.[52] Previous studies have shown that, in genes encoding proteins with mitochondrial function, the 3′ UTR is important in localizing mRNA to the mitochondrial outer membrane.[4] This occurs through mechanisms that are either dependent or independent of binding to the protein Puf3p via a consensus binding domain within the 3′ UTR.[53] Previous studies have identified Puf3p binding consensus sequences in the 3′ UTRs of *MRPL20* and *MRS4*.[54,55] We speculate that the designed insertion of loxPsym sites into a small subset of these mitochondrial genes interferes with correct localization of mRNA to the mitochondria, resulting in defects in respiratory growth. Strains in which these loxPsym sites were removed reverted to a healthy respiratory growth phenotype following mitochondrial DNA restoration. A similar effect was observed during construction and debugging of *synXIV*.[56] In future synthetic construct design, we would recommend caution when altering the 3′ UTR sequences of genes with mitochondrially targeted mRNA or any other regulatory motifs in the region.

As well as fitness defects, another unintended feature of the *synXI* construction process was introduction of structural variations in the form of large DNA sequence repeats, as seen in other synthetic yeast chromosome studies.[57–59] Some of these repeats are artifacts of the construction process. In the case of megachunk J, an inefficiently cut SfiI site in the J1 chunk plasmid appears to be directly associated with generation of the repeats. It is also notable that the repeats in the megachunk Q region contain the majority of the *MRPL20* CDS. Because the respiratory growth defect was fixed after we reverted *MRPL20* to its native sequence, it is possible that the repeats in megachunk Q subtly alleviated the effects of a defective synthetic *MRPL20* and were thus selected for.

Short-read sequencing was able to determine that repeats were present, via increased read coverage depth over repeated sequence, but long-read sequencing was much better at capturing the structure of repeated sequences and helped us identify the best strategy for their condensation. Because long-read sequencing data allowed us to construct contigs *de novo*, without a predicted sequence scaffold, it was also better at identifying unexpected insertions, like the bacterial transposase sequence discovered in *TRK2*. We conclude that use of long-read sequencing to help determine the structure of chromosome-scale synthetic constructs is highly beneficial. Where extensive repeats occur, the CRISPR-Cas9 repeat condensation methods we developed here are effective at restructuring a chromosome to remove higher-order deviations from the designed sequence.

Removal of native repeat sequences in the Sc2.0 chromosome redesign process is intended to improve stability and reduce the incidence of genomic rearrangements. However, in some cases, dynamic events involving interaction between repeated elements have biological function, such as in some eccDNA formation events. *GAP1* eccDNA formation to adapt to nitrogen-limited conditions is often cited as an example of eccDNA function in the literature.[22,45,46,48] Its location on *chrXI* gave us a good test case to investigate the unintended effects on eccDNA function that could be caused by the synthetic chromosome redesign. The native LTRs that have been shown previously to recombine to form the *GAP1* eccDNA in wild-type strains have been replaced by loxPsym sites in *synXI*, effectively removing the proposed natural circularization mechanism. Our testing found no negative fitness impact associated with the synthetic *GAP1* locus under common lab conditions.

The synthetic reformatting of the *GAP1* locus gave us an opportunity to directly study *GAP1* eccDNA by using a SCRaMbLE-analogous process to induce irreversible *GAP1* eccDNA formation in cells with a synthetic *GAP1* locus. While previous work has emulated an eccDNA by using a recombinase to remove replicative elements from a centromeric plasmid,[26,60] the SPecc method directly causes eccDNA formation from a chromosomal locus. We were able to show that these SPeccs were inherited asymmetrically down cell lineages and that they resulted in widely heterogeneous expression levels within a population. This behavior follows our expectations of the native *GAP1* eccDNA species.[24,48]

While the synthetic *GAP1* locus was designed and generated as part of the *synXI* construction process, synthetic loci allowing generation of SPeccs analogous to other eccDNAs or containing particular genes of interest do not require full synthetic chromosomes to be exploited. We showed that the SPecc methodology could be used to generate eccDNAs of the *HIS3* gene. Not only did the SPeccs give us the ability to induce specific eccDNA formation from a chromosomal locus, but we were also able to use fluorescent reporters to monitor the propagation dynamics of circular DNA in a population and correlate this with cellular behaviors such as growth.

We believe the BY4741-*GAP1*$_{syn}$-*yEGFP* strain to be a promising tool for future study of eccDNA dynamics. We showed that the *GAP1*$_{syn}$-*yEGFP* locus can be used as a chromosomal landing pad for eccDNA formation, where the *GAP1* gene can be replaced with an alternative gene of interest. We have the ability to select for *GAP1* using L-citrulline and counter-select against *GAP1* using D-histidine,[61] and locus formatting enables inducible formation of SPeccs containing an autonomously replicating sequence (ARS), a fluorescent marker gene, and the gene of interest. Cells with the SPeccs are easily selected for, and dynamics of SPecc propagation in a population can be assessed using the fluorescent reporter.

Given the emerging importance of eccDNAs in the fields of evolution, immunology,[27] cancer,[25,62] and aging,[26] we believe that the SPecc methodology will be a valuable tool for a wide range of future studies in yeast and other organisms. SPeccs may also be promising tools for fields such as synthetic biology and biotechnology. Spontaneous formation of eccDNA has been found to be a mechanism for improvement in xylose fermentation during evolutionary engineering experiments.[63] SPecc formation may be a route to accelerating strain improvement strategies. Additionally, the ability to confer a population with widely

heterogeneous expression of a foreign gene or pathway of interest could be used to determine optimal gene expression levels or generate strains with self-sacrificing individuals; for example, where a minority sequester toxic metals or metabolites for the benefit of the wider population.

The Sc2.0 synthetic chromosome XI has been assembled and will make up part of the complete synthetic yeast genome. Many of the lessons learnt in this project, as well as our approaches to chromosome design, assembly, debugging, and structural manipulation of the genome, will contribute to future synthetic genome projects in yeast and in an expanded range of organisms. Additionally, our study and manipulation of a synthetic eccDNA locus has delivered a new tool enabling us to generate eccDNAs and examine their functions and behavior *in vivo*. This will open up new methodologies for synthetic biology and biotechnology and provide new tools for those studying the roles played by these DNA species in many aspects of biology.

Limitations of the study

While we worked hard to ensure that the completed *synXI* strain had fitness equivalent to its wild-type parent under a wide range of conditions, our study does not cover all eventualities, and so sequence changes that lead to fitness defects for non-tested conditions may still be present in the design that could be found at a later date. For the sequence changes that led to fitness defects identified here, it is important to note that we did not fully determine the mechanisms at play and that caution should be taken to extrapolate from the handful of fitness defect cases we see here when changing only one chromosome in the yeast genome. Similarly, when testing for generation of eccDNA species from our synthetic loci, it's important to recognize that our *cre-loxPsym*-based method can generate other types of copy number variations in the yeast genome, but our fluorescent protein-based screening method preferentially selects for SPeccs because of their much higher copy number. Further study of strains containing SPeccs will be needed to understand their role in strain fitness and their inheritance.

CONSORTIA

This work is part of the international Synthetic Yeast Genome (Sc2.0) consortium. The chromosome design and building consortium includes research groups worldwide: Boeke Lab at Johns Hopkins University and New York University (led chromosomes I, III, IV, VI, VIII, and IX); Chandrasegaran lab at Johns Hopkins (led chromosomes III and IX); Cai Lab at the University of Edinburgh and University of Manchester (led chromosomes II and VII and tRNA neochromosome); Yue Shen's team at BGI-Research SHENZHEN (led chromosomes II, VII, and XIII); Y.J. Yuan's team at Tianjin University (led chromosomes V and X); Dai Lab at Tsinghua University and Shenzhen Institute of Advanced Technology, CAS (led chromosome XII); Ellis Lab at Imperial College London (led chromosome XI); Sakkie Pretorius's team at Macquarie University (led chromosomes XIV and XVI); Matthew Wook Chang's team at National University of Singapore (led chromosome XV); Bader and Boeke Labs at Johns Hopkins University (led design and workflow); and Build-A-Genome undergraduate teams at Johns Hopkins University and Loyola University Maryland (contributed to chromosomes I, III, IV, VIII, and IX). The Sc2.0 consortium includes numerous other participants and are acknowledged on the project web site (www.syntheticyeast.org).

STAR★METHODS

Detailed methods are provided in the online version of this paper and include the following:

- KEY RESOURCES TABLE
- RESOURCE AVAILABILITY
  - Lead contact
  - Materials availability
  - Data and code availability
- EXPERIMENTAL MODEL AND STUDY PARTICIPANT DETAILS
  - Strains
  - Growth and media conditions
- METHOD DETAILS
  - Plasmid construction
  - DNA extraction
  - DNA transformations
  - synXI design and synthesis
  - synXI assembly
  - Switching TRT2 complementation to tRNA array
  - CRISPR/Cas9 genome editing and debugging
  - Repeat sequence condensation
  - Removal of insertion sequence in TRK2
  - Reinstating the *PRP16* TAA stop codon
  - Mating, sporulation and tetrad isolation
  - Ploidy determination and fixing
  - PCRTag analysis
  - Genome sequencing
  - Pulsed-field gel electrophoresis
  - Transcript analysis
  - Growth spot assays
  - Growth and fluorescence curves
  - Mitochondria assays
  - Flow cytometry
  - Fluorescence microscopy
  - Phenotypic assessment of synthetic GAP1 locus
  - SCRaMbLEing the GAP1 locus
  - Media switching to assess HIS3 SPeccs
  - Online databases and resources
- QUANTIFICATION AND STATISTICAL ANALYSIS

SUPPLEMENTAL INFORMATION

Supplemental information can be found online at https://doi.org/10.1016/j.xgen.2023.100418.

ACKNOWLEDGMENTS

We would like to thank Paul Freemont, Alistair Elfick, Ian Roberts, and Anil Wipat for their support and advice; William Shaw for his help in establishing CRISPR gap repair methods; and Markus Ralser for his gift of plasmid pHLUM. This work was funded by BBSRC awards BB/K019791/1 and BB/R002614/1. B.A.B. was supported by the University of Nottingham through a Nottingham Research Fellowship.

AUTHOR CONTRIBUTIONS

Conceptualization, B.A.B., A.H., S.G.O., L.A.M., J.S.B., J.D.B., and T.E.; methodology, B.A.B., Y.Z., S.L., G.S., L.A.M., J.D.B., and T.E.; software, R.M.M., G.-O.F.G., S.G.O., L.A.M., and J.S.B.; validation, B.A.B., M.I.S., Y.Z., S.L., G.-O.F.G., and K.P.-F.; formal analysis, B.A.B., X.L., K.C., V.F., E.L., K.P.-F., and T.E.; investigation, B.A.B., X.L., M.R.M.D., D.J., M.I.S., K.C., Y.Z., S.L., and F.S.; resources, B.A.B., R.S.K.W., Y.C., G.S., L.A.M., J.S.B., J.D.B., and T.E.; data curation, B.A.B., K.C., R.M.M., G.-O.F.G., V.F., E.L., J.C., G.S., and T.E.; writing – original draft, B.A.B.; writing – review & editing, B.A.B., Y.Z., S.L., S.G.O., G.S., J.S.B., J.D.B., and T.E.; visualization, B.A.B., X.L., and M.I.S.; supervision, B.A.B., S.G.O., Y.C., G.S., J.S.B., J.D.B., and T.E.; project administration, B.A.B., J.D.B., and T.E.; funding acquisition, B.A.B., S.G.O., J.D.B., and T.E.

DECLARATION OF INTERESTS

B.A.B. is a scientific advisory board (SAB) member of Eden Bio Ltd. T.E. is a consultant to Replay Holdings, LLC and SAB member of Modern Synthesis, Inc. J.D.B. is a founder and director of CDI Labs, Inc.; a founder of and consultant to Neochromosome, Inc.; a consultant to Opentrons Labworks, Inc.; a founder and SAB member of and consultant to ReOpen Diagnostics, LLC; and serves or served on the SAB of the following: Sangamo, Inc.; Modern Meadow, Inc.; Rome Therapeutics, Inc.; Sample6, Inc.; Tessera Therapeutics, Inc.; and the Wyss Institute. L.A.M. is a founder of Neochromosome, Inc. and an employee of Opentrons Labworks, Inc.

Received: October 11, 2022
Revised: July 13, 2023
Accepted: September 8, 2023
Published: November 8, 2023

REFERENCES

1. Fredens, J., Wang, K., de la Torre, D., Funke, L.F.H., Robertson, W.E., Christova, Y., Chia, T., Schmied, W.H., Dunkelmann, D.L., Beránek, V., et al. (2019). Total synthesis of Escherichia coli with a recoded genome. Nature *569*, 514–518. https://doi.org/10.1038/s41586-019-1192-5.
2. Gibson, D.G., Glass, J.I., Lartigue, C., Noskov, V.N., Chuang, R.Y., Algire, M.A., Benders, G.A., Montague, M.G., Ma, L., Moodie, M.M., et al. (2010). Creation of a Bacterial Cell Controlled by a Chemically Synthesized Genome. Science *329*, 52–56. https://doi.org/10.1126/science.1190719.
3. Alper, H., Fischer, C., Nevoigt, E., and Stephanopoulos, G. (2005). Tuning genetic control through promoter engineering. Proc. Natl. Acad. Sci. USA *102*, 12678–12683. https://doi.org/10.1073/pnas.0504604102.
4. Marc, P., Margeot, A., Devaux, F., Blugeon, C., Corral-Debrinski, M., and Jacq, C. (2002). Genome-wide analysis of mRNAs targeted to yeast mitochondria. EMBO Rep. *3*, 159–164. https://doi.org/10.1093/embo-reports/kvf025.
5. Pleiss, J.A., Whitworth, G.B., Bergkessel, M., and Guthrie, C. (2007). Transcript specificity in yeast pre-mRNA splicing revealed by mutations in core spliceosomal components. PLoS Biol *5*, 745–757. https://doi.org/10.1371/journal.pbio.0050090.
6. Yu, C.H., Dang, Y., Zhou, Z., Wu, C., Zhao, F., Sachs, M.S., and Liu, Y. (2015). Codon Usage Influences the Local Rate of Translation Elongation to Regulate Co-translational Protein Folding. Mol. Cell *59*, 744–754. https://doi.org/10.1016/j.molcel.2015.07.018.
7. Mercy, G., Mozziconacci, J., Scolari, V.F., Yang, K., Zhao, G., Thierry, A., Luo, Y., Mitchell, L.A., Shen, M., Shen, Y., et al. (2017). 3D organization of synthetic and scrambled chromosomes. Science *355*, eaaf4597. https://doi.org/10.1126/science.aaf4597.
8. Duan, Z., Andronescu, M., Schutz, K., McIlwain, S., Kim, Y.J., Lee, C., Shendure, J., Fields, S., Blau, C.A., and Noble, W.S. (2010). A three-dimensional model of the yeast genome. Nature *465*, 363–367. https://doi.org/10.1038/nature08973.
9. Garvie, C.W., and Wolberger, C. (2001). Recognition of specific DNA sequences. Mol. Cell *8*, 937–946. https://doi.org/10.1016/s1097-2765(01)00392-6.
10. Dymond, J.S., Richardson, S.M., Coombes, C.E., Babatz, T., Muller, H., Annaluru, N., Blake, W.J., Schwerzmann, J.W., Dai, J., Lindstrom, D.L., et al. (2011). Synthetic chromosome arms function in yeast and generate phenotypic diversity by design. Nature *477*, 471–476. https://doi.org/10.1038/nature10403.
11. Shen, Y., Stracquadanio, G., Wang, Y., Yang, K., Mitchell, L.A., Xue, Y., Cai, Y., Chen, T., Dymond, J.S., Kang, K., et al. (2016). SCRaMbLE generates designed combinatorial stochastic diversity in synthetic chromosomes. Genome Res. *26*, 36–49. https://doi.org/10.1101/gr.193433.115.
12. Blount, B.A., Gowers, G.O.F., Ho, J.C.H., Ledesma-Amaro, R., Jovicevic, D., McKiernan, R.M., Xie, Z.X., Li, B.Z., Yuan, Y.J., and Ellis, T. (2018). Rapid host strain improvement by in vivo rearrangement of a synthetic yeast chromosome. Nat. Commun. *9*, 1932. https://doi.org/10.1038/s41467-018-03143-w.
13. Liu, W., Luo, Z., Wang, Y., Pham, N.T., Tuck, L., Pérez-Pi, I., Liu, L., Shen, Y., French, C., Auer, M., et al. (2018). Rapid pathway prototyping and engineering using in vitro and in vivo synthetic genome SCRaMbLE-in methods. Nat. Commun. *9*, 1936. https://doi.org/10.1038/s41467-018-04254-0.
14. Luo, Z., Wang, L., Wang, Y., Zhang, W., Guo, Y., Shen, Y., Jiang, L., Wu, Q., Zhang, C., Cai, Y., and Dai, J. (2018). Identifying and characterizing SCRaMbLEd synthetic yeast using ReSCuES. Nat. Commun. *9*, 1930. https://doi.org/10.1038/s41467-017-00806-y.
15. Luo, Z., Yu, K., Xie, S., Monti, M., Schindler, D., Fang, Y., Zhao, S., Liang, Z., Jiang, S., Luan, M., et al. (2021). Compacting a synthetic yeast chromosome arm. Genome Biol. *22*, 5. https://doi.org/10.1186/s13059-020-02232-8.
16. Richardson, S.M., Mitchell, L.A., Stracquadanio, G., Yang, K., Dymond, J.S., DiCarlo, J.E., Lee, D., Huang, C.L.V., Chandrasegaran, S., Cai, Y., et al. (2017). Design of a synthetic yeast genome. Science *355*, 1040–1044. https://doi.org/10.1126/science.aaf4557.
17. Schindler, D., Walker, R.S.K., Jiang, S., Brooks, A.N., Wang, Y., Muller, C.A., Cockram, C., Luo, Y., Garcia, A., Schraivogel, D., et al. (2022). Design, Construction, and Functional Characterization of a tRNA Neochromosome in Yeast. Preprint at bioRxiv. https://doi.org/10.1101/2022.10.03.510608.
18. Annaluru, N., Muller, H., Mitchell, L.A., Ramalingam, S., Stracquadanio, G., Richardson, S.M., Dymond, J.S., Kuang, Z., Scheifele, L.Z., Cooper, E.M., et al. (2014). Total Synthesis of a Functional Designer Eukaryotic Chromosome. Science *344*, 55–58. https://doi.org/10.1126/science.1249252.
19. Carroll, S.M., Derose, M.L., Gaudray, P., Moore, C.M., Needham-Vandevanter, D.R., Vonhoff, D.D., and Wahl, G.M. (1988). Double minute chromosomes can be produced from precursors derived from a chromosomal deletion. Mol. Cell Biol. *8*, 1525–1533. https://doi.org/10.1128/mcb.8.4.1525-1533.1988.
20. Dillon, L.W., Kumar, P., Shibata, Y., Wang, Y.H., Willcox, S., Griffith, J.D., Pommier, Y., Takeda, S., and Dutta, A. (2015). Production of Extrachromosomal MicroDNAs Is Linked to Mismatch Repair Pathways and Transcriptional Activity. Cell Rep. *11*, 1749–1759. https://doi.org/10.1016/j.celrep.2015.05.020.
21. van Loon, N., Miller, D., and Murnane, J.P. (1994). Formation of extrachromosomal circular DNA in HeLa cells by nonhomologous recombination. Nucleic Acids Res. *22*, 2447–2452. https://doi.org/10.1093/nar/22.13.2447.
22. Møller, H.D., Parsons, L., Jorgensen, T.S., Botstein, D., and Regenberg, B. (2015). Extrachromosomal circular DNA is common in yeast. Proc. Natl. Acad. Sci. USA *112*, E3114–E3122. https://doi.org/10.1073/pnas.1508825112.
23. deCarvalho, A.C., Kim, H., Poisson, L.M., Winn, M.E., Mueller, C., Cherba, D., Koeman, J., Seth, S., Protopopov, A., Felicella, M., et al. (2018).

Discordant inheritance of chromosomal and extrachromosomal DNA elements contributes to dynamic disease evolution in glioblastoma. Nat. Genet. *50*, 708–717. https://doi.org/10.1038/s41588-018-0105-0.

24. Denoth-Lippuner, A., Krzyzanowski, M.K., Stober, C., and Barral, Y. (2014). Role of SAGA in the asymmetric segregation of DNA circles during yeast ageing. Elife *3*, e03790. https://doi.org/10.7554/eLife.03790.
25. Ling, X., Han, Y., Meng, J., Zhong, B., Chen, J., Zhang, H., Qin, J., Pang, J., and Liu, L. (2021). Small extrachromosomal circular DNA (eccDNA): major functions in evolution and cancer. Mol. Cancer *20*, 113. https://doi.org/10.1186/s12943-021-01413-8.
26. Hull, R.M., King, M., Pizza, G., Krueger, F., Vergara, X., and Houseley, J. (2019). Transcription-induced formation of extrachromosomal DNA during yeast ageing. PLoS Biol. *17*, e3000471. https://doi.org/10.1371/journal.pbio.3000471.
27. Wang, Y., Wang, M., Djekidel, M.N., Chen, H., Liu, D., Alt, F.W., and Zhang, Y. (2021). eccDNAs are apoptotic products with high innate immunostimulatory activity. Nature *599*, 308–314. https://doi.org/10.1038/s41586-021-04009-w.
28. Paulsen, T., Kumar, P., Koseoglu, M.M., and Dutta, A. (2018). Discoveries of Extrachromosomal Circles of DNA in Normal and Tumor Cells. Trends Genet. *34*, 270–278. https://doi.org/10.1016/j.tig.2017.12.010.
29. Wu, S., Turner, K.M., Nguyen, N., Raviram, R., Erb, M., Santini, J., Luebeck, J., Rajkumar, U., Diao, Y., Li, B., et al. (2019). Circular ecDNA promotes accessible chromatin and high oncogene expression. Nature *575*, 699–703. https://doi.org/10.1038/s41586-019-1763-5.
30. Turner, K.M., Deshpande, V., Beyter, D., Koga, T., Rusert, J., Lee, C., Li, B., Arden, K., Ren, B., Nathanson, D.A., et al. (2017). Extrachromosomal oncogene amplification drives tumour evolution and genetic heterogeneity. Nature *543*, 122–125. https://doi.org/10.1038/nature21356.
31. Nikolaev, S., Santoni, F., Garieri, M., Makrythanasis, P., Falconnet, E., Guipponi, M., Vannier, A., Radovanovic, I., Bena, F., Forestier, F., et al. (2014). Extrachromosomal driver mutations in glioblastoma and low-grade glioma. Nat. Commun. *5*, 5690. https://doi.org/10.1038/ncomms6690.
32. Koche, R.P., Rodriguez-Fos, E., Helmsauer, K., Burkert, M., MacArthur, I.C., Maag, J., Chamorro, R., Munoz-Perez, N., Puiggròs, M., Dorado Garcia, H., et al. (2020). Extrachromosomal circular DNA drives oncogenic genome remodeling in neuroblastoma. Nat. Genet. *52*, 29–34. https://doi.org/10.1038/s41588-019-0547-z.
33. Zhu, Y., Gujar, A.D., Wong, C.H., Tjong, H., Ngan, C.Y., Gong, L., Chen, Y.A., Kim, H., Liu, J., Li, M., et al. (2021). Oncogenic extrachromosomal DNA functions as mobile enhancers to globally amplify chromosomal transcription. Cancer Cell *39*, 694–707.e7. https://doi.org/10.1016/j.ccell.2021.03.006.
34. Whale, A.J., King, M., Hull, R.M., Krueger, F., and Houseley, J. (2022). Stimulation of adaptive gene amplification by origin firing under replication fork constraint. Nucleic Acids Res. *50*, 915–936. https://doi.org/10.1093/nar/gkab1257.
35. Winzeler, E.A., Shoemaker, D.D., Astromoff, A., Liang, H., Anderson, K., Andre, B., Bangham, R., Benito, R., Boeke, J.D., Bussey, H., et al. (1999). Functional characterization of the S. cerevisiae genome by gene deletion and parallel analysis. Science *285*, 901–906. https://doi.org/10.1126/science.285.5429.901.
36. Mitchell, L.A., Wang, A., Stracquadanio, G., Kuang, Z., Wang, X., Yang, K., Richardson, S., Martin, J.A., Zhao, Y., Walker, R., et al. (2017). Synthesis, debugging, and effects of synthetic chromosome consolidation: synVI and beyond. Science *355*, eaaf4831. https://doi.org/10.1126/science.aaf4831.
37. Hill, A., and Bloom, K. (1987). Genetic manipulation of centromere function. Mol. Cell Biol. *7*, 2397–2405. https://doi.org/10.1128/mcb.7.7.2397-2405.1987.
38. Palacios-Flores, K., García-Sotelo, J., Castillo, A., Uribe, C., Aguilar, L., Morales, L., Gómez-Romero, L., Reyes, J., Garciarubio, A., Boege, M., and Dávila, G. (2018). A Perfect Match Genomic Landscape Provides a Unified Framework for the Precise Detection of Variation in Natural and Synthetic Haploid Genomes. Genetics *208*, 1631–1641. https://doi.org/10.1534/genetics.117.300589.
39. Kitakawa, M., Grohmann, L., Graack, H.R., and Isono, K. (1990). Cloning and Characterization of Nuclear Genes for 2 Mitochondrial Ribosomal-Proteins in *Saccharomyces cerevisiae*. Nucleic Acids Res. *18*, 1521–1529. https://doi.org/10.1093/nar/18.6.1521.
40. Zhang, H., and Singh, K.K. (2014). Global genetic determinants of mitochondrial DNA copy number. PLoS One *9*, e105242. https://doi.org/10.1371/journal.pone.0105242.
41. Bohovych, I., Fernandez, M.R., Rahn, J.J., Stackley, K.D., Bestman, J.E., Anandhan, A., Franco, R., Claypool, S.M., Lewis, R.E., Chan, S.S.L., and Khalimonchuk, O. (2015). Metalloprotease OMA1 Fine-tunes Mitochondrial Bioenergetic Function and Respiratory Supercomplex Stability. Sci. Rep. *5*, 13989. https://doi.org/10.1038/srep13989.
42. Wang, H., Li, Q., Zhang, Z., Zhou, C., Ayepa, E., Abrha, G.T., Han, X., Hu, X., Yu, X., Xiang, Q., et al. (2019). YKL107W from *Saccharomyces cerevisiae*. Appl. Microbiol. Biotechnol. *103*, 5699–5713. https://doi.org/10.1007/s00253-019-09885-x.
43. Sertil, O., Cohen, B.D., Davies, K.J., and Lowry, C.V. (1997). The *DAN1* gene of *S. cerevisiae*. Gene X. *192*, 199–205. https://doi.org/10.1016/s0378-1119(97)00028-0.
44. Domitrovic, T., Kozlov, G., Freire, J.C.G., Masuda, C.A., da Silva Almeida, M., Montero-Lomeli, M., Atella, G.C., Matta-Camacho, E., Gehring, K., and Kurtenbach, E. (2010). Structural and functional study of YER067W, a new protein involved in yeast metabolism control and drug resistance. PLoS One *5*, e11163. https://doi.org/10.1371/journal.pone.0011163.
45. Gresham, D., Usaite, R., Germann, S.M., Lisby, M., Botstein, D., and Regenberg, B. (2010). Adaptation to diverse nitrogen-limited environments by deletion or extrachromosomal element formation of the *GAP1*. Proc. Natl. Acad. Sci. USA *107*, 18551–18556. https://doi.org/10.1073/pnas.1014023107.
46. Møller, H.D., Andersen, K.S., and Regenberg, B. (2013). A model for generating several adaptive phenotypes from a single genetic event: Saccharomyces cerevisiae GAP1 as a potential bet-hedging switch. Commun. Integr. Biol. *6*, e23933. https://doi.org/10.4161/cib.23933.
47. Jauniaux, J.C., and Grenson, M. (1990). Gap1, the General Amino-Acid Permease Gene of *Saccharomyces cerevisiae*. Eur. J. Biochem. *190*, 39–44. https://doi.org/10.1111/j.1432-1033.1990.tb15542.x.
48. – Arrey, G., Keating, S.T., and Regenberg, B. (2022). A unifying model for extrachromosomal circular DNA load in eukaryotic cells. Semin. Cell Dev. Biol. *128*, 40–50. https://doi.org/10.1016/j.semcdb.2022.03.002.
49. Hua, S.B., Qiu, M., Chan, E., Zhu, L., and Luo, Y. (1997). Minimum length of sequence homology required for in vivo cloning by homologous recombination in yeast. Plasmid *38*, 91–96. https://doi.org/10.1006/plas.1997.1305.
50. Quénet, D., and Dalal, Y. (2012). The CENP-A nucleosome: a dynamic structure and role at the centromere. Chromosome Res *20*, 465–479. https://doi.org/10.1007/s10577-012-9301-4.
51. Henikoff, S., Ramachandran, S., Krassovsky, K., Bryson, T.D., Codomo, C.A., Brogaard, K., Widom, J., Wang, J.-P., and Henikoff, J.G. (2014). The budding yeast Centromere DNA Element II wraps a stable Cse4 hemisome in either orientation in vivo. Elife *3*, e01861. https://doi.org/10.7554/eLife.01861.
52. Kreike, J., Schulze, M., Pillar, T., Körte, A., and Rödel, G. (1986). Cloning of a Nuclear Gene *MRS1* Involved in the Excision of a Single Group-I Intron (BI3) from the Mitochondrial Cob Transcript in *Saccharomyces cerevisiae*. Curr. Genet. *11*, 185–191. https://doi.org/10.1007/BF00420605.
53. Saint-Georges, Y., Garcia, M., Delaveau, T., Jourdren, L., Le Crom, S., Lemoine, S., Tanty, V., Devaux, F., and Jacq, C. (2008). Yeast mitochondrial biogenesis: a role for the PUF RNA-binding protein Puf3p in mRNA localization. PLoS One *3*, e2293. https://doi.org/10.1371/journal.pone.0002293.

54. Foat, B.C., Houshmandi, S.S., Olivas, W.M., and Bussemaker, H.J. (2005). Profiling condition-specific, genome-wide regulation of mRNA stability in yeast. Proc. Natl. Acad. Sci. USA *102*, 17675–17680. https://doi.org/10.1073/pnas.0503803102.

55. Gerber, A.P., Herschlag, D., and Brown, P.O. (2004). Extensive association of functionally and cytotopically related mRNAs with Puf family RNA-binding proteins in yeast. PLoS Biol. *2*, E79–E354. https://doi.org/10.1371/journal.pbio.0020079.

56. Williams, T.C., Krokamp, H., Xu, X., Wightman, E.L.I., Llorente, B., Borneman, A.R., Carpenter, A.C., Van Wyk, N., Espinosa, M.i., Daniel, E.L., et al. (2022). Laboratory evolution and polyploid SCRaMbLE reveal genomic plasticity to synthetic chromosome defects and rearrangements. Preprint at bioRxiv. https://doi.org/10.1101/2022.07.22.501046.

57. Shen, Y., Wang, Y., Chen, T., Gao, F., Gong, J., Abramczyk, D., Walker, R., Zhao, H., Chen, S., Liu, W., et al. (2017). Deep functional analysis of synII, a 770-kilobase synthetic yeast chromosome. Science *355*, eaaf4791. https://doi.org/10.1126/science.aaf4791.

58. Wu, Y., Li, B.Z., Zhao, M., Mitchell, L.A., Xie, Z.X., Lin, Q.H., Wang, X., Xiao, W.H., Wang, Y., Zhou, X., et al. (2017). Bug mapping and fitness testing of chemically synthesized chromosome X. Science *355*, aaf4706. https://doi.org/10.1126/science.aaf4706.

59. Xie, Z.X., Li, B.Z., Mitchell, L.A., Wu, Y., Qi, X., Jin, Z., Jia, B., Wang, X., Zeng, B.X., Liu, H.M., et al. (2017). "Perfect" designer chromosome V and behavior of a ring derivative. Science *355*, eaaf4704. https://doi.org/10.1126/science.aaf4704.

60. Sinclair, D.A., and Guarente, L. (1997). Extrachromosomal rDNA circles–a cause of aging in yeast. Cell *91*, 1033–1042. https://doi.org/10.1016/s0092-8674(00)80493-6.

61. Regenberg, B., and Hansen, J. (2000). *GAP1*, a novel selection and counter-selection marker for multiple gene disruptions in *Saccharomyces cerevisiae*. Yeast *16*, 1111–1119. https://doi.org/10.1002/1097-0061(20000915)16:12<1111::AID-YEA611>3.0.CO;2-3.

62. Noer, J.B., Hørsdal, O.K., Xiang, X., Luo, Y., and Regenberg, B. (2022). Extrachromosomal circular DNA in cancer: history, current knowledge, and methods. Trends Genet. *38*, 766–781. https://doi.org/10.1016/j.tig.2022.02.007.

63. Demeke, M.M., Foulquié-Moreno, M.R., Dumortier, F., and Thevelein, J.M. (2015). Rapid evolution of recombinant *Saccharomyces cerevisiae*. PLoS Genet. *11*, e1005010. https://doi.org/10.1371/journal.pgen.1005010.

64. Grant, S.G., Jessee, J., Bloom, F.R., and Hanahan, D. (1990). Differential Plasmid Rescue from Transgenic Mouse DNAs into *Escherichia coli*. Proc. Natl. Acad. Sci. USA *87*, 4645–4649. https://doi.org/10.1073/pnas.87.12.4645.

65. Brachmann, C.B., Davies, A., Cost, G.J., Caputo, E., Li, J., Hieter, P., and Boeke, J.D. (1998). Designer deletion strains derived from Saccharomyces cerevisiae S288C: a useful set of strains and plasmids for PCR-mediated gene disruption and other applications. Yeast *14*, 115–132. https://doi.org/10.1002/(SICI)1097-0061(19980130)14:2<115::AID-YEA204>3.0.CO;2-2.

66. Shaw, W.M., Yamauchi, H., Mead, J., Gowers, G.O.F., Bell, D.J., Öling, D., Larsson, N., Wigglesworth, M., Ladds, G., and Ellis, T. (2019). Engineering a Model Cell for Rational Tuning of GPCR Signaling. Cell *177*, 782–796.e27. https://doi.org/10.1016/j.cell.2019.02.023.

67. Lee, M.E., DeLoache, W.C., Cervantes, B., and Dueber, J.E. (2015). A Highly Characterized Yeast Toolkit for Modular, Multipart Assembly. ACS Synth. Biol. *4*, 975–986. https://doi.org/10.1021/sb500366v.

68. Quinlan, A.R., and Hall, I.M. (2010). BEDTools: a flexible suite of utilities for comparing genomic features. Bioinformatics *26*, 841–842. https://doi.org/10.1093/bioinformatics/btq033.

69. Loman, N.J., and Quinlan, A.R. (2014). Poretools: a toolkit for analyzing nanopore sequence data. Bioinformatics *30*, 3399–3401. https://doi.org/10.1093/bioinformatics/btu555.

70. Koren, S., Walenz, B.P., Berlin, K., Miller, J.R., Bergman, N.H., and Phillippy, A.M. (2017). Canu: scalable and accurate long-read assembly via adaptive *k*-mer weighting and repeat separation. Genome Res. *27*, 722–736. https://doi.org/10.1101/gr.215087.116.

71. Liu, H., Wu, S., Li, A., and Ruan, J. (2021). SMARTdenovo: a *de novo* assembler using long noisy reads. GigaByte *2021*, gigabyte15. https://doi.org/10.46471/gigabyte.15.

72. Robinson, M.D., McCarthy, D.J., and Smyth, G.K. (2010). edgeR: a Bioconductor package for differential expression analysis of digital gene expression data. Bioinformatics *26*, 139–140. https://doi.org/10.1093/bioinformatics/btp616.

73. Castillo-Hair, S.M., Sexton, J.T., Landry, B.P., Olson, E.J., Igoshin, O.A., and Tabor, J.J. (2016). FlowCal: A User-Friendly, Open Source Software Tool for Automatically Converting Flow Cytometry Data from Arbitrary to Calibrated Units. ACS Synth. Biol. *5*, 774–780. https://doi.org/10.1021/acssynbio.5b00284.

74. Schindelin, J., Arganda-Carreras, I., Frise, E., Kaynig, V., Longair, M., Pietzsch, T., Preibisch, S., Rueden, C., Saalfeld, S., Schmid, B., et al. (2012). Fiji: an open-source platform for biological-image analysis. Nat. Methods *9*, 676–682. https://doi.org/10.1038/nmeth.2019.

75. Blount, B.A., Weenink, T., Vasylechko, S., and Ellis, T. (2012). Rational diversification of a promoter providing fine-tuned expression and orthogonal regulation for synthetic biology. PLoS One *7*, e33279. https://doi.org/10.1371/journal.pone.0033279.

76. Sikorski, R.S., and Hieter, P. (1989). A System of Shuttle Vectors and Yeast Host Strains Designed for Efficient Manipulation of DNA in *Saccharomyces cerevisiae*. Genetics *122*, 19–27. https://doi.org/10.1093/genetics/122.1.19.

77. Gibson, D.G., Young, L., Chuang, R.Y., Venter, J.C., Hutchison, C.A., and Smith, H.O. (2009). Enzymatic assembly of DNA molecules up to several hundred kilobases. Nat. Methods *6*, 343–345. https://doi.org/10.1038/nmeth.1318.

78. Bakota, L., Brandt, R., and Heinisch, J.J. (2012). Triple mammalian/yeast/bacterial shuttle vectors for single and combined Lentivirus- and Sindbis virus-mediated infections of neurons. Mol. Genet. Genom. *287*, 313–324. https://doi.org/10.1007/s00438-012-0680-1.

79. Norrander, J., Kempe, T., and Messing, J. (1983). Construction of improved M13 vectors using oligodeoxynucleotide-directed mutagenesis. Gene X. *26*, 101–106. https://doi.org/10.1016/0378-1119(83)90040-9.

80. Xie, Z.X., Mitchell, L.A., Liu, H.M., Li, B.Z., Liu, D., Agmon, N., Wu, Y., Li, X., Zhou, X., Li, B., et al. (2018). Rapid and Efficient CRISPR/Cas9-Based Mating-Type Switching of *Saccharomyces cerevisiae*. G3 (Bethesda) *8*, 173–183. https://doi.org/10.1534/g3.117.300347.

81. Cai, Y., Agmon, N., Choi, W.J., Ubide, A., Stracquadanio, G., Caravelli, K., Hao, H., Bader, J.S., and Boeke, J.D. (2015). Intrinsic biocontainment: multiplex genome safeguards combine transcriptional and recombinational control of essential yeast genes. Proc. Natl. Acad. Sci. USA *112*, 1803–1808. https://doi.org/10.1073/pnas.1424704112.

82. Blount, B.A., Driessen, M.R.M., and Ellis, T. (2016). GC Preps: Fast and Easy Extraction of Stable Yeast Genomic DNA. Sci. Rep. *6*, 26863. https://doi.org/10.1038/srep26863.

83. Huxley, C., Green, E.D., and Dunham, I. (1990). Rapid Assessment of *S. cerevisiae*. Trends Genet. *6*, 236. https://doi.org/10.1016/0168-9525(90)90190-h.

84. Whelan, W.L., Gocke, E., and Manney, T.R. (1979). The CAN1 locus of *Saccharomyces cerevisiae*: fine-structure analysis and forward mutation rates. Genetics *91*, 35–51. https://doi.org/10.1093/genetics/91.1.35.

85. Zhao, Y., Coelho, C., Hughes, A.L., Lazar-Stefanita, L., Yang, S., Brooks, A.N., Walker, R.S.K., Zhang, W., Lauer, S., Hernandez, C., et al. (2022). Debugging and consolidating multiple synthetic chromosomes reveals combinatorial genetic interactions. Preprint at bioRxiv. https://doi.org/10.1101/2022.04.11.486913.

86. Bray, N.L., Pimentel, H., Melsted, P., and Pachter, L. (2016). Near-optimal probabilistic RNA-seq quantification. Nat. Biotechnol. *34*, 525–527. https://doi.org/10.1038/nbt.3519.

87. Xu, Z., Wei, W., Gagneur, J., Perocchi, F., Clauder-Münster, S., Camblong, J., Guffanti, E., Stutz, F., Huber, W., and Steinmetz, L.M. (2009). Bidirectional promoters generate pervasive transcription in yeast. Nature *457*, 1033–1037. https://doi.org/10.1038/nature07728.

88. Mülleder, M., Capuano, F., Pir, P., Christen, S., Sauer, U., Oliver, S.G., and Ralser, M. (2012). A prototrophic deletion mutant collection for yeast metabolomics and systems biology. Nat. Biotechnol. *30*, 1176–1178. https://doi.org/10.1038/nbt.2442.

89. Slonimski, P.P., Perrodin, G., and Croft, J.H. (1968). Ethidium Bromide Induced Mutation of Yeast Mitochondria: Complete Transformation of Cells into Respiratory Deficient Non-Chromosomal "Petites". Biochem. Biophys. Res. Commun. *30*, 232–239. https://doi.org/10.1016/0006-291x(68)90440-3.

90. Skinner, S.O., Sepúlveda, L.A., Xu, H., and Golding, I. (2013). Measuring mRNA copy number in individual Escherichia coli cells using single-molecule fluorescent *in situ*. Nat. Protoc. *8*, 1100–1113. https://doi.org/10.1038/nprot.2013.066.

91. Lauer, S., Avecilla, G., Spealman, P., Sethia, G., Brandt, N., Levy, S.F., and Gresham, D. (2018). Single-cell copy number variant detection reveals the dynamics and diversity of adaptation. PLoS Biol. *16*, e3000069. https://doi.org/10.1371/journal.pbio.3000069.

92. Cherry, J.M., Hong, E.L., Amundsen, C., Balakrishnan, R., Binkley, G., Chan, E.T., Christie, K.R., Costanzo, M.C., Dwight, S.S., Engel, S.R., et al. (2012). Saccharomyces Genome Database: the genomics resource of budding yeast. Nucleic Acids Res. *40*, D700–D705. https://doi.org/10.1093/nar/gkr1029.

93. Altschul, S.F., Gish, W., Miller, W., Myers, E.W., and Lipman, D.J. (1990). Basic local alignment search tool. J. Mol. Biol. *215*, 403–410. https://doi.org/10.1016/S0022-2836(05)80360-2.

94. Karsch-Mizrachi, I., Takagi, T., and Cochrane, G.; International Nucleotide Sequence Database Collaboration (2018). The international nucleotide sequence database collaboration. Nucleic Acids Res. *46*, D48–D51. https://doi.org/10.1093/nar/gkx1097.

STAR★METHODS

KEY RESOURCES TABLE

REAGENT or RESOURCE	SOURCE	IDENTIFIER
**Bacterial and virus strains**		
Escherichia coli DH10B: F– *mcrA* Δ(*mrr-hsdRMS-mcrBC*) φ80*lacZ*ΔM15 Δ*lacX74 recA1 endA1 araD139* Δ(*ara-leu*)7697 *galU galK* λ– *rpsL*(Str^R) *nupG*	Grant et al.[64]	Thermo Scientific Cat#EC0113
**Chemicals, peptides, and recombinant proteins**		
Camptothecin	Sigma-Aldrich	Cat#C9911
Benomyl	Sigma-Aldrich	Cat#381586
6-azauracil	Sigma-Aldrich	Cat#A1757
Methyl methanesulfonate (MMS)	Sigma-Aldrich	Cat#129925
Cycloheximide	Sigma-Aldrich	Cat#C7698
Hydrogen peroxide	Sigma-Aldrich	Cat#88597
L-canavanine sulfate	Sigma-Aldrich	Cat#C9758
Ethidium bromide	Sigma-Aldrich	Cat#E8751
D-histidine	Sigma-Aldrich	Cat#H3751
β-estradiol	Thermo Scientific	Cat#L03801
**Critical commercial assays**		
Genomic-tip 100/G Kit	Qiagen	Cat#10243
NEBNext Ultra II FS DNA Library Prep Kit (NEB	New England Biolabs	Cat#E7805
g-TUBE (Covaris)	Covaris	Cat#520079
Ligation Sequencing Kit 1D R9.4	Oxford Nanopore Technologies	Cat#SQK-LSK109
NucleoSpin RNA Plus Kit	Macherey-Nagel	Cat#740984
RNA 6000 Nano Kit	Agilent	Cat#5067-1512
GoScript Reverse Transcription Kit A5001	Promega	Cat#A5001
Luna Universal qPCR Master Mix	New England Biolabs	Cat#M3003
**Deposited data**		
RNAseq read data for sXIb01	This paper	BioSample: SAMN37120063
RNAseq read data for sXIb16	This paper	BioSample: SAMN37120064
RNAseq read data for BY4742	This paper	BioSample: SAMN37120065
Nanopore genome sequencing read data for ysXIb03	This paper	BioProject ID: PRJNA1007585
Nanopore genome sequencing read data for ysXIb08	This paper	BioProject ID: PRJNA1007585
Illumina genome sequencing data for ysXIb16	This paper	BioProject ID: PRJNA351844
**Experimental models: Organisms/strains**		
S. cerevisiae BY4741: *MAT**a** his3*Δ1 *leu2*Δ0 *met15*Δ0 *ura3*Δ0	Brachmann et al.[65]	EUROSCARF: Y00000
S. cerevisiae BY4742: *MAT*α *his3*Δ1 *leu2*Δ0 *lys2*Δ0 *ura3*Δ0	Brachmann et al.[65]	EUROSCARF: Y10000
S. cerevisiae strain Y07039: *MAT**a** his3*Δ1 *leu2*Δ0 *met15*Δ0 *ura3*Δ0*YKL220C*Δ:*kanMX4*	Winzeler et al.[35]	EUROSCARF: Y07039
S. cerevisiae strain yXIb17: *MAT**a** his3*Δ1 *leu2*Δ0 *lys2*Δ0 *ura3*Δ0 *synXI*_9.11 pRS413-*chrXI*_tRNA (*HIS3*)	This paper	N/A
See Table S2 for a full list of yeast strains generated in this study	This paper	N/A

(*Continued on next page*)

Continued

REAGENT or RESOURCE	SOURCE	IDENTIFIER
Oligonucleotides		
For a list of primers used in this study, see Table S3	This paper	N/A
For a list of PCRTag primers used in this study, see Table S4	This paper	N/A
For a list of CRISPR/Cas9 oligonucleotides used in this study, see Table S5	This paper	N/A
Recombinant DNA		
Ellis Lab Yeast CRISPR Plasmids	Shaw et al.[66]	Addgene Cat#90516 - #90963
MoClo-YTK Plasmids	Lee et al.[67]	Addgene Kit #1000000061
For a list of plasmids used in this study, see Table S7	This paper	N/A
Software and algorithms		
BEDTools	Quinlan et al.[68]	https://github.com/arq5x/bedtools2
Poretools	Loman and Quinlan[69]	https://github.com/arq5x/poretools
Canu	Koren et al.[70]	https://github.com/marbl/canu
Smartdenovo	Liu et al.[71]	https://github.com/ruanjue/smartdenovo
edgeR	Robinson et al.[72]	https://doi.org/10.18129/B9.bioc.edgeR
FlowJo	Becton Dickinson	https://www.flowjo.com/solutions/flowjo/downloads
FlowCal	Castillo-Hair et al.[73]	https://pypi.org/project/FlowCal/
Fiji	Schindelin et al.[74]	https://fiji.sc/

RESOURCE AVAILABILITY

Lead contact

Further information and requests for resources and reagents should be directed to and will be fulfilled by the lead contact, Tom Ellis (t.ellis@imperial.ac.uk).

Materials availability

Yeast strains, plasmids and other reagents generated by this study are available from the lead contact upon request.

Data and code availability

- RNAseq data have been deposited at NCBI BioSample:https://www.ncbi.nlm.nih.gov/biosample/SAMN37120063, SAMN37120064, SAMN37120065. Illumina sequence data for ysXIb16 (*synXI*_9.11) have been deposited at NCBI BioProject:https://www.ncbi.nlm.nih.gov/bioproject/PRJNA351844. Nanopore sequence data for ysXIb03 (*synXI*_9.03) and ysXIb08 (*synXI*_9.08) have been deposited at NCBI BioProject:PRJNA1007585. All deposited data are publicly available as of the date of publication.
- This paper does not report original code.
- Any additional information required to reanalyze the data reported in this paper is available from the lead contact upon request.

EXPERIMENTAL MODEL AND STUDY PARTICIPANT DETAILS

Strains

The *Saccharomyces cerevisiae* strains used and generated in this study are listed in Table S2. *Escherichia coli* DH10B[64] (Thermo Scientific) was used for vector cloning and propagation. Strains generated by this study were constructed as follows:

ysXIa01 assembly recipient strain: We linearised the pRS403*TRT2* plasmid at the *HIS3* locus by digestion with NdeI and transformed the fragment into Y07039.[35] The successful integrant was strain ysXIa01.

BY4742-*CEN11** strain with galactose inducible *chrXI* loss: The 2985 bp EcoRI/HindIII restriction fragment of p*CEN11** was integrated into the *CEN11* locus of BY4742,[65] replacing the native sequence and giving strain BY4742-*CEN11**. To generate the strains used to test *CEN11* variant configurations, *CEN11* variant regions were PCR amplified from plasmids p*CEN11*_3_37_M2, p*CEN11*_3_37_M2b, pCEN11_3_37_M2c and pCEN11_3_37_M2f using primers BB877/BB878 and integrated into the BY4742-*CEN11** locus to replace the *CEN11** sequence using CRISPR/Cas9.

$GAP1_{syn}$ Synthetic *GAP1* locus strain: Plasmids pSXI_3_34_O1 and pSXI_3_34_O2 were digested with SfiI and the gel purified chunk DNA sections were ligated together. This ligated DNA was used as template for PCR amplification of the synthetic *GAP1* locus with primers BB582/BB585. The 4668 bp product was integrated into BY4741,[65] replacing the wild type *GAP1* locus, using CRISPR/Cas9.

GAP1 GFP reporter strains: Strains $GAP1_{WT}$-*yEGFP* and $GAP1_{syn}$-*yEGFP* were generated by insertion of the *PPFY1-yEGFP-TCYC1* cassette from pSV-PFY1p[75] 1118 bp upstream of the *GAP1* CDS of strains BY4741 and $GAP1_{syn}$ respectively. This was done using CRISPR/Cas9 with a repair template encoding the insertion sequence assembled from a PCR fragment encoding the *yEGFP* expression cassette amplified from pSV-PFY1p with primers XL494/XL495 and PCR fragments encoding homology arms amplified from BY4741 genomic DNA using primers XL492/XL493 and XL496/XL497. The 3 PCR products were pooled and co-transformed with the CRISPR/Cas9 DNA into the recipient strain, with the repair template being formed through *in vivo* homologous recombination combining the 3 overlapping fragments.

GAP1 deletion strains: $GAP1_{Specc}$ culture was plated onto YPD. A colony visually assessed to have no yEGFP fluorescence was isolated. $GAP1_{SPecc}$ loss and chromosomal *GAP1-yEFGP* deletion in *GAP1*Δ was confirmed by flow cytometry and PCR as in Figure S9A. For *GAP1Δ-yEGFP*, the *GAP1* gene of BY4741-$GAP1_{syn}$-*yEGFP* was deleted by CRISPR/Cas9 using repair template PCR-amplified from BY4741-$GAP1_{syn}$-*yEGFP* genomic DNA with primers XL1220, XL1215, XL1227 and XL1228, and gRNA array plasmid gXL080-gXL081.

Strain with *HIS3* in $GAP1_{syn}$-*yEGFP* landing pad: The *GAP1* gene of BY4741-$GAP1_{syn}$-*yEGFP* was replaced with *HIS3* using CRISPR/Cas9 with plasmid gXL080-gXL081 and repair template consisting of the *GAP1* upstream and downstream regions amplified from BY4741-$GAP1_{syn}$-*yEGFP* genomic DNA using primers XL1215, XL1178, XL1181 and XL1220, and a *HIS3* gene amplified from pWS173[66] using primers XL1179 and XL1180. Screening of correct edit was performed using primers YKR039W_1_syn_F, YKR039W_1_syn_R, XL1215, XL041, XL042 and XL1220.

Growth and media conditions

Unless otherwise stated, liquid yeast cultures were grown shaking at 30°C in YPD medium (10 g L-1 yeast extract, 20 g L-1 peptone, 20 g L-1 glucose). YPGal medium (10 g L-1 yeast extract, 20 g L-1 peptone, 20 g L-1 galactose) was used to induce galactose-inducible promoters. YPG medium (10 g L-1 yeast extract, 20 g L-1 peptone, 20 g L-1 glycerol) was used to assess respiratory growth. Synthetic complete medium (SC; 6.7 g L-1 yeast nitrogen base, 1.4 g L-1 yeast synthetic dropout medium supplemented with appropriate amino acids absent, 20 g L-1 glucose) was used for auxotrophic selection, or with all amino acids supplemented as a defined complete medium. Low-nitrogen medium (MG; 1.6 g L-1 yeast nitrogen base without ammino acids and ammonium sulfate, 20 g L-1 glucose, 0.35mM L-glutamine unless otherwise specified, no additional amino acid supplement) was used to provide low-nitrogen conditions. MPDHis medium (1.6 g L-1 D-histidine, 1 g l-1 L-proline, 10 g L-1 succinic acid, 6 g L-1 NaOH, 1.6 g L-1 yeast nitrogen base without ammino acids and ammonium sulfate, 20 g L-1 glucose) was used to assay Gap1 function (Regenberg and Hansen, 2000). For growth on plates, media were supplemented with 20 g L-1 agar. Where required for *kanMX4* selection, media were supplemented with G-418 disulfate solution (Formedium) to 250 μg mL-1. For testing strain fitness under various perturbations, media were supplemented with sorbitol (osmotic stress; 1M, 1.5M or 2M), camptothecin (topoisomerase inhibitor; 0.1, 0.5 or 1 μg mL-1), benomyl (microtubule inhibitor; 15 μg mL-1), 6-azauracil (transcription elongation inhibitor; 100 μg mL-1), or methyl methanesulfonate (MMS; DNA alkylating agent; 0.05%). When testing cells with cycloheximide (protein synthesis inhibitor; 10 μg mL-1) or hydrogen peroxide (H2O2; oxidative stress; μg ml-1) liquid YPD cultures were supplemented with the additive and incubated for 2 h prior to being washing with water, diluted and plated.

Luria Bertani medium was used for bacterial growth with ampicillin (100 μg mL^{-1}), kanamycin (50 μg mL^{-1}) or spectinomycin (50 μg mL^{-1}) added for selection as required.

METHOD DETAILS

Plasmid construction

The plasmids used and generated in this study are listed in Table S7. Plasmids were constructed as follows:

pRS403*TRT2* tRNA complementing integrative vector: We PCR amplified the *TRT2* CDS, along with 380p upstream and 267 bp downstream sequence, using BY4741 genomic DNA as template with primers BB210/BB211. The PCR product was cloned into the multiple cloning site of pRS403[76] as an EcoRI/XmaI restriction fragment.

pSXI_3_36_M1 edited M1 chunk vector: We performed two PCR amplifications with pSXI_3_34_M1 template DNA, using primer pairs BB286/BB287 and BB288/BB289. The products were gel purified, pooled and used as template for a PCR reaction with primers BB286 and BB289. The 831 bp product containing the desired TAG>TAA change in the *SFT1* CDS was purified and assembled into the 12870 bp NdeI digest fragment of pSXI_3_34_M1 by Gibson isothermal assembly.[77]

pSXI_3_36_M3 edited M3 chunk vector: We PCR amplified four fragments from pSXI_3_34_M3 using primer pairs BB290/BB291, BB292/BB293, BB294/BB295 and BB296/297. The BB290/BB291and BB292/BB293 products were combined and used as template for PCR amplification using primers BB290 and BB293, yielding a 2496 bp product. The BB294/BB295 and BB296/297 products were combined and used as template for PCR amplification using primers BB294 and BB297, yielding a 1812 bp product. Both

fragments, containing the TAG>TAA recoded *ECM9* and *YKR005C* regions, were purified and assembled into the 10639 bp SalI digest fragment of pSXI_3_34_M3 by Gibson isothermal assembly.

pSXI_3_37_M2 edited M2 chunk vector: We PCR amplified 4 fragments from a pSXI_3_34_M2 template. Fragment 1 was amplified with primers BB369/BB370, fragment 2 was amplified with primers BB371/BB372, fragment 3 was amplified with primers BB367/BB373 and fragment 4 was amplified with primers BB374/BB368. PCR with primers BB367/BB368 using a mixture of fragments 3 and 4 generated fragment 5, a 530 bp sequence covering *CEN11* with the downstream loxPsym site removed. Fragments 1 and 2 were ligated together using T4 DNA ligase to generate fragment 6, a 2050 bp sequence including the region 3′ of *YKR001C* CDS with a loxPsym sequence insertion. The 514 bp NsiI/SpeI restriction product of fragment 5, the 1998 bp SpeI/SexAI restriction product of fragment 6 and the 7535 bp restriction product of pSXI_3_34_M2 were ligated together with T4 DNA ligase to create pSXI_3_37_M2.

p*CEN11*_3_37b *CEN11* variant plasmid: We PCR amplified pSXI_3_34_M2 template DNA with primers BB468/BB472 to yield a 511 bp fragment, and with primers BB469/BB473 to yield a 286 bp fragment. These PCR products were ligated together with T4 DNA ligase and the ligation product was PCR amplified with primers BB468/BB469 to yield a 798 bp fragment, which was cloned into the 9285 bp NsiI/SalI pSXI_3_34_M2 restriction digest product as an NsiI/SalI restriction fragment.

p*CEN11*_3_37c *CEN11* variant plasmid: We PCR amplified p*CEN11*_3_37b template with primers BB468/BB475 to yield a 193 bp product, and with primers BB469/BB476 to yield a 571 bp product. These PCR products were ligated together with T4 DNA ligase and the ligation product was PCR amplified with primers BB468/BB469 to yield a 765 bp fragment, which was cloned into the 9285 bp NsiI/SalI pSXI_3_34_M2 restriction digest product as an NsiI/SalI restriction fragment.

p*CEN11*_3_37f *CEN11* variant plasmid: We PCR amplified pSXI_3_34_M2 template DNA with primers BB468/BB481 to yield a 511 bp fragment, and with primers BB469/BB479 to yield a 253 bp fragment. These PCR products were ligated together with T4 DNA ligase and the ligation product was PCR amplified with primers BB468/BB469 to yield a 764 bp fragment, which was cloned into the 9285 bp NsiI/SalI pSXI_3_34_M2 restriction digest product as an NsiI/SalI restriction fragment.

p*CEN11**, containing *CEN11* region with *PGAL1* and a *Kluyveromyces lactis URA3* expression cassette: The region upstream of *CEN11* was PCR amplified from BY4741 genomic DNA with primers BB433/BB434; the *K. lactis URA3* cassette was amplified from pJJH1304[78] with primers BB435/BB436; *PGAL1* was amplified from BY4741 genomic DNA with primers BB437/BB438; and *CEN11* and its downstream region were amplified with primers BB439/BB440. The *CEN11* upstream and *K. lactis URA3* PCR products were pooled and used as template for PCR with primers BB433/BB436, yielding a 2035 bp product. The *PGAL1* and *CEN11* downstream PCR products were pooled and used as template for PCR with primers BB437/BB440, yielding a 1010 bp product. In a final PCR step, the 2035 bp and 1010 bp products were pooled and amplified with primers BB433/BB440 to give a 2999 bp product encoding the modified *CEN11* region. The assembled PCR product was ligated into the multiple cloning site of pUC19[79] as an EcoRI/HindIII restriction fragment.

pRS405-*LEU2*:*URA3* marker swapper construct plasmid: *URA3* was PCR amplified from pRS406[76] with primers oLM396/oLM397 and cloned into pRS405[76] as an AflII fragment.

pSXI_3_34_J4*kanMX*, J4 chunk plasmid with *kanMX4* insertion: We modified pSXI_3_34_J4 to incorporate a *kanMX4* marker within the J4 chunk sequence by PCR amplifying the *kanMX4* marker from the genomic DNA of strain Y07039 using primers BB570 and BB571. The 1460bp product was cloned into pSXI_3_34_J4 as an XbaI/NarI restriction fragment, replacing 15 bp of *YKL053C-A*, generating plasmid pSXI_3_34_J4*kanMX*.

pYZ412 *MAT*α expression vector: the 3.4 kb *MAT*α locus from pXZX353[80] was subcloned into pRS415.[76]

p*SCW11*-*cre*-EBD-*kanMX4* SCRaMbLE plasmid with *kanMX4* marker: The vector was assembled using MoClo-YTK assembly[67] with plasmids pYKT083 (*AmpR-ColE1*), pYTK003 (ConL1), pYTK051 (*TENO1*), pYTK067 (ConR1), pYTK077 (*kanMX4*), pYTK081 (*CEN6*/*ARS4*) and plasmids with parts cloned from p*SCW11*-*cre*-EBD ([81]) encoding the *SCW11* promoter (pJCH021) and the *cre*-EBD CDS (pJCH022).

pXL007, with a tetracycline inducible mScarlet reporter in a *LEU2* integraton cassette: The vector was assembled using MoClo-YTK assembly and had a *LEU2* integration cassette containing *PRAD27*-[*TetA*-Nuclear Localisation Signal-*GAL4* activation domain]-*TADH1* Tet-On cassette, a tetO$_7$-*PPHO5*-*mScarlet*-*TTDH1* fluorescent reporter cassette and a *LEU2* marker cassette on a ColE1-*kanR* backbone.

pXL008, with a tetracycline inducible BFP reporter in a *LEU2* integraton cassette: The vector was assembled using MoClo-YTK assembly and had a *LEU2* integration cassette containing *PRAD27*-[*TetA*-Nuclear Localisation Signal-*GAL4* activation domain]-*TADH1* Tet-On cassette, a tetO$_7$-*PPHO5*-*BFP2*-*TTDH1* fluorescent reporter cassette and a *LEU2* marker cassette on a *ColE1*-*kanR* backbone.

gXL080-gXL081, encoding a CRISPR/Cas9 system with gRNAs targeting regions upstream and downstream of *GAP1*: The plasmid was constructed by amplifying and retargeting gRNA fragments from pWS3178 using primers XL1176, XL1182, XL1177 and XL501, which were subsequently cloned into vector pWS3910 by Golden Gate assembly (Shaw, W. et al., in preparation).

DNA extraction

Yeast genomic DNA for PCR screening was extracted using the GC prep method.[82] Yeast genomic DNA for genome sequencing was extracted using Genomic-tip kits (Qiagen). Plasmid DNA was isolated from bacterial hosts using QIAprep Spin Miniprep kits (Qiagen).

DNA transformations

Linear DNA for chromosomal integration and plasmid DNA was transformed into yeast recipient cells using the lithium acetate method.[18] Cells underwent heat shock at 42°C for 14 min and a 10 min recovery step in 5 mM $CaCl_2$ prior to plating on appropriate media. Plasmid DNA was introduced to *E. coli* recipient cells via electroporation with a MicroPulser (Bio-Rad).

synXI design and synthesis

The native *chrXI* sequence was edited *in silico* according to the Sc2.0 design principles to generate *synXI*.[16] The *synXI* sequence was divided into "chunk" sections, ranging from 4.8 kb to 9.8 kb in size. Each chunk was flanked by recoded recognition sites for restriction enzymes which cleave to generate non-palindromic sticky ends. The chunks were grouped into 18 "megachunks" with a letter designation. Where chunks represent units of DNA synthesis, megachunks represent units of *in vivo* assembly. With the exception of the first 2 chunks, which comprised megachunk A, chunks were assigned into groups of 5 per megachunk, with the final chunk in each megachunk having an auxotrophic marker sequence added to the 3′ end. Chunks were synthesized and cloned into bacterial vectors by GenScript Biotech (Piscataway, NJ, USA, chunks A1-C5) and by GeneArt AG (Regensburg, Germany, chunks D1-R5).

Due to a non-standard GFF notation of intron-encoding genes, the software platform used for design (Biostudio) did not identify TAG stop codons in these genes and, subsequently, erroneously included 3 TAG codons in the *synXI*_3.34 design. These TAG codons were in the intron-containing genes *SFT1* (encoded in chunk M1), *ECM9* (chunk M3) and *YKR005C* (chunk M3). These TAG stop codons were subsequently recoded to TAA in design *synXI*_3.36. As DNA synthesis had already been completed at the time of this design update, the existing M1 and M3 chunk plasmids were edited to conform to *synXI*_3.36.

synXI assembly

Chunk DNA was released from the plasmid backbone through restriction digest at the designed nonpalindromic cutting sites (see Figure 1A), separated through 1% agarose gel electrophoresis, selectively excised from the gel and purified using a QIAquick Gel Extraction kit (Qiagen). Chunks constituting each megachunk were ligated together *in vitro* with T4 DNA ligase (New England Biolabs) overnight at 16°C and then concentrated in a Concentrator Plus (Eppendorf) prior to transformation into the recipient strain. Transformant colonies were phenotypically selected for gain of the new auxotrophic marker and loss of the previous marker. After each megachunk integration, we performed PCRTag analysis to confirm replacement of native sequence with synthetic DNA and performed phenotypic growth spot assays to confirm that the synthetic sequence introduced did not cause growth defects. In this way, native chromosomal DNA was sequentially replaced with synthetic DNA following the SwAP-In approach.[10,16]

In completing integration of megachunk M, difficulties were encountered isolating a transformant with successful integration of the locus at the chunk M4-M5 junction (at gene *TOF2*), presumably due to inefficient *in vitro* restriction and ligation between DNA fragments. To integrate the missing synthetic DNA of this locus into a strain with a partial M integration, ysXIa16, marker swapper construct *LEU2*:*URA3* was inserted into the chromosomal *LEU2* marker (introduced by the prior megachunk M integration). This generated strain ysXIa17 with a functional *URA3* gene and a disrupted *LEU2*. Chunks M4 and M5 were then ligated together *in vitro* and the full-length ligation product was purified and transformed into ysXIa17. Auxotrophic screens on selective media were then carried out to select colonies for gain of *LEU2* and loss of *URA3*.

Difficulties were encountered fully integrating megachunk O. The sequence around the junction of chunks O3-O4, corresponding to genes *HFL1* and *MRS4*, consistently failed to integrate. As previously, this was presumed this to be due to inefficient *in vitro* assembly. One transformant strain, ysXIa24, was isolated with incomplete integration of megachunk O and residual wild-type sequence at the presumably problematic junction. This was co-transformed with a CRISPR/Cas9 system targeted to the *YKR051W*_1_WT_R PCRTag sequence and a repair template consisting of *in vitro* ligated and gel-purified O3 and O4 chunks. Using this approach, successfully isolation of full integrants was achieved without the need for marker swapping and subsequent auxotrophic marker integration. The resulting strain, ysXIa25, had full integration of megachunks A to O.

To increase the rate of synthetic chromosome construction, megachunks P, Q and R were iteratively integrated into a second construction strain, ysXIa26 - a strain we generated by integrating chunk O5 into BY4742. Integration of the final megachunk, megachunk R, resulted in a *URA3* auxotrophic marker proximal to the universal telomere cap of the right chromosomal arm. This was removed via CRISPR/Cas9 editing.

Prior to combining the two completed synthetic sections of *chrXI*, the wild type *CEN11* centromere region of ysXIa30 was modified to enable selective loss of this chromosome. Activating transcription from an inducible promoter upstream of a centromere has previously been shown to disrupt centromere function and cause loss of the chromosome during mitotic growth (Hill and Bloom, 1987). To implement this, the 2985 bp EcoRI/HindIII restriction fragment of p*CEN11** was integrated into the *CEN11* region of ysXIa30. Strains ysXIa25 (*MAT**a***, megachunks A-O, pRS413-*chrXI*_tRNA) and ysXIa31 (*MAT*α, megachunks O5-R, *CEN11*:*klURA3-PGAL1*) were mated to form diploid strain ysXIa32 with all of the *synXI* sequence, split between two separate chromosomes.

Switching TRT2 complementation to tRNA array

Plasmid pRS413-*chrXI*_tRNA, containing an array of the native tRNA genes of *chrXI* under the control of promoter and 3′ untranslated region (3′UTR) elements from *Ashbya gossypii* and *Eremothecium coryli*, was generated as part of the construction of a tRNA neochromosome.[17] We decided that this plasmid-based construct represented a more favorable method of tRNA gene complementation that would travel with synXI during genetic crosses.

To introduce pRS413-*chrXI*_tRNA to the *synXI* construction strain, whilst removing the *TRT2* insertion from the *HIS3* locus, strain ysXIa18 was mated with strain Y15078 (BY4742 *YKR007W:kanMX4*, EUROSCARF)[35] to generate the diploid ysXIa19. CRISPR/Cas9 mediated homologous recombination was then used to replace the *HIS3::TRT2* locus with *ΔHIS3* locus template sequence PCR-amplified from BY4741. The resultant strain was transformed with pRS413-*chrXI*_tRNA, sporulated and tetrads were dissected. Strain ysXIa22 was isolated, a haploid strain with full chromosomal megachunk A-M sequence, no *TRT2* sequence at the *HIS3* locus and the pRS413-chrXI_tRNA vector.

CRISPR/Cas9 genome editing and debugging

Unless otherwise stated, chromosomal editing using CRISPR/Cas9 was carried out using a previously described gap-repair vector system.[66] Target sequences were identified using the Benchling guide RNA design tool (http://www.benchling.com). To edit pWS082 to encode a retargeted gRNA, we amplified the vector using the phosphorylated primer BB353 and the desired retargeting primer, consisting of a 3′ sequence to bind pWS082 and a 5′ sequence encoding the retargeted gRNA region (Table S3). The exception is for the gRNAs targeted to *YKRCδ11*, *YKRCδ12* and the *yEGFP* insertion site upstream of *GAP1*, which were targeted using annealed oligonucleotides.[66] The PCR product was treated with DpnI at 37°C for 1 h to remove any template material, isolated by agarose gel electrophoresis, excised and then purified with a QIAquick Gel Extraction kit. The purified retargeted linear vector was self-ligated using T4 DNA ligase, which was then heat-inactivated. The gRNA vector piece for transformation into the recipient cell was generated by PCR amplifying the circularised retargeted vector with primers BB421 and BB422. The product underwent agarose gel electrophoresis and purification and was co-transformed into the recipient cell along with the BsmBI restriction fragment of the CRISPR/Cas9 plasmid and the repair template. For the specific CRISPR/Cas9 target sites, repair templates and primers see Table S5.

Repeat sequence condensation

To remove the excess repeats in the regions of synXI_9.01 corresponding to megachunk J, the initial strategy was to use CRISPR/Cas9 to reintroduce megachunk J DNA, replacing the multi-copy locus with a new single-copy region. To prepare strain ysXIb01 for this operation, chunk I5 was chromosomally integrated to introduce *LEU2* into *YKL069W*, and a modified chunk J4 with a *kanMX4* selection cassette was inserted into *YKL053C-A*, giving strain ysXIb02. Megachunk J was then integrated into this strain, along with cas9 and gRNAs targeting double-strand breaks to the *LEU2* and *kanMX4* markers (Figure 2C).

For repeat sequence condensation in the megachunk Q region, a *URA3* marker was inserted upstream of the repeated Q region in ysXIb04 via re-integration of chunk P5. This strain was then co-transformed with a full copy of megachunk Q and a CRISPR/Cas9 construct targeting the *URA3* marker (Figure S3D).

PFGE was used to analyze *synXI* length in selected transformants and full genome nanopore sequencing was performed to confirm extent of repeat sequence loss.

Removal of insertion sequence in TRK2

To remove the bacterial transposon-associated insertion sequences in *TRK2*, the propagation of the O3 chunk vector in the *E. coli* host was repeated, but with the cells growing slowly at 18°C to reduce burden during growth and thus reduce the chances of stress-induced transposon insertions into the vector. The chunk DNA was transformed into ysXIb08, along with a CRISPR/Cas9 construct targeting the transposase insertion sequence. From the resulting transformants strain ysXIb09 was isolated, in which the *TRK2*-transposon locus had been replaced by a single intact copy of *TRK2*.

Reinstating the *PRP16* TAA stop codon

When repairing the *HBS1-OMA1* region, along with removing the loxPsym sites associated with *MRPL20* and *OMA1*, a TAG stop codon was reintroduced into *PRP16*. To conform to the design criteria of the Sc2.0 genome, this TAG stop codon was swapped to TAA by CRISPR/Cas9-mediated recombination with a template amplified from chunk Q2. This generated chromosome *synXI*_9.11 in strain ysXIb13. This strain was backcrossed a further time with BY4742-*CEN11** and sporulated to isolate *HIS3+/URA3-MAT**a*** strain ysXIb16, which displayed good respiratory fitness (Figures 3D–3F). This strain underwent PCRTag analysis targeted to loci across *synXI* (Figures S6A and S6B) and genome sequencing to confirm the debugged sequence of *synXI*_9.11.

Mating, sporulation and tetrad isolation

Diploid yeast strains were generated by streaking strains onto YPD agar, incubating at 30°C for 2 days and then mixing patches of colonies from cells of opposite mating type together on a fresh YPD agar plate. Cells were incubated for 4 h at 30°C before restreaking onto a fresh media plate with appropriate selection.

To set up sporulation cultures of diploid strains, cells were grown for 24 h in pre-sporulation medium (10 g L^{-1} yeast extract, 20 g L^{-1} peptone, 10 g L^{-1} potassium acetate) before being washed twice in water and then resuspended in sporulation medium (10 g L^{-1} potassium acetate, 0.35 g L^{-1} yeast synthetic dropout medium and required amino acids supplemented to 0.25 x the amount added to synthetic complete medium). Sporulation cultures were incubated at 30°C for 1–5 days until spore formation was visible under a microscope.

To isolate haploid strains, 200 μL sporulated cells were washed and resuspended in 200 μL water with LongLife Zymolyase (G-Biosciences). Cells were incubated at 37°C for 10–20 min before 800 μL water was added to the cells. Digested cells were plated

onto a YPD plate and tetrads were dissected to individual spores arrayed on the plate using a SporePlay+ tetrad dissection microscope (Singer Instruments). Mating type of isolated haploids was determined by PCR with primers BB_mat_a, BB_mat_alpha and BB_mat_uni[83] (Table S3).

Ploidy determination and fixing

To assess whether strains were haploid, they were first patched onto YPD plates and incubated at 30°C for 2 days. The plates were then replica plated onto SC-Arg plates with and without L-canavanine sulfate (Sigma-Aldrich) added to a final concentration of 60 μg ml^{-1}. As survival on L-canavanine is reliant on mutation of the *CAN1* gene, a spontaneously acquired recessive trait,[84] strains with colony growth L-canavanine were assumed to be haploid.

To generate a haploid ysXIb16 strain, it was transformed with plasmid pYZ412 (expressing the *MAT*α mating locus) and sporulated. Haploidy was confirmed by L-canavanine assay.

PCRTag analysis

Genomic DNA from megachunk transformant colonies with the correct auxotrophic profile was PCR screened for gain of synthetic DNA and loss of the corresponding wild type DNA. This was done using PCRTag primers, which target the synthetic PCRTag watermarks and their wild type equivalents (Table S4). Colonies confirmed to have gained all PCRTag sequences and lost all equivalent wild type sequences were considered to be successful megachunk integrants and progressed to the next round of megachunk integration. Following megachunk integration, successful megachunk integrants underwent spot assays on YPG media at 30°C and YPD media at 30°C and 37°C.

Genome sequencing

For Illumina MiSeq genome sequencing, yeast genomic DNA was quantified by Qubit fluorometry using a dsDNA HS Assay Kit (Invitrogen). For strain ysXIb01, library prep and sequencing was performed by BaseClear BV. For other strains, whole genome sequencing libraries were generated using the NEBNext Ultra II FS DNA Library Prep Kit (New England Biolabs) and sequenced using an Illumina NextSeq 500/550 High Output Kit v2.5 (75 Cycles). The Illumina MiSeq sequencing data for ysXIb01 was analyzed using the Perfect Match Genomic Landscape strategy.[38] Sequencing data for other strains was analyzed using the Synthetic Yeast sequencing pipeline (Stracquadanio, G. et al., in preparation). Read coverage over genomic loci was determined using BEDTools[68] and normalized to average genome-wide coverage.[85]

Nanopore sequencing and analysis was performed as previously described.[12] After shearing to 20 kb by g-TUBE (Covaris), genomic DNA underwent library preparation with a Ligation Sequencing Kit 1D R9.4 (Oxford Nanopore Technologies). Libraries were analyzed on R9.4 flow cells using a MinION Mk 1B (Oxford Nanopore Technologies). Standard 48 h sequencing runs were performed with the MinKnow 1.5.5 software using local basecalling. Raw fast5 files were converted into fastq and fasta with Poretools.[69] Reads were then corrected with Canu v1.5,[70] before assembling into contiguous sequences with Smartdenovo,[71] using default flags.

Pulsed-field gel electrophoresis

Samples for pulsed-field gel electrophoresis were prepared using a CHEF Yeast Genomic DNA Plug Kit (Bio-Rad) with lyticase from *Arthrobacter luteus* (Sigma-Aldrich) and recombinant proteinase K (Roche). Samples were run on a 1% certified megabase agarose (Bio-Rad) in TAE gel using a CHEF-DR III Pulsed Field Electrophoresis System (Bio-Rad) for 24 h, at 6V cm^{-1} with a 60–120 s switch time ramp at an included angle of 120°. DNA was visualised under UV light following staining for 30 min with 0.5 μg mL^{-1} GelRed Nucleic Acid Stain (Millipore) and destaining in deionised water for 1 h.

Transcript analysis

Cells were grown in YPD or YPG medium at 30°C until mid-exponential growth phase (OD_{600} ~2). Cell culture corresponding to ~3 x 10^8 cells was harvested by centrifugation, washed in 0.8% physiological salt solution and resuspended in 500 μL solution of 1M sorbitol and 100 mM ethylenediaminetetraacetic acid (EDTA). Spheroplasts were generated by digesting cells with 50U zymolyase (Zymo Research) at 30°C for 30 min. Spheroplasts were collected by centrifugation and RNA was isolated using the NucleoSpin RNA Plus kit (Macherey-Nagel). RNA quality and integrity was determined by Qubit fluorometry using an RNA BR Assay Kit (Invitrogen), spectrophotometry with a NanoDrop (Thermo Scientific) and on a 2100 Bioanalyser using an RNA 6000 Nano Kit (Agilent). RNA sequencing was performed by Novogene Co. Briefly, mRNA was purified from total RNA with poly-T oligo-attached magnetic beads prior to cDNA synthesis, adaptor ligation and sequencing on an Illumina platform.

RNA sequencing data was analyzed using a custom pipeline. First, the Illumina unstranded paired-end reads were pre-processed by trimming adapters and removing low quality bases. Then, a reference *synXI* genome was built by replacing the wild type BY4742 *chrXI* with the *synXI*_9.11 and a reference transcriptome was created by considering only protein-coding genes. Importantly, when a gene in the synthetic chromosome was deleted, it was replaced with the corresponding wild type one; this allowed us to readily cross check for sample mislabeling, since no expression is expected from a deleted locus.

Transcriptomes and reads were used to quantify gene expression using kallisto quant with sequence based bias correction.[86] Successively, differentially expressed genes were identified with edgeR,[72] using the exactTest method with dispersion parameter set to 0.22 to account for the lack of replicates. Genes with a False Discovery Rate (FDR) less or equal to 0.01 were reported as significantly differentially expressed (Tables S9 and S10).

For the purposes of our analysis, we eliminated mating-type specific genes to compensate for mating type differences between the parental and *synXI* strains. This included transcripts of the dubious ORF *YKL177W* as it almost entirely overlaps *STE3*, which encodes the receptor for a factor pheromone in *MAT*α cells. The CDS of *FLO10* was entirely recoded by an early version of REPEATSMASHER due to the presence of highly repetitive sequence.[16] As a result, the *synXI FLO10*has < 70% identity to the wild type version. This lack of sequence similarity and the repetitive nature of the mRNA in BY4742, coupled with the extremely low expression levels typically observed in S288C-derived strains, led us to also omit *FLO10* from our analysis.

Of the 6 significantly differentially expressed genes that are located on *synXI* in ysXIb16, only *YKL107W* was deduced to have biological relevance. *GEX2* and *YKL223W* neighbor the telomeres, so it was assumed that their downregulation in ysXIb16 is due to a proximal telomeric repression effect. *YKL106C-A* is found 77 bp downstream of the *YKL107W* CDS and we assume its increased transcript level in the analysis is due to the two genes having overlapping transcripts.[87] *YKL118W* and *YKL202W* are both small dubious ORFS overlapping sequences changed in the synthetic redesign process. In the case of *YKL118W*, this is a Ty1 LTR and *YKL202W* overlaps repetitive sequence 3′ of *MNN4*.

For RT-qPCR determination of *GAP1* transcript levels, total RNA was isolated as for RNA sequencing. 2 μg of the isolated RNA was digested with DNAse I (Roche) and cDNA was synthesized using the GoScript reverse transcription kit A5001 (Promega). PCR assays were performed using the Luna Universal qPCR Master Mix (New England Biolabs) in a MasterCycler RealPlex 4 (Eppendorf. Each 20 μL qPCR reaction contained 90 ng of cDNA. Primers used for qPCR are XL1250, XL1251, XL1258 and XL1259. The fold change of gene expression was calculated using the $\Delta\Delta$Ct method, using *ACT1* as the reference gene. Two technical repeats were performed for each of three biological replicates.

Growth spot assays

Saturated overnight yeast cultures were used to inoculate 5 mL YPD cultures. Cultures were grown to mid-exponential phase, normalised to an OD_{600} of 1, pelleted by centrifugation, washed in water, pelleted again and resuspended in water. Washed normalised cells were serially diluted in water in one-in-ten steps. Diluted cells were plated in 10 μL spots onto media plates and incubated at the appropriate temperature for the assay.

Growth and fluorescence curves

Overnight cultures were harvested, washed and used to inoculate 100 μL cultures in a 96-well plate with a starting OD600 normalised to 0.02. Plates were incubated and measured in a Synergy HT Microplate Reader (Biotek) shaking at 30°C. Mean absorbance values of equivalent blank media wells were subtracted from data points. Where required, fluorescence measurements were made as absorbance measurements with an excitation wavelength of 485 nm and an emission wavelength of 528 nm. Mean fluorescence values of equivalent blank media wells were subtracted from data points. Where appropriate, strains were transformed with plasmid pHLUM[88] to confer prototrophy prior to assays.

Mitochondria assays

To determine whether loss of mitochondrial function affects cell growth and viability in a strain, cells were grown overnight and then incubated for 24 h at 30°C with or without the addition of ethidium bromide, a mitochondrial DNA depletion agent,[89] to a final concentration of 10 μg mL^{-1} cells were then diluted to an OD600 of 0.001 and then plated onto YPD agar.

To determine whether cells were ρ^0, we performed PCRs on genomic DNA templates with primer pairs targeting the mitochondrial 15S ribosomal RNA encoding *15S_RRNA*/*YNCQ0002W* (oLM394/oLM395) and the mitochondrial gene *COX2* (oLM398/oLM399). We visualised the PCR products by agarose gel electrophoresis. Product bands (634 bp for the 15S rRNA and 602 bp for *COX2*) were indicative of the mitochondrial genome being present in cells.

Flow cytometry

The yEGFP fluorescence of cells was measured by an Attune NxT Flow Cytometer (Thermo Scientific). The following settings were used for measuring the size of the cell, complexity of the cell: FSC 100 V, SSC 355 V, BL1 260 V. 10,000 events were collected for each experiment and analyzed by FlowJo and violin plots were generated using FlowCal.[73]

Fluorescence microscopy

Agarose pads were prepared by pouring a molten solution of phosphate buffer saline with 1.5% low-melt agarose onto five stacked microscope slides, covering with a final slide and solidifying under a 200 g weight for 45 min at room temperature.[90] For each sample, 2 μL of cell suspension was pipetted onto a coverslip of an imaging dish (idiTreat, μ-Dish 35 mm) and an agarose pad was placed on top. Cells in the imaging dish were visualised using a Nikon ECLIPSE Ti microscope by time-lapse imaging, with the following settings: Nosepiece, 20x, PFS on, interval 30 min, optical conf. BF and GFP, gain 1552. To process the images, Fiji[74] was used to merge GFP and gray channels, with the brightness of the GFP channel adjusted to match the gray channel.

Phenotypic assessment of synthetic GAP1 locus

To investigate the effects of the synthetic redesign of the *GAP1* locus on *GAP1* transcription, we performed a number of phenotypic tests on strain $GAP1_{syn}$, a BY4741 strain with the native *GAP1* locus replaced by the synthetic *GAP1* locus from *synXI*_9.11. Analysis of *GAP1* transcript levels of BY4741 and the $GAP1_{syn}$ strain by qPCR in SC -His -Leu -Ura -Trp and MG media showed high similarity between the strains (Figure S7). The $GAP1_{syn}$ locus had no discernible effect on strain growth in YPD, SC or MG growth media (Figure S8B).To discern any effects of the $GAP1_{syn}$ locus on *GAP1* copy number expansion, we inserted a *yEGFP* fluorescent marker gene into the region upstream of *GAP1*, in both the BY4741 and BY4741-$GAP1_{syn}$ strains, enabling use of GFP fluorescence as a proxy for *GAP1* locus copy number[91] (Figure S8A). Again, no effects were seen on strain growth due to this gene insertion (Figure S8B). We confirmed D-histidine sensitivity in strains with the *GAP1* locus variants, confirming that *GAP1* was producing a functional Gap1 protein in all strains (Figure S8C).

We next used our SCRaMbLE-derived strains to determine the effects of *GAP1* copy number on growth in rich, defined and 0.4mM L-glutamine MG media (Figure S10). In all conditions, we saw similar growth between the $GAP1_{SCRaMbLE-9}$ strain with 2 chromosomal copies of *GAP1* and the $GAP1_{syn}$ and $GAP1_{WT}$ strains with a single *GAP1* copy. The $GAP1_{SCRaMbLE-19}$ strain, which also has 2 *GAP1* copies but also has 2 additional copies of *ARS1116*, had slightly reduced growth rate in all tested media. In all media, including MG, cultures with $GAP1_{SPecc}$ showed a slower growth than strains a single-copy $GAP1_{syn}$, $GAP1_{WT}$ strains, or strains with a *GAP1* deletion. For all growth assays, yEGFP fluorescence was confirmed in $GAP1_{SPecc}$ cultures prior to inoculation and fluorescence was monitored to track the prevalence of *GAP1* loci within the population (Figure S10). At the population level, the $GAP1_{SPecc}$ cultures showed higher fluorescence than strains with 0, 1 or 2 copies of *GAP1* and *yEGFP*.

SCRaMbLEing the GAP1 locus

Strain $GAP1_{syn}$-*yEGFP*, transformed with plasmid p*SCW11*-*cre*EBD-*kanMX*4 was grown overnight in YPD media supplemented with 200 μg mL^{-1} G418S. Culture was diluted to an OD600 of 0.2 in 5 mL YPD media supplemented with 200 μg/mL G418S and grown for 4 h. SCRaMbLE was induced by addition of β-estradiol to a final concentration of 1 μM. Cultures were grown for a further 2 h before being washed twice in water and resuspended in 5 mL YPD. Cells were diluted x 10^{-3} in YPD and plated onto YPD agar plates. Plates were incubated at 30°C for 3 days. Colonies were analyzed by eye under blue light and those with increased GFP expression underwent screening to detect rearrangements at the *GAP1* locus. PCR analysis of colonies using exhaustive combinations of primers BB582, BB585, XL217, XL788, XL789, XL790, XL808 and XL809 was used to determine the structure of the *GAP1* locus for each strain.

To generate *HIS3* SPeccs by SCRaMbLE, strain *HIS3-yEGFP* underwent SCRaMbLE as above. Post-SCRaMbLE, cells were diluted x 10^{-3} and x 10^{-4} and plated onto SC HIS- agar plates. Plates were incubated at 30°C for 3 days. Colonies were analyzed by eye under blue light and those with increased GFP expression underwent screening to detect rearrangements at the *HIS3* locus. PCR analysis of colonies using exhaustive combinations of primers XL215, XL222, XL1243, XL1244, XL789, XL809 and XL790 was used to determine the structure of the *HIS3* locus for each strain.

Media switching to assess HIS3 SPeccs

To determine the effects on yEGFP fluorescence of switching between SC -His and SC media, colonies with visible GFP fluorescence from the post-SCRaMbLE screening plate were inoculated in 2 mL SC -His medium and grown overnight at 30°C. The overnight SC -His cultures were then used to inoculate 2 mL SC medium (supplemented with 20 mg/L histidine) and grown overnight at 30°C. This was repeated once more but growing cells initially in SC medium and then in SC -His. 20 μL of each overnight culture was diluted in 180 μL phosphate buffer saline to analyze single cell GFP expression using flow cytometry.

Online databases and resources

Unless otherwise stated, native *S. cerevisiae* CDSs and other DNA sequence elements were defined according to their annotations in the *Saccharomyces* Genome Database.[92] The basic local alignment search tool[93] (BLAST) was used to align DNA sequences against sequence databases.[94]

QUANTIFICATION AND STATISTICAL ANALYSIS

Statistical details of experiments are described in figure legends. Statistical analysis of RNAseq data, including determination of significance, is detailed in the "transcript analysis" method details section.

Cell Genomics, Volume *3*

Supplemental information

Synthetic yeast chromosome XI design provides a testbed for the study of extrachromosomal circular DNA dynamics

Benjamin A. Blount, Xinyu Lu, Maureen R.M. Driessen, Dejana Jovicevic, Mateo I. Sanchez, Klaudia Ciurkot, Yu Zhao, Stephanie Lauer, Robert M. McKiernan, Glen-Oliver F. Gowers, Fiachra Sweeney, Viola Fanfani, Evgenii Lobzaev, Kim Palacios-Flores, Roy S.K. Walker, Andy Hesketh, Jitong Cai, Stephen G. Oliver, Yizhi Cai, Giovanni Stracquadanio, Leslie A. Mitchell, Joel S. Bader, Jef D. Boeke, and Tom Ellis

Supplemental Information

Contents:

Figure S1 – page 1
Figure S2 – page 2
Figure S3 – page 3
Figure S4 – page 3
Figure S5 – page 4
Figure S6 – page 5
Figure S7 – page 6
Figure S8 – page 7
Figure S9 – page 8
Figure S10 – page 9
Figure S11 – page 10
Figure S12 – page 10
Figure S13 – page 11
Table S1 – page 12
Table S5 – pages 13-14
Table S6 – pages 15-16

Supplemental Figures

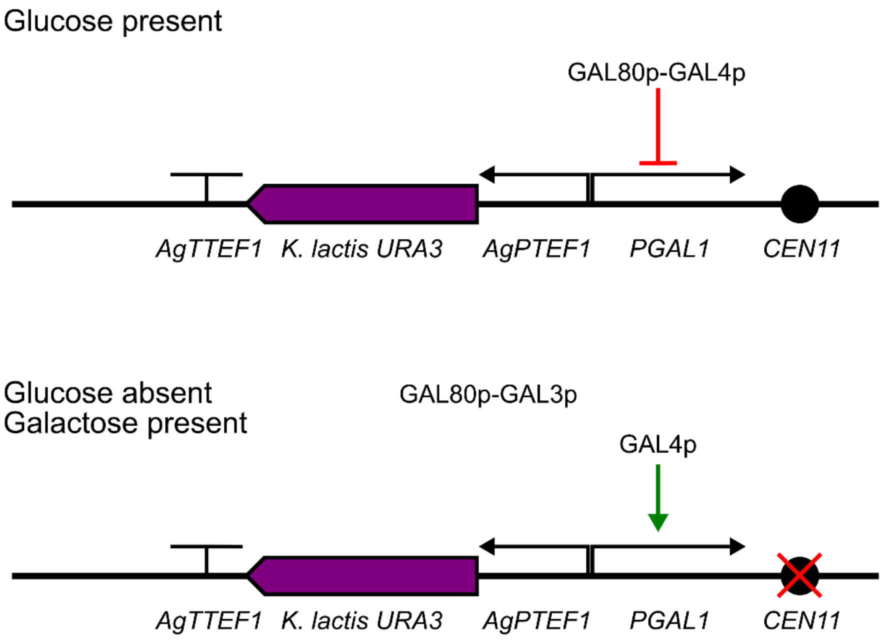

Figure S1: Schematic of *CEN11* functional disruption by the *CEN11 construct - related to Figure 1.** Overview of the *CEN11** locus under glucose repression and galactose induction. In the absence of glucose and the presence of galactose, transcription from the *GAL1* promoter (*PGAL1*) disrupts the function of *CEN11*, leading to chromosome loss through miss-segregation in mitosis. *AgTTEF1* is the *TEF1* terminator from *Ashbya gossypii*, *K. lactis URA3* is the *URA3* coding sequence from *Kluyveromyces lactis* and *AgPTEF1* is the *TEF1* promoter from *A. gossypii*. The red bar indicates transcriptional repression, the green arrow represents transcriptional activation and the red cross represents disruption of centromere function.

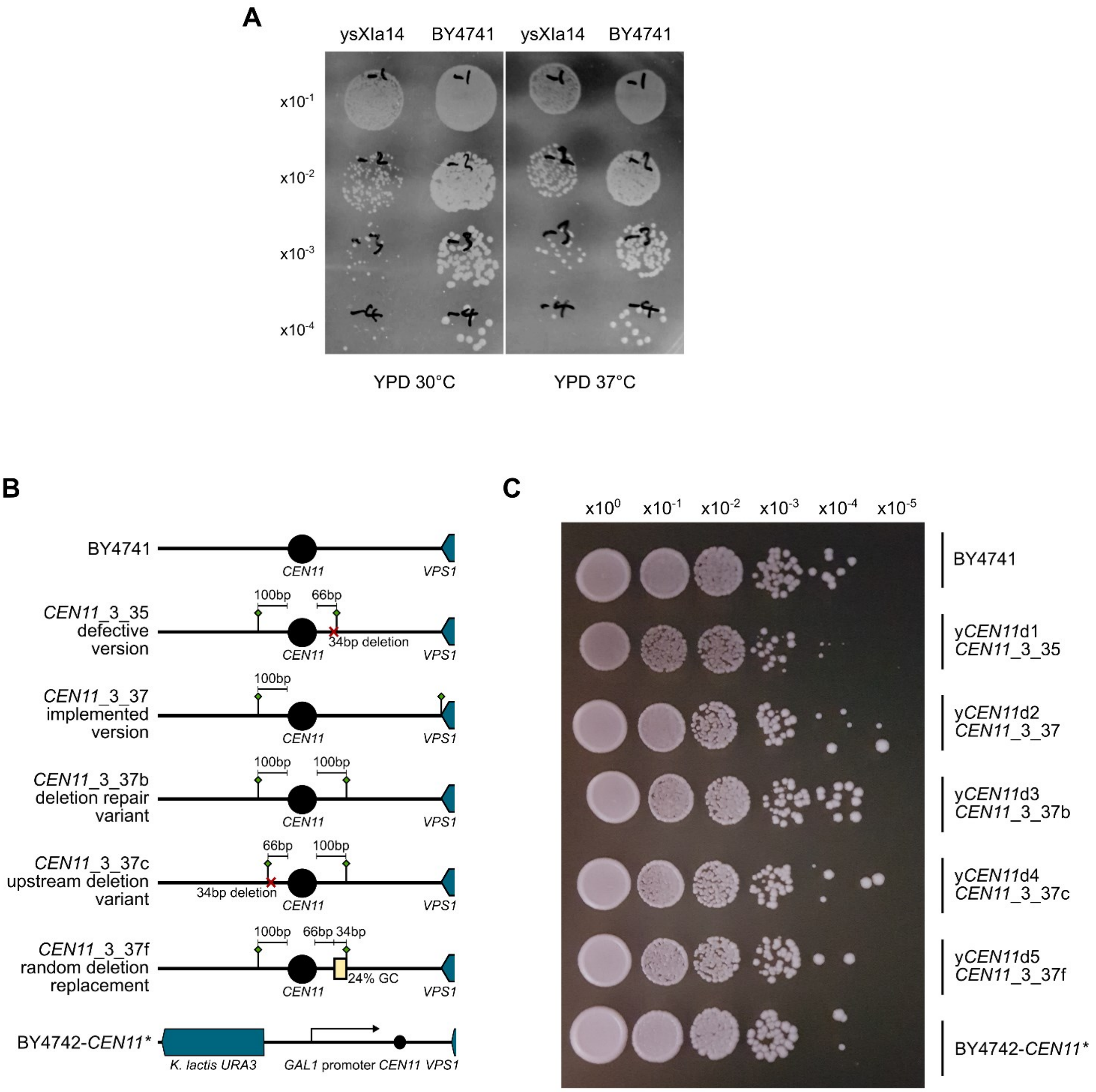

Figure S2: CEN11_3_35 causes a growth defect - related to Figure 2. (A) Growth spot assays of ysXIa14 and BY4741 parental control on YPD agar after 2 days. Incubation temperatures and spot dilution factors are indicated on the image. (B) Overview of the *CEN11* locus variants generated in this study. Green diamonds denote loxPsym sites. (C) Growth spot assays of BY4742 strains with different *CEN11* locus variants on YPD after 3 days growth at 37 °C. Strain names and *CEN11* variant numbers (where appropriate) are shown to the right. Spot dilution factors are shown above the image.

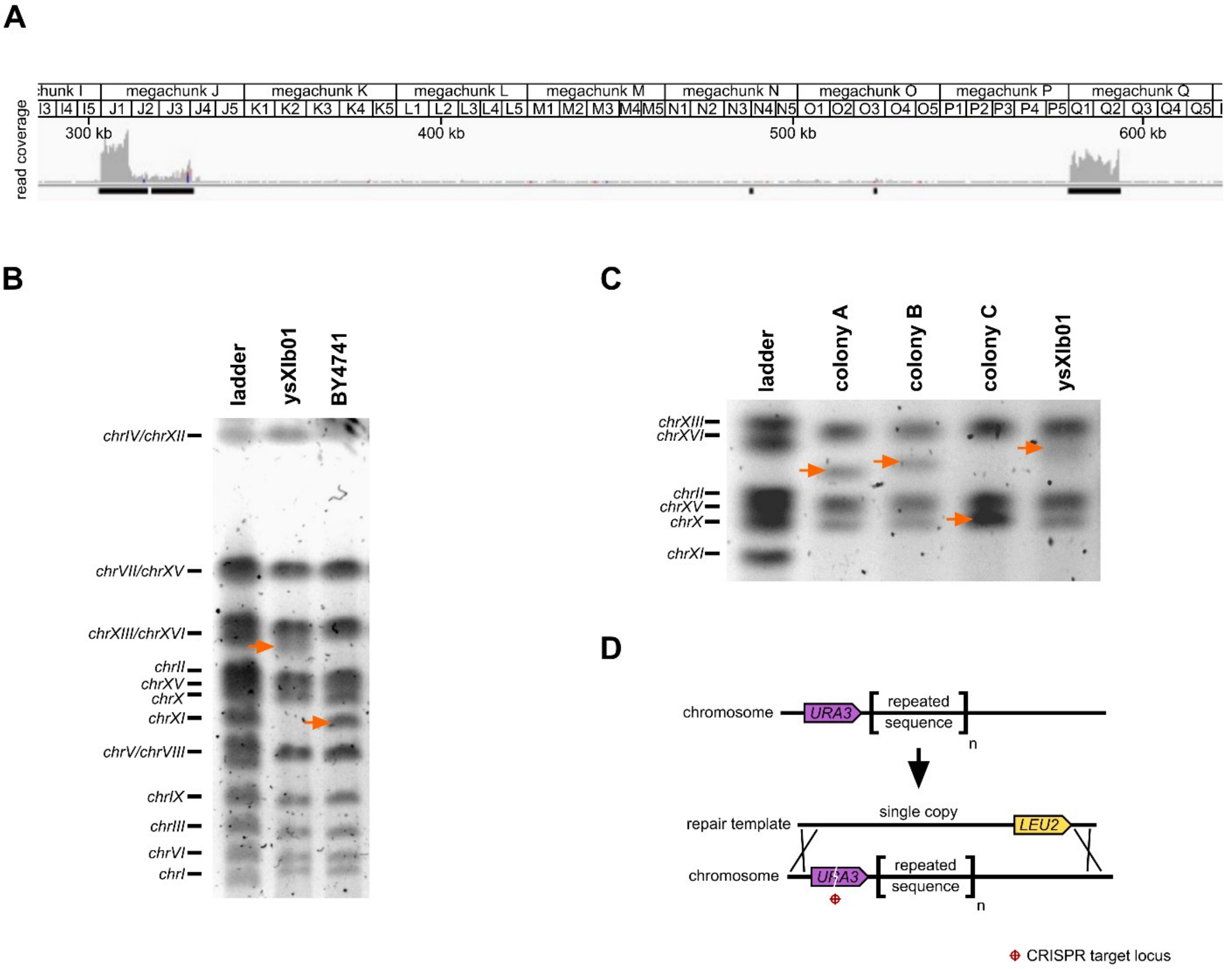

Figure S3: Repeat regions were condensed using CRISPR/Cas9 – related to Figure 2. (A) Read coverage depth from short read genome sequencing over a section of *synXI*_9.01 isolated from ysXIb01. Annotated boxes above the graph indicate the corresponding synthetic chunk and megachunk regions. (B) PFGE electrophoresis gel of genomic DNA isolated from ysXIb01 and BY4741 showing increased size of *synXI* in ysXIb01. The ladder is 0.2-2.2 Mb *S. cerevisiae* ladder (Bio-Rad). Orange arrows show the inferred positions of *chrXI* and *synXI*. (C) PFGE electrophoresis gel of genomic DNA isolated from ysXIb01 and colonies isolated after the first round of CRISPR-mediated megachunk J repeat condensation. Colony C is deduced to have the most condensation of synXI repeats. Orange arrows show the inferred position of *synXI*. (D) Strategy to condense the repeat sequence in the megachunk Q region. *URA3* is inserted upstream of the repeats before being targeted by CRISPR-Cas9 with a single copy of the region as repair template.

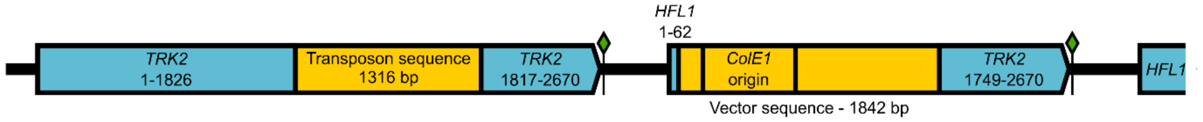

Figure S4: Schematic of the sequence insertions at the *YKR050W/TRK2* locus in ysXI_9.09 – related to Figure 2. Blue boxes represent yeast CDS sequence, yellow boxes represent sequence of bacterial origin of replication, green diamonds represent loxPsym sites. Base coordinates given for *TRK2* and *HFL1* sequences are relative to the annotated start of the coding sequence in the wildtype strain.

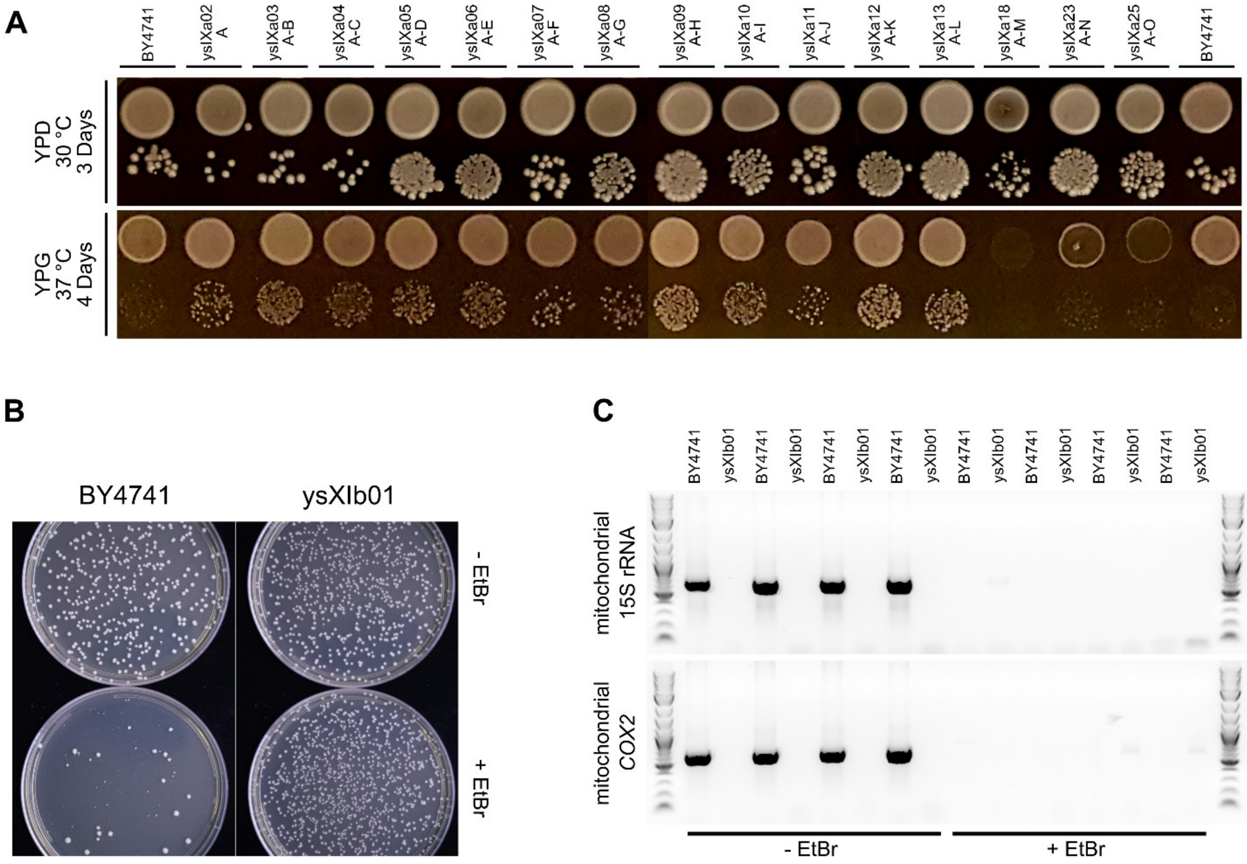

Figure S5: ysXIb01 has a mitochondrial defect - related to Figure 3. (A) Growth spot assays of *synXI* assembly intermediates following rounds of megachunk integration on YPD (glucose) and YPG (glycerol) to test respiratory function. For each strain and condition, the top spot is a x10^{-1} dilution and the bottom spot is a x10^{-3} dilution. (B) Diluted cells plated onto YPD with or without 24 hours prior exposure 10 µg ethidium bromide (EtBr). EtBr causes loss of the mitochondrial genome. (C) PCR assays targeting mitochondrial genomic DNA for amplification performed on genomic DNA harvested from colonies grown on YPD or on YPD + 10 µg EtBr. Product bands indicate the presence of mitochondrial genomic DNA. n=4 biological replicates per strain and per condition. PCR products are visualised on 1% agarose gel, with the gel image colour inverted. Ladder is 1 kb Plus DNA Ladder (New England Biolabs).

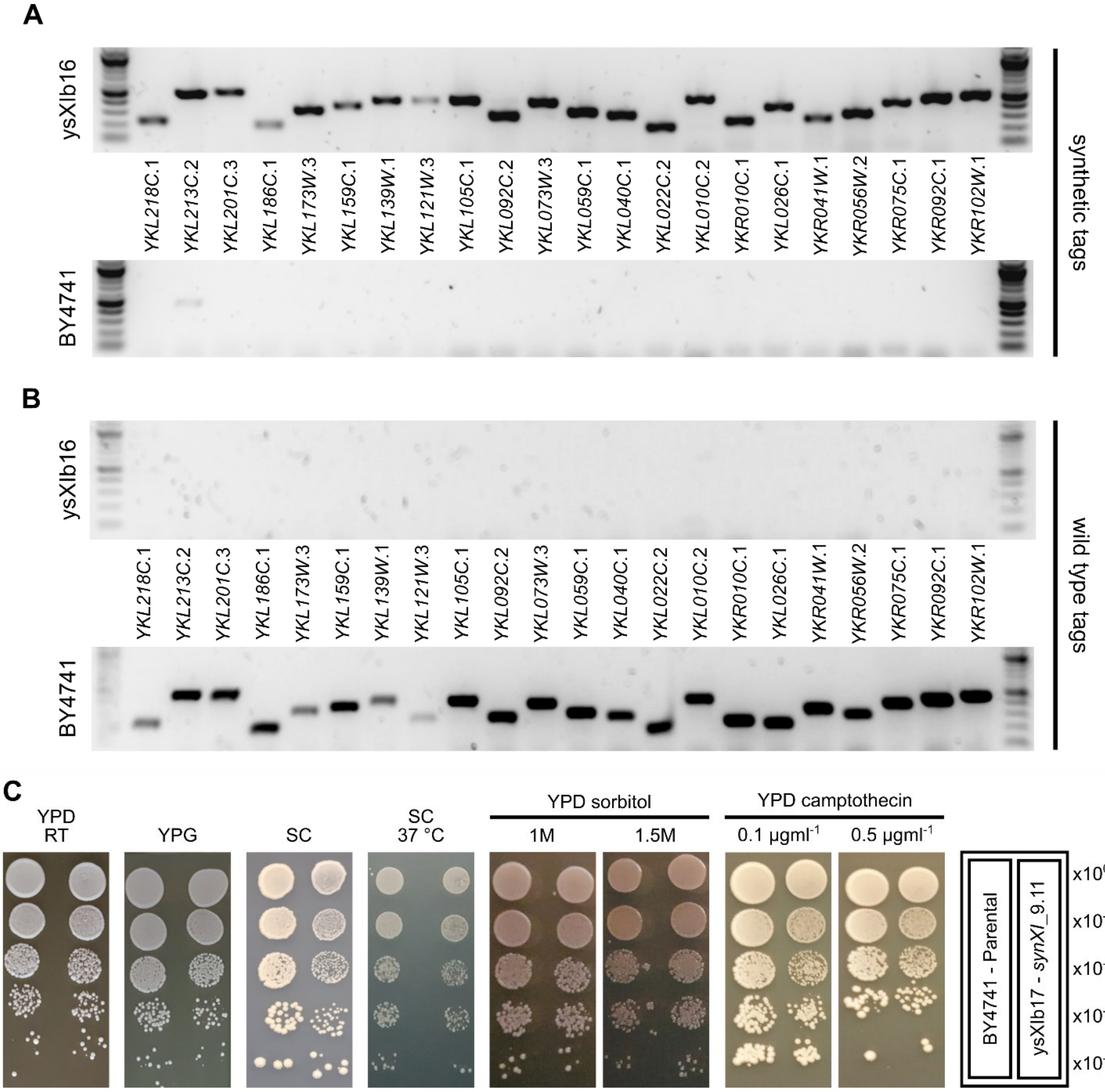

Figure S6: Genotypic and phenotypic validation of synXI strains – related to Figure 4. (A) Genomic DNA from ysXIb16 or a BY4741 control was amplified with synthetic PCRTag primer pairs. Product bands indicate the presence of synthetic DNA. (B) Genomic DNA from ysXIb16 or a BY4741 control was amplified with wild type PCRTag primer pairs. Product bands indicate the presence of wild type DNA. For panels A and B, PCR products were visualised on 1% agarose gel, with the colour inverted. PCRTag primer pair targets are labelled between the gel images, with target sequences listed in Table S4. Ladder is 1 kb Plus DNA Ladder (New England Biolabs). (C) Additional growth spot assays of ysXIb17 and a BY4741 parental control to assess cellular fitness. Serial dilutions of cultures were spotted as illustrated on the right. Unless otherwise indicated, plates were incubated at 30 °C.

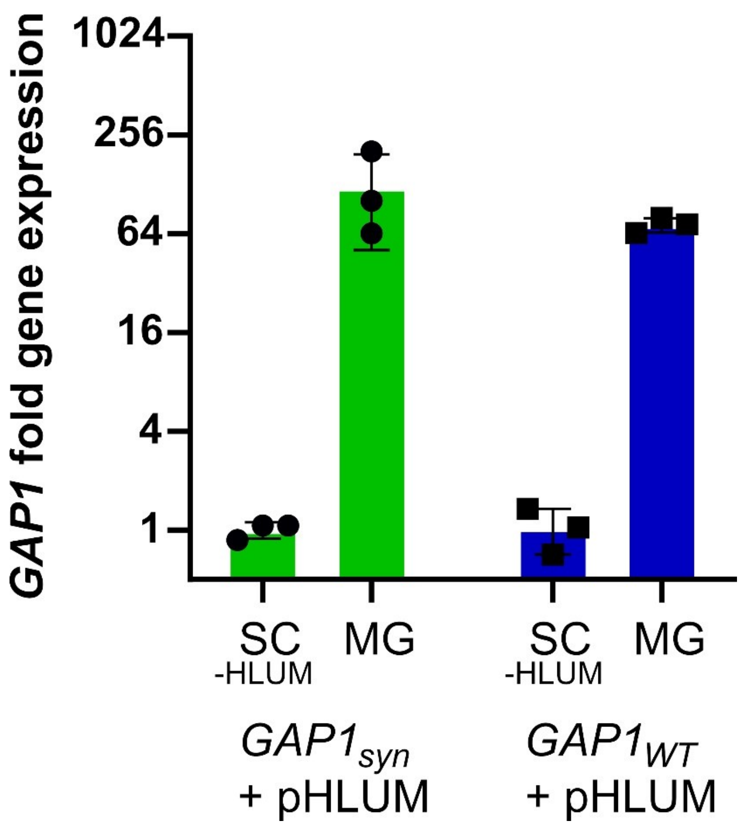

Figure S7: *GAP1*$_{syn}$ has similar transcription levels to *GAP1*$_{WT}$ - related to Figure 5. Quantification of *GAP1* transcript levels by qPCR. Strains BY4741 (*GAP1*$_{WT}$) and *GAP1syn* were transformed with pHLUM and grown for 16 hours in SC -His -Leu -Ura -Met (SC -HLUM) or MG (0.4 mM L-glutamine) before harvesting. *GAP1* transcripts were quantified by qPCR using ACT1 as a reference gene. Data is given relative to mean *GAP1* transcript abundance in *GAP*$_{WT}$ +pHLUM cells grown in SC -His -Leu -Ura -Met. Experiments were performed in biological triplicate. Individual *GAP1*$_{syn}$ data points are plotted as round dots, individual *GAP1*$_{WT}$ data points are plotted as square dots, mean averages are denoted by bar height and error bars represent standard deviation.

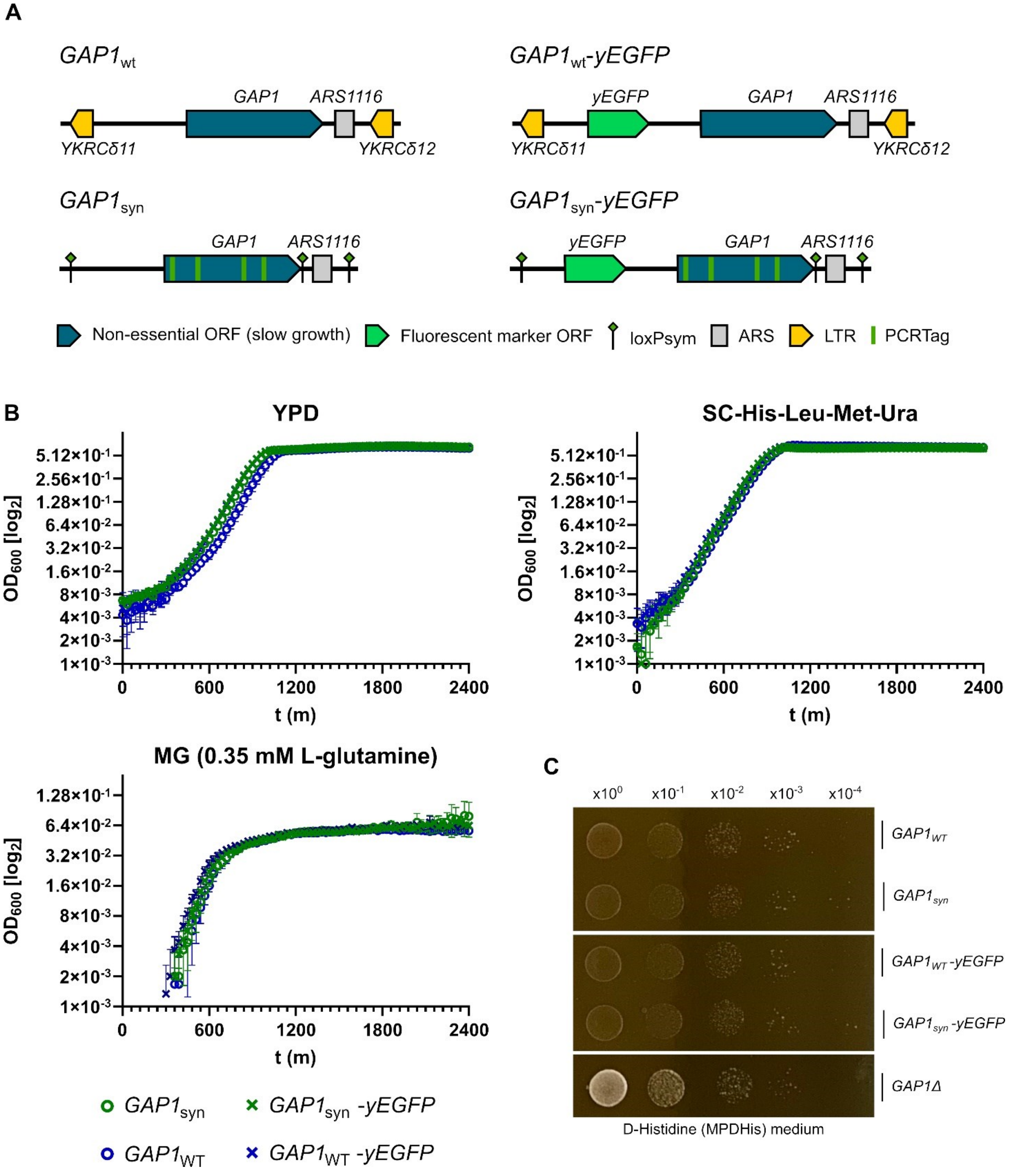

Figure S8: *GAP1$_{syn}$* produces functional Gap1 protein and does not impact growth - related to Figure 5. (A) Structure of *GAP1* loci built and integrated into BY4741 to study eccDNA dynamics. (B) Graphs show growth of BY4741 strains with different variations of the *GAP1* locus in 96 well plates under rich (YPD), defined (SC-His-Leu-Met-Ura) and low-nitrogen (MG) media conditions at 30 °C. All strains had plasmid pHLUM, making them prototrophic. Mean OD_{600} values from 3 biological replicates are plotted as circles, error bars represent standard deviation. (C) Spot growth assay on MDPHis medium containing D-histidine. Plates were incubated at 30 °C for 2 days. Image panels are all taken from a single image of one assay plate.

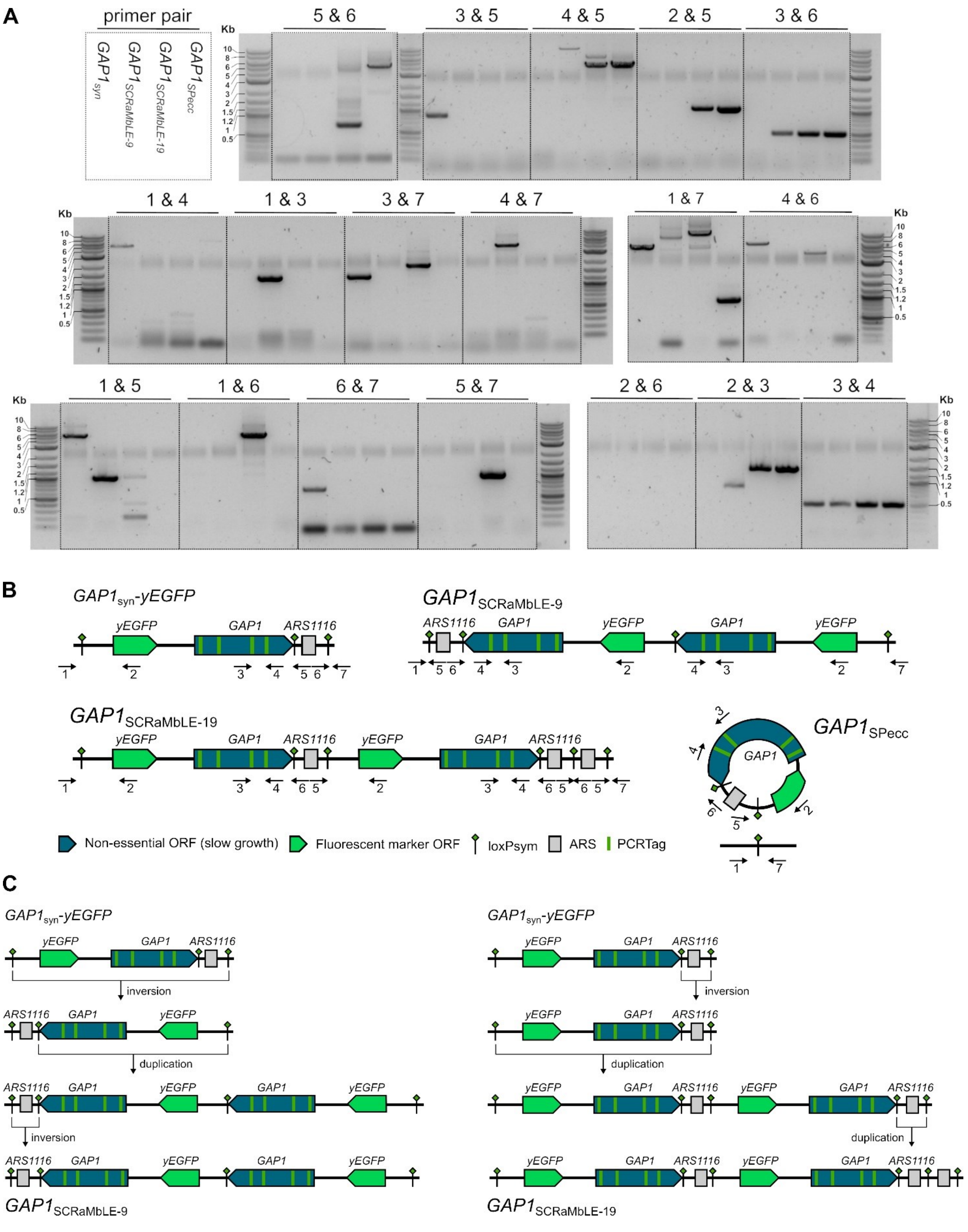

Figure S9: SCRaMbLE rearrangements of the *GAP1syn* locus were identified – related to Figure 5. (A) Agarose gel electrophoresis analysis of diagnostic PCRs to determine *GAP1* locus structure of strains following SCRaMbLE of the *GAP1syn-yEGFP* strain. Boxed sections of gel images denote samples with the same primer pairs, indicated above the boxes. Top left box shows lane loading order of each strain analysed in each boxed section. Primer pairs are numbered 1 = XL216, 2 = XL789, 3 = XL788, 4 = XL808, 5 = XL809, 6 = XL790, 7 = XL222. Primer sequences can be found in Table S3. Ladder is 1 kb Plus DNA Ladder (NEB). (B) Show's schematic overviews of the *GAP1* locus of *GAP1syn-yEGFP* and the SCRaMbLE derived strains. Numbered arrows are indicative of the binding positions and direction of each of the primers used in diagnostic PCR. Arrow lengths and relative positions are not to scale. (C) Shows one possible mechanism each for the SCRaMbLE generation of strains *GAP1*SCRaMbLE-9 and *GAP1*SCRaMbLE-19. As the diagnostic methods used cannot confirm the specific SCRaMbLE events leading to each configuration, these are illustrative only.

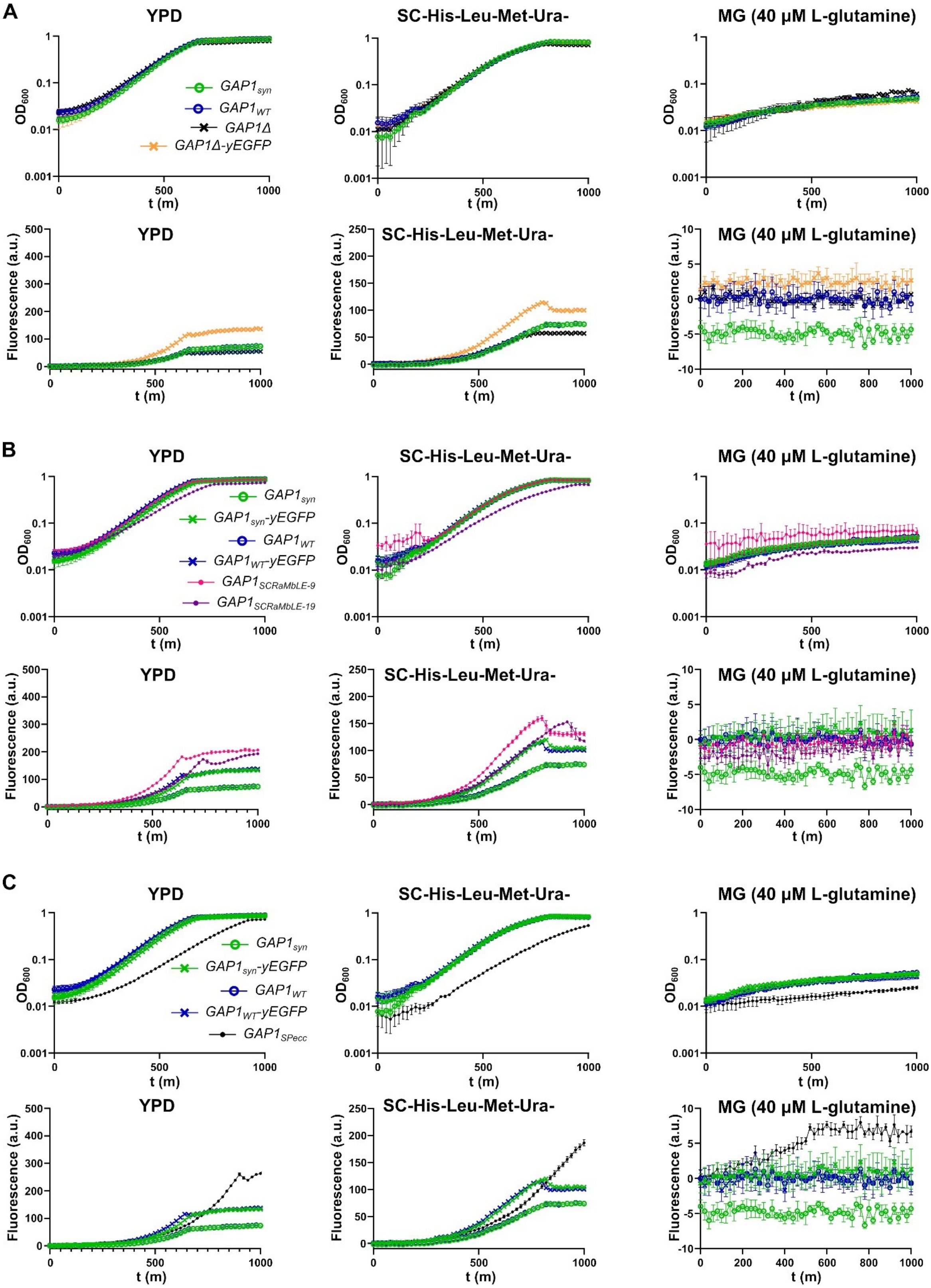

Figure S10: *$GAP1_{syn}$* locus variants were assessed for effects on growth - related to Figure 5. Growth and population fluorescence of BY4741 cultures with different *GAP1* locus variants. (A) shows *$GAP1_{syn}$, $GAP1_{WT}$, GAP1Δ* and *GAP1Δ-yEGFP cultures, (B)* shows *$GAP1_{syn}$, $GAP1_{syn}$-yEGFP*, *$GAP1_{WT}$, $GAP1_{WT}$-yEGFP*, *$GAP1_{SCRaMbLE-9}$ and $GAP1_{SCRaMbLE-19}$, and (C) shows $GAP1_{syn}$, $GAP1_{syn}$-yEGFP*, *$GAP1_{WT}$, $GAP1_{WT}$-yEGFP and $GAP1_{SPecc}$*. Media-blanked mean OD_{600} and fluorescence values from 3 biological replicates are plotted for each culture, error bars represent standard deviation. Assay growth medium is stated above each graph and assays took place at 30 °C. The y-axis has a log_{10} scale for OD_{600} and a linear scale for fluorescence.

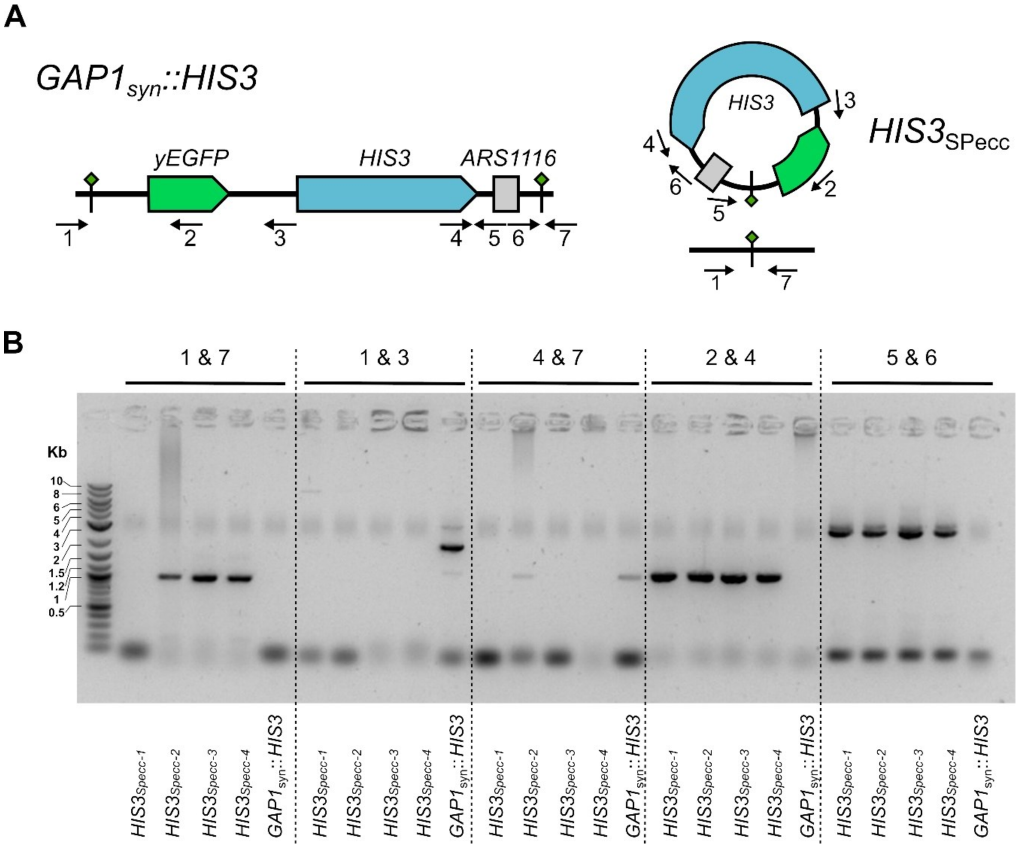

Figure S11: *HIS3* SPeccs were confirmed - related to Figure 6. (A) Show's schematic overviews of the *GAP1::HIS3* locus of BY4741-*HIS3-yEGFP* (labelled *GAP1*$_{syn}$::*HIS3*) and the SCRaMbLE derived HIS3$_{SPecc}$ strains. Numbered arrows are indicative of the binding positions and direction of each of the primers used in diagnostic PCR. Arrow lengths and relative positions are not to scale. (B) Agarose gel electrophoresis analysis of diagnostic PCRs to confirm *HIS3* SPecc formation following SCRaMbLE of the BY4741-*HIS3-yEGFP* strain. Primer pairs are indicated above the gel and strain analysed in each lane is indicated below the gel. Primer pairs are numbered 1 = XL216, 2 = XL789, 3 = XL1243, 4 = XL1244, 5 = XL809, 6 = XL790, 7 = BB585. Primer sequences can be found in Table S3. Ladder is 1 kb Plus DNA Ladder (NEB).

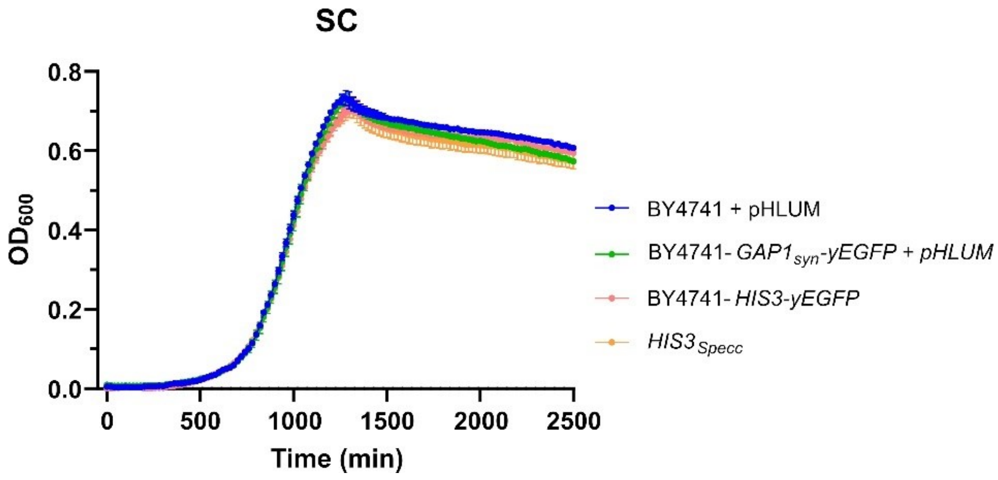

Figure S12: *HIS3* SPeccs do not affect growth in SC medium - related to Figure 6. Growth of BY4741-*HIS3*$_{SPecc}$ and parental strains in defined media with histidine (SC) at 30 °C. Mean OD_{600} values from 3 biological replicates are plotted as circles, error bars represent standard deviation.

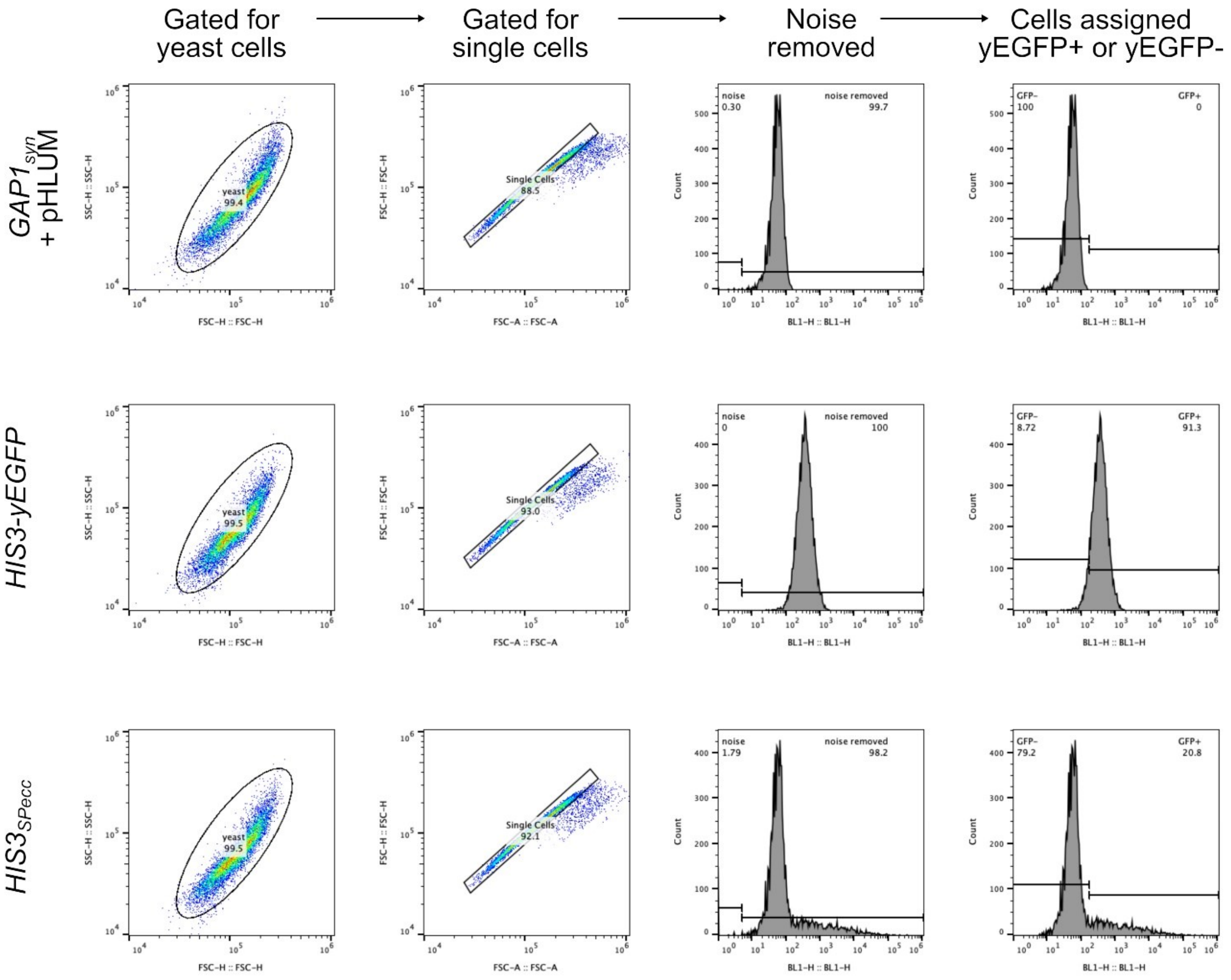

Figure S13: Gating distinguished the yEGFP status of cells – related to Figure 6. Examples of the gating process used to process raw flow cytometry data to generate the ratios shown in Figure 6F. Shown here is one set of data from each culture grown in SC (histidine +) medium.

Supplemental tables

Design element	Synthesized design *synXI*_3.34	Final sequence *synXI*_9.11
TAG stop codons replaced	68	71
PCRTags	914	900
loxP sites	199	196
tRNAs removed	15	15
introns removed	9	9
Assembly restriction sites	87	80
chunks - synthesis section	87	-
megachunks - integration sections	18	-
Assembled size (bp)*	659,617	659,107

Table S1 – Summary of design changes implemented in *synXI* – related to Figure 1. Asterisk denotes that this number does not take into account *in vivo* fluctuation in telomere sequence length.

Purpose	Target	Target sequence	Retargeting primer	Retargeting primer sequence	Repair template
M Debugging	M1 - *YKL005C_2_WT_R*	GGAAACGTCAAAGGAGGCAG[AGG]	BB392	CTGCCTCCTTTGACGTTTCCaaagtcccattcgccacccg	M1 chunk
M Debugging	M2 - *YKR001C_2_WT_F*	ACTGGCGCCTTACTAGACGA[TGG]	BB394	TCGTCTAGTAAGGCGCCAGTaaagtcccattcgccacccg	M2 chunk
M Debugging	M3 - *YKR006C_1_WT_R*	TATTGAAGAAGGCACTAACG[AGG]	BB396	CGTTAGTGCCTTCTTCAATAaaagtcccattcgccacccg	M3 chunk
M Debugging	M4 - *YKR010C_2_WT_F*	CTAGTGGTGCTCATAGTCCC[AGG]	BB398	GGGACTATGAGCACCACTAGaaagtcccattcgccacccg	M4 chunk
M Debugging	M5 - *YKR013W_1_WT_F*	TGAGGATTGAGTGCTTGAGG[CGG]	BB400	CCTCAAGCACTCAATCCTCAaaagtcccattcgccacccg	M5 chunk
*CEN11** replacement	*KlURA3* 3' region	CTTCGGGAATCGCCTATCAG[CGG]	BB875	CTGATAGGCGATTCCCGAAGaaagtcccattcgccacccg	*CEN11* variant BB877/BB878 PCR product
*CEN11** replacement	*YKR001C_2_WT_F*	ACTGGCGCCTTACTAGACGA[TGG]	BB394	TCGTCTAGTAAGGCGCCAGTaaagtcccattcgccacccg	M2 chunk
HIS3-TR2 removal	HIS3	TTTTTACTCCACGCGCCAGT[AGG]	BB417	ACTGGCGCGTGGAGTAAAAAaaagtcccattcgccacccg	*his3*Δ1 as BB418/BB419 PCR product
O integration patching	*YKR051W_1_WT_R*	ATTCTTGTACACATATCCAG[AGG]	BB390	CTGGATATGTGTACAAGAATaaagtcccattcgccacccg	O3-O4 chunk ligation product
R5 *URA3* removal	*URA3*	GCAGACATTACGAATGCGCA[CGG]	BB486	TGCGCATTCGTAATGTCTGCaaagtcccattcgccacccg	Neighbouring sequence
Mitotic crossover	O5 *LEU2*	GAAAAAAATAAAAATGGAGT[AGG]	BB457	ACTCCATTTTTATTTTTTTCaaagtcccattcgccacccg	Sister chromatid
J repeat condensation	*LEU2*	CCAGCGCCTCATCTGGAAGT[GGG]	BB573	ACTTCCAGATGAGGCGCTGGaaagtcccattcgccacccg	Megachunk J
J repeat condensation	*kanMX*	ATGAAGGAGAAAACTCACCG[AGG]	BB574	CGGTGAGTTTTCTCCTTCATaaagtcccattcgccacccg	Megachunk J
J repeat condensation	J1 vector backbone sequence (5')	ACTGGAAAGCGGGCAGTGAG[CGG]	BB604	CTCACTGCCCGCTTTCCAGTaaagtcccattcgccacccg	Neighbouring sequence
J repeat condensation	J1 vector backbone sequence (3')	CTATAGGGCGAATTGGCGGA[AGG]	BB603	TCCGCCAATTCGCCCTATAGaaagtcccattcgccacccg	Neighbouring sequence
Q repeat condensation	*URA3*	GCAGACATTACGAATGCGCA[CGG]	BB486	TGCGCATTCGTAATGTCTGCaaagtcccattcgccacccg	Megachunk Q
TRK2 insertion removal	*TRK2* insertion - transposon sequence	GCTCAAAATTTATTCACACA[TGG]	BB736	TGTGTGAATAAATTTTGAGCaaagtcccattcgccacccg	Neighbouring sequence
Q debugging	P5 *URA3*	GAAAAGCTTGCGGTAGTGAA[GGG]	BB783	TTCACTACCGCAAGCTTTTCaaagtcccattcgccacccg	Megachunk Q
Q debugging	*YKR077W_1_WT_R*	GTTTGCATTCGGAGTTCTTG[GGG]	BB704	CAAGAACTCCGAATGCAAACaaagtcccattcgccacccg	Megachunk Q

Purpose	Target	Target sequence	Retargeting primer	Retargeting primer sequence	Repair template
Q debugging	*YKR084C _2_WT_F*	AGAGTTGTTCTTA GAAAGGA[CGG]	BB705	TCCTTTCTAAGAACAACTCTaaagtcc cattcgccacccg	Megachunk Q
Glycerol locus replacement	*YKR084C _1_syn_F*	GCTAACGCCGATC AACGTAG[TGG]	BB757	CTACGTTGATCGGCGTTAGCaaagtc ccattcgccacccg	Debug colony 2 locus BB768/BB775 PCR product
Glycerol locus replacement	*YKR086W _1_syn_R*	CAATGGAGTTAAT TGACCTG[AGG]	BB778	CAGGTCAATTAACTCCATTGaaagtcc cattcgccacccg	Debug colony 2 locus BB768/BB775 PCR product
PRP16 stop swap	*PRP16* TAG	TCTTCCCATAAACT AAAAAA[AGG]	BB785	TTTTTTTAGTTTATGGGAAGaaagtccc attcgccacccg	Q3 chunk BB738/BB788 PCR product
GAP1$_{syn}$ generation	*YKRCδ11*	TCACCCATTTGTC AAGATAA[TGG]	XL498	TTTGGTCTCGCGCATCACCCATTTG TCAAGATAAGTTTTAGAGCTAGAAA TAGCAAGTTA	synthetic *GAP1* locus amplicon
			XL499	TTTGGTCTCGTTGATATAAGCCCTG CGCAAGCCCGGAATCGAAC	
GAP1$_{syn}$ generation	*YKRCδ12*	GGGCTTATATCAA GATCTGT[TGG]	XL500	TTTGGTCTCCTCAAGATCTGTGTTT TAGAGCTAGAAATAGCAAGTTA	synthetic *GAP1* locus amplicon
			XL501	TTTGGTCTCCGGATTGCGCAAGCC CGGAATCGAAC	
yEGFP insertion	sequence upstream of *GAP1*	TTTGTCGAAGATA TTCGACG[AGG]	XL502	AGATTTTGTCGAAGATATTCGACG	*PPFY1-yEGFP-TCYC1*
			XL503	AAACCGTCGAATATCTTCGACAAA	
Replacing *GAP1* with *HIS3*	sequence upstream of *GAP1*	GTTGTAGGTTACG TAAGCAG[GGG]	XL1176	TTTGGTCTCGCGCAGTTGTAGGTTA CGTAAGCAGGTTTTAGAGCTAGAAA TAGCAAGTTA	*HIS3*
			XL1182	TTTGGTCTCGTTAGATTAATGATGC GCAAGCCCGGAATCGAAC	
Replacing *GAP1* with *HIS3*	sequence downstrea m of *GAP1*	TCATTAATCTAAAG AAAAAA[GGG]	XL1177	TTTGGTCTCCCTAAAGAAAAAAGTT TTAGAGCTAGAAATAGCAAGTTA	*HIS3*
			XL1501	TTTGGTCTCCGGATTGCGCAAGCC CGGAATCGAAC	

Table S5 - CRISPR/Cas9 target sequences and gRNA retargeting primers – related to STAR methods. Target sequence Protospacer Adjacent Motifs are shown in square brackets. Retargeting primer sequences are shown with the pWS082 binding sequence in lowercase and the retargeting sequence in uppercase.

Version	Strain(s)	Comment	Details
*synXI*_3.34	N/A		Final design by BioStudio
*synXI*_3.36	N/A		TAG stop codons in *YKL006C-A*, *YKR004C* and *YKR005C* recoded to TAA
*synXI*_3.37	N/A		*CEN11* right arm loxPsym site moved to 3 bp 3' of *YKR001C*
*synXI*_9.01	ysXIb01	Initial synXI assembly	Missing loxPsym sites: 526668-526701 (*YKR052C*). WT PCRTags: 466241-466268 (*YKR016W*_1_F), 493836-493863 (*YKR029C*_2_R),493911-493938 (*YKR029C*_1_F), 536959-536986 (*YKR054C*_6_R). Point mutations causing amino acid changes: 37065 T>C (*YKL210W*), 448094 G>C (*YKR008W*). Structural variations: 303238-328404 (repeated sequence), 523727-525128 (insertions/duplication at *YKR050W*), 578748-591038 (repeated sequence).
*synX*I_9.02	ysXIb02	Markers inserted flanking megachunk J repeated sequence	Missing loxPsym sites: 526668-526701 (*YKR052C*). WT PCRTags: 466241-466268 (*YKR016W*_1_F), 493836-493863 (*YKR029C*_2_R),493911-493938 (*YKR029C*_1_F), 536959-536986 (*YKR054C*_6_R). Point mutations causing amino acid changes: 37065 T>C (*YKL210W*), 448094 G>C (*YKR008W*). Structural variations: 303238-328404 (repeated sequence), 523727-525128 (insertions/duplication at *YKR050W*), 578748-591038 (repeated sequence). Marker gene insertions: 303694 (*YKL0069W*::*LEU2*), 334347 (*YKL053C*::*kanMX4*).
*synXI*_9.03	ysXIb03	Megachunk J repeated sequence copy number reduced - respiratory growth defect present	Missing loxPsym sites: 526668-526701 (*YKR052C*). WT PCRTags: 466241-466268 (*YKR016W*_1_F), 493836-493863 (*YKR029C*_2_R),493911-493938 (*YKR029C*_1_F), 536959-536986 (*YKR054C*_6_R). Point mutations causing amino acid changes: 37065 T>C (*YKL210W*), 448094 G>C (*YKR008W*). Structural variations: 303238-311650 (repeated sequence with pMA-RQ insertion at 311649), 523727-525128 (insertions/duplication at *YKR050W*), 578748-591038 (repeated sequence). Marker gene insertions: 343787 (*YKL048C*::*URA3*).
*synXI*_9.04	ysXIb04	*URA3* marker removed - respiratory growth defect present	Missing loxPsym sites: 526668-526701 (*YKR052C*). WT PCRTags: 466241-466268 (*YKR016W*_1_F), 493836-493863 (*YKR029C*_2_R),493911-493938 (*YKR029C*_1_F), 536959-536986 (*YKR054C*_6_R). Point mutations causing amino acid changes: 37065 T>C (*YKL210W*), 448094 G>C (*YKR008W*). Structural variations: 303238-311650 (repeated sequence with pMA-RQ insertion at 311649), 523727-525128 (insertions/duplication at *YKR050W*), 578748-591038 (repeated sequence).
*synXI*_9.05	ysXIb05	Megachunk J repeated sequence removed - respiratory growth defect present	Missing loxPsym sites: 526668-526701 (*YKR052C*). WT PCRTags: 466241-466268 (*YKR016W*_1_F), 493836-493863 (*YKR029C*_2_R),493911-493938 (*YKR029C*_1_F), 536959-536986 (*YKR054C*_6_R). Point mutations causing amino acid changes: 37065 T>C (*YKL210W*), 448094 G>C (*YKR008W*). Structural variations: 523727-525128 (insertions/duplication at *YKR050W*), 578748-591038 (repeated sequence).
*synXI*_9.06	ysXIb06	*URA3* inserted upstream of megachunk Q repeated sequence - respiratory growth defect present	Missing loxPsym sites: 526668-526701 (*YKR052C*). WT PCRTags: 466241-466268 (*YKR016W*_1_F), 493836-493863 (*YKR029C*_2_R),493911-493938 (*YKR029C*_1_F), 536959-536986 (*YKR054C*_6_R). Point mutations causing amino acid changes: 37065 T>C (*YKL210W*), 448094 G>C (*YKR008W*). Structural variations: 523727-525128 (insertions/duplication at *YKR050W*), 578748-591038 (repeated sequence). Marker gene insertions: 579173 (*YKR077W*::*URA3*).
*synXI*_9.08	ysXIb08	Megachunk Q repeated sequence removed - respiratory growth defect present	Missing loxPsym sites: 526668-526701 (*YKR052C*). WT PCRTags: 466241-466268 (*YKR016W*_1_F), 493836-493863 (*YKR029C*_2_R),493911-493938 (*YKR029C*_1_F), 536959-536986 (*YKR054C*_6_R). Point mutations causing amino acid changes: 37065 T>C (*YKL210W*), 448094 G>C (*YKR008W*). Structural variations: 523727-525128 (insertions/duplication at *YKR050W*).
*synXI*_9.09	ysXIb09	*TRK2*/*YKR050W* insertions/duplication removed -	Missing loxPsym sites: 526668-526701 (*YKR052C*). WT PCRTags: 466241-466268 (*YKR016W*_1_F), 493836-493863 (*YKR029C*_2_R),493911-493938 (*YKR029C*_1_F), 536959-536986 (*YKR054C*_6_R). Point mutations causing

Version	Strain(s)	Comment	Details
		respiratory growth defect present	amino acid changes: 37065 T>C (*YKL210W*), 448094 G>C (*YKR008W*).
*synXI*_9.10	ysXIb10, ysXIb11, ysXIb12	Respiratory growth defect fixed	Remaining TAG stop codons: 597913 A>G (*YKR086W*). Missing loxPsym sites: 526668-526701 (*YKR052C*), 593898-593931 (*YKR085C*),598033-598066 (*YKR087C*). WT PCRTags: 466241-466268 (*YKR016W*_1_F), 493836-493863 (*YKR029C*_2_R),493911-493938 (*YKR029C*_1_F), 536959-536986 (*YKR054C*_6_R), 592963-592990 (*YKR084C*_1_F), 593239-593266 (*YKR084C*_1_R), 593956-593983 (*YKR085C*_1_F), 594292-594319 (*YKR085C*_1_R), 595453-595480 (*YKR086W*_2_F), 595684-595711 (*YKR086W*_2_R), 595912-595939 (*YKR086W*_3_F), 596266-596293 (*YKR086W*_3_R), 596749-596773 (*YKR086W*_1_F), 596989-597016 (*YKR086W*_1_R). Point mutations causing amino acid changes: 37065 T>C (*YKL210W*), 448094 G>C (*YKR008W*).
*synXI*_9.11	ysXIb13, ysXIb14, ysXIb16, ysXIb17	Final version	Missing loxPsym sites: 526668-526701 (*YKR052C*), 593898-593931 (*YKR085C*),598033-598066 (*YKR087C*). WT PCRTags: 466241-466268 (*YKR016W*_1_F), 493836-493863 (*YKR029C*_2_R),493911-493938 (*YKR029C*_1_F), 536959-536986 (*YKR054C*_6_R), 592963-592990 (*YKR084C*_1_F), 593239-593266 (*YKR084C*_1_R), 593956-593983 (*YKR085C*_1_F), 594292-594319 (*YKR085C*_1_R), 595453-595480 (*YKR086W*_2_F), 595684-595711 (*YKR086W*_2_R), 595912-595939 (*YKR086W*_3_F), 596266-596293 (*YKR086W*_3_R), 596749-596773 (*YKR086W*_1_F), 596989-597016 (*YKR086W*_1_R). Point mutations causing amino acid changes: 37065 T>C (*YKL210W*), 448094 G>C (*YKR008W*).

Table S6 - *synXI* chromosome versions – related to STAR methods. For *in vivo* chromosome versions, variations from *synXI*_3.37 are listed.
